# Frequency and impact of medication reviews for people aged 65 years or above in UK primary care: an observational study using electronic health records

**DOI:** 10.1186/s12877-023-04143-2

**Published:** 2023-07-14

**Authors:** Rebecca M. Joseph, Roger D. Knaggs, Carol A. C. Coupland, Amelia Taylor, Yana Vinogradova, Debbie Butler, Louisa Gerrard, David Waldram, Barbara Iyen, Ralph K. Akyea, Darren M. Ashcroft, Anthony J. Avery, Ruth H. Jack

**Affiliations:** 1 Centre for Academic Primary Care, Lifespan and Population Health, School of Medicine, University of Nottingham, Nottingham, UK; 2 National Institute for Health and Care Research Nottingham Biomedical Research Centre, Nottingham University Hospitals NHS Trust, Nottingham, UK; 3 School of Pharmacy, University of Nottingham, Nottingham, UK; 4 National Institute for Health and Care Research MindTech MedTech Co-operative, The Institute of Mental Health, Mental Health and Clinical Neurosciences, University of Nottingham, Nottingham, UK; 5 Centre for Pharmacoepidemiology and Drug Safety, Division of Pharmacy and Optometry, School of Health Sciences, Faculty of Biology, Medicine and Health, University of Manchester, Manchester, UK; 6 NIHR Greater Manchester Patient Safety Translational Research Centre, Manchester Academic Health Science Centre, University of Manchester, Manchester, UK

**Keywords:** Medication Review, Polypharmacy, General Practice, Older Adults, Routinely Collected Health Data

## Abstract

**Background:**

Medication reviews in primary care provide an opportunity to review and discuss the safety and appropriateness of a person’s medicines. However, there is limited evidence about access to and the impact of routine medication reviews for older adults in the general population, particularly in the UK. We aimed to quantify the proportion of people aged 65 years and over with a medication review recorded in 2019 and describe changes in the numbers and types of medicines prescribed following a review.

**Methods:**

We used anonymised primary care electronic health records from the UK’s Clinical Practice Research Datalink (CPRD GOLD) to define a population of people aged 65 years or over in 2019. We counted people with a medication review record in 2019 and used Cox regression to estimate associations between demographic characteristics, diagnoses, and prescribed medicines and having a medication review. We used linear regression to compare the number of medicines prescribed as repeat prescriptions in the three months before and after a medication review. Specifically, we compared the ‘prescription count’ - the maximum number of different medicines with overlapping prescriptions people had in each period.

**Results:**

Of 591,726 people prescribed one or more medicines at baseline, 305,526 (51.6%) had a recorded medication review in 2019. Living in a care home (hazard ratio 1.51, 95% confidence interval 1.40-1.62), medication review in the previous year (1.83, 1.69-1.98), and baseline prescription count (e.g. 5-9 vs 1 medicine 1.41, 1.37-1.46) were strongly associated with having a medication review in 2019. Overall, the prescription count tended to increase after a review (mean change 0.13 medicines, 95% CI 0.12-0.14).

**Conclusions:**

Although medication reviews were commonly recorded for people aged 65 years or over, there was little change overall in the numbers and types of medicines prescribed following a review. This study did not examine whether the prescriptions were appropriate or other metrics, such as dose or medicine changes within the same class. However, by examining the impact of medication reviews before the introduction of structured medication review requirements in England in 2020, it provides a useful benchmark which these new reviews can be compared with.

**Supplementary Information:**

The online version contains supplementary material available at 10.1186/s12877-023-04143-2.

## Introduction

The number of people living with multiple chronic conditions is increasing, and with it the number of people taking multiple medicines [1]. In 2015, the proportion of people aged 65 years or over taking five or more medicines ranged from 26% to 40% across 17 European countries [2]. Polypharmacy (use of multiple medicines) is associated with several adverse outcomes including increased risk of adverse drug reactions and other medicine-related problems, such as inappropriate prescribing [3–5], and a negative impact on adherence [6] and quality of life [7]. Ongoing prescribing of medicines that are no longer required or no longer appropriate is a problem recognised in the UK Department for Health and Social Care’s National Overprescribing Review, which recommends medication reviews as one tool for addressing inappropriate prescribing [8].

In the United Kingdom (UK), prescribers are expected to perform regular medication reviews for people prescribed repeat medicines. Between 2004 and 2013, English general practices were incentivised to perform medication reviews every 15 months for people prescribed repeat medicines as part of the national Quality and Outcomes Framework (QOF) [9], and medication review targets are still included for some conditions (e.g. heart failure [10]). In general practice, a typical medication review may involve a consultation with the patient or a review of medical records [11], and may be led by a general practitioner (GP), practice-based pharmacist, or other appropriate healthcare professional. Certain individuals may be offered a structured medication review, defined by the National Institute for Health and Care Excellence (NICE) as ‘a critical examination of a person’s medicines with the objective of reaching an agreement with the person about treatment, optimising the impact of medicines, minimising the number of medication‑related problems and reducing waste’ [12]. However, these structured medication reviews are targeted to specific populations [12, 13], and may not be the experience of many people prescribed long-term medicines.

As existing evidence focuses on structured medication reviews, there is little evidence about access to and the impact of typical medication reviews in the wider UK population. One cross-sectional study conducted in Sweden compared people with and without a recorded medication review. They found recorded medication reviews were more common in people with conditions including type 2 diabetes, hypertension, and depression, and where medication reviews were incentivised [14].

Typical medication reviews in general practice may be the only opportunity to check the appropriateness of medicines for large numbers of people. It is therefore important that there are no inequalities in access to these reviews, and that they can result in changes to prescribing where necessary. The current study explored some of these issues in a large population-based cohort of older adults. The study aims were 1) to quantify the numbers of people aged 65 years or over with a recorded medication review in 2019, 2) to test for differences in likelihood of having a medication review by person characteristics, and 3) to test for changes to the types and numbers of medicines prescribed before and after a medication review.

## Methods

### Design and data source

This was an observational study using electronic health records (EHR) to capture medication reviews recorded from 01 January 2019 to 31 December 2019. We focused on reviews occurring in 2019 as the last complete year before the introduction of the new Structured Medication Review guidelines [13]. We used data from the UK’s Clinical Practice Research Datalink (CPRD GOLD), a database of anonymised routinely collected primary care EHR. CPRD patients are representative of the UK population in terms of age and sex, and in 2013 it was estimated that 6.9% of the total UK population were included [15]. The data are captured as part of routine healthcare delivery and administration, and include demographic and lifestyle characteristics, symptoms and diagnoses, test results, prescriptions issued in primary care, and other clinical events. Clinical information is coded using Read codes (v2). We used the CPRD GOLD dataset (May 2022 build) linked with person-level and practice-level deprivation data (Townsend Score quintile [16], linkage based on post code).

### Study population

The study included people aged 65 years and over in 2019 with at least one year of ‘up-to-standard’ [15] (a marker of data quality) follow-up in CPRD before 01 January 2019. Individual patient follow-up was from 01 January 2019 to the earliest of death, leaving the practice, or last practice data collection date. Due to small numbers (*n=*13), we excluded people with missing/unclassified sex. For the analysis looking at numbers of people having a medication review, we excluded people with no ongoing prescriptions at baseline (defined below) and censored follow-up at first medication review or 31 December 2019. For the analyses comparing types and numbers of medicines prescribed before and after a medication review, we included only people who had a medication review in 2019 and at least three months’ follow-up after the review. When comparing numbers of medicines, we also excluded people with no prescriptions in the three months prior to their medication review, and with an extreme change in count (more than three standard deviations (SDs) from the mean – see later).

### Medication reviews

Medication reviews recorded during 2019 were identified using a pre-defined list of Read codes (see Additional File 1, table S1.1; also available online – see data sharing statement). The code list was developed by searching the code dictionary for relevant terms. The final list was approved by the research team, which includes practicing general practitioners (AJA, BI). The first medication review recorded in 2019 was the event of interest and any subsequent reviews are not included in the study. Medication reviews were classified according to the consultation type (face-to-face, telephone, other) and staff role (pharmacist, GP, nurse, admin, other) associated with each record (details in Additional File 1). As a sensitivity analysis, we used a conservative definition of medication reviews including only reviews recorded during face-to-face or telephone consultations, and excluding reviews for specific medicines/conditions, identified using Read codes (see Additional File 1, table S1.1).

### Prescribed medicines and prescription count

We derived the numbers and types of medicines prescribed at baseline, and in the three months before and three months after a medication review. Medicines were classified by drug substance and formulation, British National Formulary (BNF) [17] paragraph, and BNF chapter, and prescriptions were classified according to whether they were issued as a repeat prescription (yes/no). We excluded products in BNF chapters 14 (Immunological Products and Vaccines) and 15 (Anaesthesia), and non-medicinal products such as dressings, devices, and garments. The number of prescribed medicines (referred to as the ‘prescription count’) was defined as the number of unique medicines with prescriptions that overlapped for at least one day. The duration of each prescription was estimated using a similar approach to Pye et al. [18], using information about quantity prescribed and daily dose, and making different assumptions based on drug classes and formulations. Products with the same drug substance but different formulations were classified separately. The prescription count was categorised as 0, 1, 2-4, 5-9, 10-14, 15-20, and 20+ prescribed medicines. Further details are given in Additional File 1 and the data processing code is available online (see data sharing statement).

### Other variables

Baseline variables were defined with respect to 01 January 2019 and were modelled as binary or categorical variables, except age (continuous, and categorised into 10-year bands). These variables were: age in 2019, sex, ethnicity, deprivation (quintile of Townsend Score [16], person-level if available, otherwise practice-level), practice region (grouped as Scotland, Wales, Northern Ireland, London, rest of England), smoking status, alcohol intake, body mass index (BMI), having a medication review in the previous year, whether the person was living in a care home, diagnoses mentioned in the Structured Medication Review guidelines [13], the Quality and Outcomes Framework [19], or the New Medicine Service [20], and baseline medicines commonly associated with medication errors [21]. The diagnoses included were: atrial fibrillation, cancer, chronic kidney disease, chronic obstructive pulmonary disease (COPD), coronary heart disease, dementia, depression, anxiety, diabetes, epilepsy, heart failure, hypertension, hypothyroidism, learning disability, mental health disorder (schizophrenia, bipolar affective disorder, or other psychosis), obesity, osteoporosis, being on the palliative care pathway, peripheral arterial disease, rheumatoid arthritis, stroke or transient ischaemic attack (TIA), asthma, dyslipidaemia, gout, glaucoma, Parkinson’s disease, benign prostatic hyperplasia, urinary incontinence or retention, mobility problems, thrombosis or thrombophilia, and frailty (severe frailty, recent fall, recent fracture). Where available, we used existing Read code lists to define these variables. These were sourced from the ClinicalCodes repository [22], the HDR-UK Phenotype Library [23, 24], OpenCodelists [25], QOF business rules [26], and individual papers (see Additional File 1). Baseline medicines were considered present if prescribed on or in the six-months before 01 January 2019 and included: non-steroidal anti-inflammatory drugs (NSAIDs), aspirin/antiplatelet medicines, renin-angiotensin system drugs, diuretics, opioids including combination analgesics, antidepressants, antipsychotics, bisphosphonates, benzodiazepines and z-drugs, gabapentinoids, inhaled long-acting beta-antagonists and corticosteroids, lithium, and anticholinergic medicines. Finally, we created indicator variables for medicines with ongoing prescriptions at baseline (i.e., with start and stop dates spanning 01 January 2019), at BNF-chapter level. Further details about how we defined the variables in this paragraph are given in Additional File 1.

### Analysis – quantifying and describing people who had a medication review

We summarised the baseline characteristics of the population of people with at least one ongoing prescription on 01 January 2019. We assessed the association between baseline characteristics and having a medication review in 2019 using Cox regression in order to take follow-up time into account. We performed unadjusted models, age-sex adjusted models, and multivariable models including demographic and clinical characteristics, and tested the proportional hazards assumption by examining log-log plots of survival and comparing observed and predicted Kaplan Meier survival plots.

### Analysis – comparing medicines prescribed before and after a medication review

When comparing types of medicines, we included only medicines flagged as repeat prescriptions to reduce noise from short-course medicines (e.g., anti-infective medication), which were overrepresented when considering medicines prescribed only before or only after a review (see Additional File 3 Figure S3.4 for results including all medicines). For simplicity and readability, we refer to medicines only prescribed before a review as ‘stopped’, medicines only prescribed after a review as ‘started’, and medicines prescribed before and after a review as ‘continued’. We ranked the twenty medicines most frequently stopped and/or started after a medication review, and counted the numbers of people who stopped, started, or continued each medicine. These results are presented graphically.

To assess the change in maximum prescription count, we calculated the difference between maximum prescription count after the review and maximum prescription count before the review. We then excluded people with a change in count more than three SDs from the mean, reducing skew when breaking down results by original prescription count. After confirming the change in prescription count had a Normal distribution, overall (Additional File 2 Figure S2.1) and for the different levels of the categorical variables, we used multivariable-adjusted linear regression to test the association between change in prescription count and demographic characteristics, prescription count before the review, consultation type, and staff role.

A ‘missing’ category was used to summarise variables with missing values (smoking status, alcohol intake, BMI, ethnicity, consultation type, staff role). Of these variables, only consultation type and staff role were included in regression models (using the ‘missing’ category). All regression models accounted for practice-level clustering by specifying robust standard errors that allowed for intragroup correlation. Results are shown with 95% confidence intervals (CI) and exact *p*-values. Data preparation and analyses were performed using Stata MP v17.0. The Stata do-files to replicate the whole analysis are available online (see data sharing statement).

### Sensitivity analyses

We repeated all analyses using the conservative definition of medication reviews and assessed change in type and number of medicines prescribed before and after a review using different time windows: 6 months before and after, 3 months before vs 1-4 months after (in order to account for any gradual changes, such as tapering medicines before they were stopped), and 1 month before and after.

### Patient and participant involvement

The study team includes three patient and participant involvement representatives (DB, LG, DW) who helped define the study aims and who have provided insights on our study design, methods, and interpretation.

## Results

### Quantifying and describing people who had a medication review

**Fig. 1:**
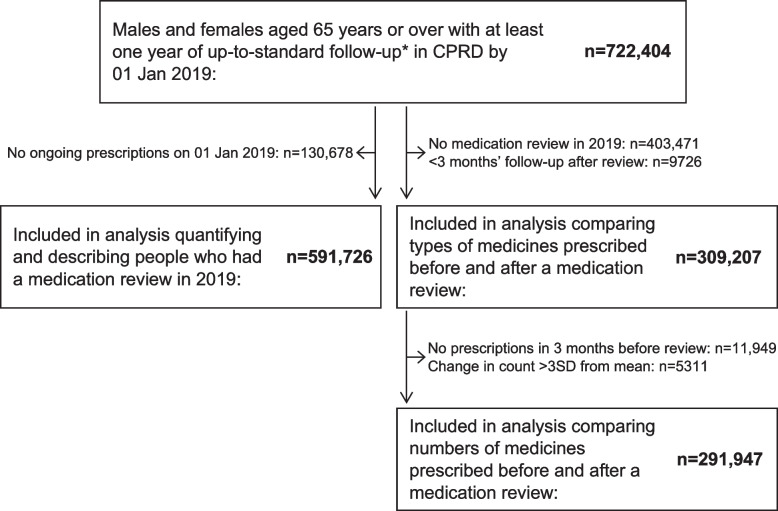
Flow diagram showing people included in each part of the study. CPRD Clinical Practice Research Datalink, n number of people, SD standard deviation. *a marker of data quality in CPRD

Figure 1 shows the number of people included in each part of the analysis. The original dataset contained 722,404 males and females aged 65 years and over with at least one year of up-to-standard follow-up prior to 01 January 2019 (baseline). After excluding 130,678 (18.1%) people with no ongoing prescriptions on 01 January 2019, 591,726 people remained in the study population for the first part of the analysis. The median age in this group was 74 years (interquartile range 70 to 81 years), and 54.5% were female. Key baseline characteristics are presented in Table 1, and further clinical details are presented in Additional File 2 Table S2.1.

**Table 1:** Characteristics of the study population as of 01 January 2019

	**Medication review in 2019**		
	**No**	**Yes**	**Whole study population**
**Overall count (number of people)**	286,200	305,526	591,726
**Age group (years) in 2019**			
65-74	149,168 (52.1%)	147,918 (48.4%)	297,086 (50.2%)
75-84	96,114 (33.6%)	110,534 (36.2%)	206,648 (34.9%)
85-94	36,970 (12.9%)	43,127 (14.1%)	80,097 (13.5%)
95+	3,948 (1.4%)	3,947 (1.3%)	7,895 (1.3%)
**Sex**			
Male	130,550 (45.6%)	138,847 (45.4%)	269,397 (45.5%)
Female	155,650 (54.4%)	166,679 (54.6%)	322,329 (54.5%)
**Ethnicity**			
Asian/British Asian	2,201 (0.8%)	2,184 (0.7%)	4,385 (0.7%)
Black/Black British	569 (0.2%)	484 (0.2%)	1,053 (0.2%)
Mixed	282 (0.1%)	229 (0.1%)	511 (0.1%)
Other	1,072 (0.4%)	1,033 (0.3%)	2,105 (0.4%)
White	143,017 (50.0%)	140,015 (45.8%)	283,032 (47.8%)
Missing	139,059 (48.6%)	161,581 (52.9%)	300,640 (50.8%)
**Townsend Score quintile**			
Quintile 1 (least deprived)	42,823 (15.0%)	44,588 (14.6%)	87,411 (14.8%)
Quintile 2	50,671 (17.7%)	52,117 (17.1%)	102,788 (17.4%)
Quintile 3	80,077 (28.0%)	87,105 (28.5%)	167,182 (28.3%)
Quintile 4	74,446 (26.0%)	79,073 (25.9%)	153,519 (25.9%)
Quintile 5 (most deprived)	38,183 (13.3%)	42,643 (14.0%)	80,826 (13.7%)
**Practice region**			
Scotland	101,940 (35.6%)	132,131 (43.2%)	234,071 (39.6%)
Wales	74,115 (25.9%)	94,792 (31.0%)	168,907 (28.5%)
Northern Ireland	23,214 (8.1%)	22,205 (7.3%)	45,419 (7.7%)
London	12,984 (4.5%)	6,523 (2.1%)	19,507 (3.3%)
Rest of England	73,947 (25.8%)	49,875 (16.3%)	123,822 (20.9%)
**Number of ongoing prescriptions at baseline**			
1	42,825 (15.0%)	24,454 (8.0%)	67,279 (11.4%)
2-4	114,980 (40.2%)	106,088 (34.7%)	221,068 (37.4%)
5-9	101,209 (35.4%)	130,820 (42.8%)	232,029 (39.2%)
10-14	23,437 (8.2%)	37,335 (12.2%)	60,772 (10.3%)
15-19	3,338 (1.2%)	6,028 (2.0%)	9,366 (1.6%)
20+	411 (0.1%)	801 (0.3%)	1,212 (0.2%)
**Medication review in previous year (2018)**	120,457 (42.1%)	207,071 (67.8%)	327,528 (55.4%)
**Living in a care home**	3,892 (1.4%)	7,569 (2.5%)	11,461 (1.9%)
**Medicines prescribed in the 6 months prior to baseline**			
NSAIDS (non-steroidal anti-inflammatory drugs)	21,485 (7.5%)	23,446 (7.7%)	44,931 (7.6%)
Oral anticoagulants	26,574 (9.3%)	39,351 (12.9%)	65,925 (11.1%)
Aspirin/antiplatelet medicines	67,694 (23.7%)	84,330 (27.6%)	152,024 (25.7%)
Renin-angiotensin system drugs	108,410 (37.9%)	133,328 (43.6%)	241,738 (40.9%)
Diuretics	64,065 (22.4%)	82,012 (26.8%)	146,077 (24.7%)
Opioids (including combination painkillers)	65,708 (23.0%)	83,406 (27.3%)	149,114 (25.2%)
Antidepressants	53,880 (18.8%)	71,215 (23.3%)	125,095 (21.1%)
Antipsychotics	4,803 (1.7%)	6,622 (2.2%)	11,425 (1.9%)
Bisphosphonates	16,805 (5.9%)	20,572 (6.7%)	37,377 (6.3%)
Benzodiazepines and Z-drugs	20,085 (7.0%)	26,171 (8.6%)	46,256 (7.8%)
Gabapentinoids	12,944 (4.5%)	19,134 (6.3%)	32,078 (5.4%)
Inhaled long-acting beta-antagonists and corticosteroids	36,131 (12.6%)	47,782 (15.6%)	83,913 (14.2%)
Lithium	470 (0.2%)	697 (0.2%)	1,167 (0.2%)
Anticholinergic medicines	49,935 (17.4%)	64,667 (21.2%)	114,602 (19.4%)

Results are count (column percent)

Out of the 591,726 people in this part of the analysis, 305,526 (51.6%) had at least one medication review in 2019. Of these, 73.2% had a single medication review. Considering only the first recorded medication reviews, 45.5% were recorded during face-to-face or telephone consultations and 67.2% were recorded by GPs (Table 2).

**Table 2:** Characteristics of first medication reviews recorded per person in 2019

	**Count**	**Percent**
**Consultation type**		
Face to face	129,678	42.4%
Telephone	9,600	3.1%
Other	166,056	54.4%
Missing	192	0.1%
**Staff role**		
Pharmacist	48,930	16.0%
GP (general practitioner)	205,296	67.2%
Nurse	25,954	8.5%
Other	16,689	5.5%
Admin	3,873	1.3%
Missing	4,784	1.6%

Results of unadjusted, age-sex-adjusted, and multivariable-adjusted Cox regression models are shown in Table 3 (full results for the models are given in Additional File 2 Table S2.2). In the unadjusted and age-sex adjusted models, most baseline diagnoses and prescribed medicines were associated with an increased likelihood of having a medication review (Additional File 2 Table S2.2). For diagnoses and prescribed medicines, the largest associations with having a medication review were for atrial fibrillation (age-sex adjusted hazard ratio (HR) 1.29, 95% CI 1.25 to 1.33) and antipsychotics (age-sex adjusted HR 1.38, 95% CI 1.32 to 1.44), respectively. In the fully adjusted model, living in a care home (HR 1.51, 95% CI 1.40 to 1.62), medication review in the previous year (HR 1.83, 95% CI 1.69 to 1.98), and increasing prescription count at baseline (HR for 5-9 medicines vs 1 medicine 1.41, 95% CI 1.37 to 1.46) were most strongly associated with having a review (Table 3). There was only a weak association between older age and having a medication review (HR for 75-84 vs 65-74 years 1.03, 95% CI 1.02 to 1.05), and no association for sex (HR 1.00, 95% CI 0.99 to 1.01 for male vs female). There were differences according to UK region, with people at practices in London (HR 0.62, 95% CI 0.47 to 0.81) and the rest of England (HR 0.77, 95% CI 0.65 to 0.91) less likely to have a recorded medication review than Scotland (the largest group in this case).

**Table 3:** Association between baseline characteristics and having a medication review in 2019, Cox regression

	**Hazard ratios (95% confidence intervals), *p*-value**		
	**Unadjusted**	**Age-sex adjusted**	**Fully adjusted**^**a**^
Age group, years (vs 65-74)			
75-84	1.13 (1.11, 1.15), *p<*0.001	1.13 (1.11, 1.15), *p<*0.001	1.03 (1.02, 1.05), *p<*0.001
85-94	1.22 (1.19, 1.26), *p<*0.001	1.22 (1.19, 1.26), *p<*0.001	1.04 (1.01, 1.06), *p=*0.006
95+	1.26 (1.20, 1.32), *p<*0.001	1.26 (1.20, 1.33), *p<*0.001	1.02 (0.97, 1.07), *p=*0.516
Female vs male	1.01 (0.99, 1.02), *p=*0.315	0.99 (0.98, 1.01), *p=*0.304	1.00 (0.99, 1.01), *p=*0.781
Townsend quintile (vs 1 – least deprived)			
Quintile 2	0.99 (0.81, 1.21), *p=*0.935	0.99 (0.81, 1.22), *p=*0.938	0.94 (0.80, 1.11), *p=*0.477
Quintile 3	1.03 (0.85, 1.25), *p=*0.756	1.03 (0.85, 1.25), *p=*0.754	0.97 (0.84, 1.12), *p=*0.678
Quintile 4	1.02 (0.82, 1.26), *p=*0.891	1.02 (0.82, 1.26), *p=*0.870	0.96 (0.81, 1.13), *p=*0.594
Quintile 5 - most deprived	1.04 (0.84, 1.30), *p=*0.700	1.05 (0.84, 1.30), *p=*0.669	0.98 (0.82, 1.15), *p=*0.776
Practice region (vs Scotland)			
Wales	1.01 (0.87, 1.17), *p=*0.904	1.01 (0.87, 1.17), *p=*0.942	0.99 (0.87, 1.13), *p=*0.913
Northern Ireland	0.82 (0.64, 1.06), *p=*0.131	0.82 (0.64, 1.06), *p=*0.128	0.81 (0.65, 1.01), *p=*0.064
London	0.54 (0.39, 0.74), *p<*0.001	0.53 (0.38, 0.74), *p<*0.001	0.62 (0.47, 0.81), *p=*0.001
Rest of England	0.72 (0.58, 0.88), *p=*0.001	0.71 (0.58, 0.87), *p=*0.001	0.77 (0.65, 0.91), *p=*0.003
Number of ongoing prescriptions at baseline (vs 1)			
2-4	1.45 (1.42, 1.49), *p<*0.001	1.44 (1.41, 1.48), *p<*0.001	1.27 (1.24, 1.30), *p<*0.001
5-9	1.88 (1.81, 1.95), *p<*0.001	1.84 (1.77, 1.92), *p<*0.001	1.41 (1.37, 1.46), *p<*0.001
10-14	2.25 (2.14, 2.37), *p<*0.001	2.20 (2.09, 2.32), *p<*0.001	1.48 (1.43, 1.55), *p<*0.001
15-19	2.51 (2.35, 2.67), *p<*0.001	2.46 (2.31, 2.63), *p<*0.001	1.51 (1.44, 1.59), *p<*0.001
20+	2.83 (2.56, 3.13), *p<*0.001	2.81 (2.54, 3.11), *p<*0.001	1.64 (1.49, 1.80), *p<*0.001
Medication review in previous year	2.05 (1.89, 2.21), *p<*0.001	2.03 (1.88, 2.20), *p<*0.001	1.83 (1.69, 1.98), *p<*0.001
Living in a care home	1.98 (1.83, 2.15), *p<*0.001	1.87 (1.73, 2.03), *p<*0.001	1.51 (1.40, 1.62), *p<*0.001

^a^Adjusted for variables shown plus baseline diagnoses and clinical indicators, and medicines prescribed in 6 months prior to baseline (see Additional File 2 Table S2.2 for the complete list)

In a sensitivity analysis using the conservative definition of medication reviews, 142,576 people (24.1% of the eligible population) had a medication review in 2019. The Cox regression results were broadly similar to the main analysis (Additional File 2 Table S2.3). One notable difference was in the association with practice region: compared to Scotland, people at practices in Wales were more likely to have a recorded medication review (HR 1.29, 95% CI 1.10 to 1.50) and people at practices in Northern Ireland less likely (HR 0.66, 95% CI 0.49 to 0.90). There was no significant difference between people at practices in London or the rest of England and people at practices in Scotland.

### Medicines prescribed before and after medication reviews

As shown in Fig. 1, 309,207 people who had a medication review in 2019 and at least three months of follow-up after their medication review were included in this part of the analysis. Characteristics of these people are summarised in Additional File 2, Table S2.4.

**Fig. 2:**
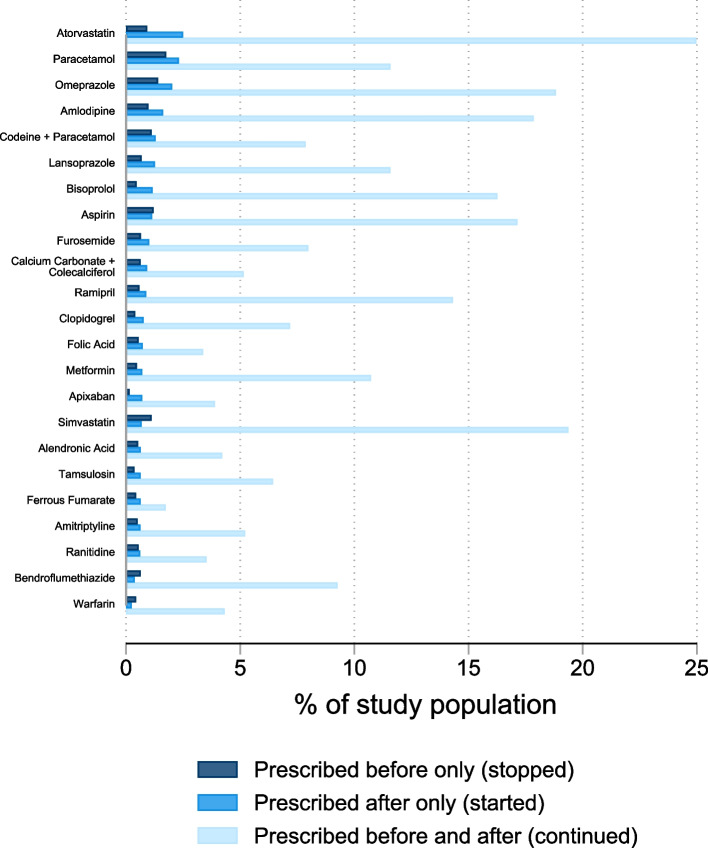
Medicines most frequently started or stopped after a medication review. These results are sorted by percentage started, are based on a study population of 309,207 people, and include only tablets prescribed as a repeat prescription. The time periods of interest are the three months before and three months after a medication review

Figure 2 shows the twenty medicines most frequently stopped or started in the three months after compared to three months before a medication review (see Additional File 2 Figure S2.5 for the corresponding statistics). This figure is restricted to tablets, but a version including all formulations is provided in Additional File 3. For the medicines shown, most people had prescriptions both before and after a medication review. From this list, four medicines were slightly more frequently stopped than started (aspirin, simvastatin, bendroflumethiazide, and warfarin). The other medicines shown were more often started than stopped. Some medicines made the list due to being prescribed to large numbers of people at any time (e.g., atorvastatin, omeprazole, simvastatin). Other medicines were prescribed to fewer people in total, but a higher proportion of people started or stopped them compared to other medicines. These included vitamins/supplements (e.g., colecalciferol, folic acid, ferrous fumarate), alendronic acid, ranitidine, apixaban, and warfarin. Additional figures showing results for sensitivity analyses, plus breakdowns at BNF chapter level and for named drug groups, are provided in Additional File 3. A sensitivity analysis using the strict definition of medication reviews had very similar results to the main analysis (Additional Figure 3 S3.12). Similarly, increasing the time window around the reviews did not have a big impact on the findings. With the shorter window (one month before and after the review), the proportions of people who stopped or started individual medicines were notably larger (likely reflecting refill frequencies slightly longer than one month).

When comparing the change in maximum prescription count, we further excluded people with no prescriptions in the three months before the review (*n=*11,949), and people with a change in maximum prescription count more than three standard deviations from the mean (*n=*5311), meaning 291,947 people remained in the analysis. The median (interquartile range) maximum prescription count was the same before and after a review (5, 3 to 8 medicines). The mean difference in maximum prescription count after compared with before a review was 0.13 (95% CI 0.12 to 0.14, *P<*0.001), indicating a small but statistically significant increase in count. Categorised prescription count before/after a review is summarised in Additional File 2, Table S2.6. Table 4 shows the results of multivariable linear regression adjusting for demographic characteristics and features of the medication review. Most factors had very small associations with change in prescription count. On average, people with a maximum prescription count of 10 or more medicines in the three months before a review had a lower count after the review (represented graphically in Additional File 2 Figure S2.2).

**Table 4:** Multivariable-adjusted linear regression for change in maximum prescription count

	**Coefficient (95% CI), *p*-value**
Age group (years)	
65-74	reference
75-84	0.04 (0.03, 0.05), *p<*0.001
85-94	0.04 (0.03, 0.06), *p<*0.001
95+	-0.03 (-0.07, 0.02), *p=*0.270
Sex	
Male	reference
Female	-0.01 (-0.02, -0.01), *p=*0.002
Original prescription count	
1	0.30 (0.28, 0.32), *p<*0.001
2-4	0.14 (0.13, 0.15), *p<*0.001
5-9	reference
10-14	-0.21 (-0.22, -0.19), *p<*0.001
15-19	-0.42 (-0.47, -0.38), *p<*0.001
20-24	-0.55 (-0.69, -0.40), *p<*0.001
Practice region	
Scotland	reference
Wales	0.05 (0.03, 0.07), *p<*0.001
Northern Ireland	0.12 (0.09, 0.14), *p<*0.001
London	0.07 (0.00, 0.13), *p=*0.058
Rest of England	0.02 (0.00, 0.05), *p=*0.031
Consultation type	
Face-to-face	reference
Telephone	-0.01 (-0.04, 0.03), *p=*0.674
Other	-0.02 (-0.04, -0.01), *p<*0.001
Missing	0.04 (-0.15, 0.22), *p=*0.715
Staff role	
General practitioner	reference
Pharmacist	-0.05 (-0.07, -0.03), *p<*0.001
Nurse	0.00 (-0.02, 0.02), *p=*0.863
Other	-0.04 (-0.06, -0.01), *p=*0.008
Admin	0.02 (-0.01, 0.06), *p=*0.207
Missing	0.01 (-0.10, 0.12), *p=*0.804
Townsend Quintile	
Quintile 1 (least deprived)	reference
Quintile 2	0.02 (-0.01, 0.06), *p=*0.208
Quintile 3	0.01 (-0.02, 0.04), *p=*0.380
Quintile 4	0.02 (-0.02, 0.05), *p=*0.306
Quintile 5 (most deprived)	0.03 (0.00, 0.07), *p=*0.056
Intercept	0.06 (0.02, 0.09), *p=*0.001

The outcome is change in maximum prescription count in the three months after vs three months before a medication review. Negative coefficients indicate a decrease in prescription count after the review, and vice versa. The model is adjusted for the factors shown in the table. CI confidence interval

A sensitivity analysis using the strict definition of medication reviews, which included 136,139 people, found similar results, with a mean difference in maximum prescription count after compared with before the review of 0.15 (95% CI 0.14 to 0.16, *P<*0.001). Increasing the time window around the medication review did not affect the results (six months pre- and post-review, mean change 0.17 (95% CI 0.16 to 0.17, *P<*0.001), three months pre- and one to four months post-review, mean change 0.09 (95% CI 0.08 to 0.10, *P<*0.001)). However, with a shorter time window (one month pre- and post-review), the mean change in prescription count was -0.48 (95% CI -0.54 to -0.42, *P<*0.001), indicating a decrease in count on average.

## Discussion

Approximately half (51.6%) of people aged 65 years or older who had at least one ongoing prescription at baseline had a medication review recorded in 2019. Living in a care home, baseline prescription count, and having a medication review in the previous year were the strongest predictors of having a medication review in 2019. There was little overall change in terms of both the types and the numbers of medicines prescribed in the three months before and after a medication review. On average, the maximum prescription count tended to increase slightly after a review. However, this varied according to the number of medicines prescribed before a review, and the average maximum count decreased in those prescribed 10 or more medicines before the review.

Although just over half of the people in the study had some form of medication review, only 45.5% of reviews were during consultations with patients (face-to-face or by telephone). No differences were observed in the likelihood of having a medication review in terms of age, sex, and deprivation, but we found differences according to practice region. This may reflect different healthcare policies in each of the four countries in the UK (England, Northern Ireland, Scotland, and Wales). We were unable to draw any useful conclusions regarding differences by ethnicity as the proportion of missing data was too high. Other primary care datasets might have more complete ethnicity data, and this could be explored for future studies. There is limited existing research into the routine provision of primary care medication reviews for older adults in the UK general population. Our study provides evidence that medication reviews are being recorded in primary care, with higher rates in more complex patients (e.g., people living in care homes, and people with higher prescription counts).

Comparing the medicines prescribed before and after a medication review, there was little change overall in the types and numbers prescribed. For the whole study population, the number of medicines increased on average, and although the difference was small (mean increase of 0.13 medicines), it would add up to large numbers of new medicines on a population scale. However, we cannot directly attribute the change to the medication review based on this study. The only factor having a notable impact on the number of medicines prescribed was the original prescription count: people prescribed more medicines before the review were more likely to have a reduced prescription count after the review, and vice versa. Trials and meta-analyses have shown that interventions similar to structured medication reviews tend to lead to a reduction in the total number of medicines prescribed [27–30]. In contrast, our study explored the impact of typical medication reviews delivered in a real-world setting on prescribed medicines. This study was not designed to assess the appropriateness of prescribing, and it is possible that no changes were required to the majority of prescriptions. Our results suggest that the medication reviews captured in this study, recorded in the primary care setting, do not necessarily lead to reductions in the numbers of medicines prescribed at the population-level, although they may have an impact for people prescribed a greater number of medicines. A different approach may be needed if implementation of the National Overprescribing Review [8] is to be successful. NHS England’s investment in pharmacists in primary care to undertake structured medication reviews may help with this, although this will need to be evaluated by future studies.

This study focused on the change in types and numbers of prescribed medicines. Further research could study other outcomes, including other changes to prescribed medicines (e.g., changes in dose or formulation, changing medicines within the same class, or reductions in potentially inappropriate prescribing), clinical outcomes such as hospitalisation rates, and outcomes important to patients, such as side-effects and problems with adherence [7, 31]. Considering changes to prescribed medicines, it would be useful to compare our results to a group of people who did not have a medication review over the same time frame. As our study was set before the introduction of requirements for targeted structured medication reviews [13], our results could be used as a baseline to review the impact of this change.

This was a large, population-based study, representing real-world practice. Although some regions (e.g., the East Midlands) were not represented in the study dataset, the results should be broadly generalisable to the UK population. The dataset provides an almost complete record of prescriptions issued in primary care. We grouped medicines by drug substance and formulation, allowing us to count unique medicines.

The limitations of the study include lack of detail about the medication reviews captured in the dataset. Due to the nature of the dataset, medication reviews were defined using a pre-specified list of clinical codes and while we could group the reviews by consultation type and staff role, we have no detail about whether these were brief medical record reviews or an in-depth discussion between clinician and patient. The study is unlikely to include medication reviews occurring outside general practice, such as in community pharmacies or secondary care. In addition, there is likely to be some misclassification in our definition of prescription count, which required us to estimate a prescription duration for the full range of prescribed medicines. This may particularly affect non-tablet formulations (e.g., inhalers, creams) and medicines used ‘as required’. However, we know the prescriptions included were issued within a similar time frame (+/- three months of the medication review) and our results should be comparable to other methods of defining polypharmacy (e.g., total number of medicines prescribed in a fixed time window). The dataset does not include medicines obtained outside primary care, including secondary care prescriptions and over-the-counter medicines. Finally, in common with other studies using routinely collected data, there may be misclassification in all variables due to missing, incomplete, or inaccurate information, which could introduce a degree of imprecision in our estimates.

## Conclusions

We found 51.6% of people aged 65 years or older with at least one ongoing prescription at baseline had a medication review recorded in 2019. We found no important differences in likelihood of having a medication review by several demographic characteristics; however, there was regional variation. On average, for the whole study population, there was a small increase in the maximum number of medicines prescribed after a review. This differed by prescription count before the review, with people prescribed more medicines before the review more likely to have a decrease in count after the review. Overall, we found little change in the type of medicines prescribed before and after a review. The results provide some reassurance that medication reviews are being recorded in UK primary care and may be useful in reducing the number of medicines for people with a large number of prescribed medicines. Further research is needed to evaluate the impact of medication reviews on other aspects of prescribing practice and medicine use.

## Supplementary Information

**Additional file 1:** **Additional Table S1.1.** Medication review Read codes. **Additional Table S1.2.** Staff role groupings. **Additional Table S1.3.** Consultation type groupings. **Additional Table S1.4.** Terms used to define specified drug groups.

**Additional file 2:** **Additional Table S2.1.** Full baseline characteristics of the study population as of 01 January 2019. **Additional Table S2.2.** Full results, association between baseline factors and having a medication review in 2019, Cox regression. **Additional Table S2.3.** Sensitivity analysis: association between baseline factors and having a medication review in 2019, Cox regression using conservative definition of medication reviews. **Additional Table S2.4.** Characteristics and mean change in prescription count for people with a medication review and at least three months of subsequent follow-up. **Additional Table S2.5.** Top twenty medicines most frequently stopped or started after a medication review. **Additional Figure S2.1.** Distribution of change (difference) in prescription count before (A) and after (B) dropping extreme values. **Additional Table S2.6.** Cross-tabulation of maximum prescription count before vs after a medication review. **Additional Figure S2.2.** Bar charts showing the mean change in prescription count by demographic and other characteristics.

**Additional file 3:** **Additional Figure S3.1.** Medicines prescribed in the three months before and/or after a medication review – BNF paragraph-level. **Additional Figure S3.2.** Medicines prescribed in the three months before and/or after a medication review – BNF chapter-level. **Additional Figure S3.3.** Medicines prescribed in the three months before and/or after a medication review – all formulations. **Additional Figure S3.4.** Medicines prescribed in the three months before and/or after a medication review – all prescription types. **Additional Figure S3.5.** Medicines prescribed in the three months before and/or after a medication review, by BNF chapter. **Additional Figure S3.6.** Psychotropic medicines prescribed in the three months before and/or after a medication review. **Additional Figure S3.7.** Opioids prescribed in the three months before and/or after a medication review. **Additional Figure S3.8.** Anticholinergic medicines prescribed in the three months before and/or after a medication review. **Additional Figure S3.9.** Gabapentinoids prescribed in the three months before and/or after a medication review. **Additional Figure S3.10.** Medicines prescribed in the six months before and/or after a medication review. **Additional Figure S3.11.** Medicines prescribed in the three months before and/or one-four months after a medication review. **Additional Figure S3.12.** Medicines prescribed in the one month before and/or after a medication review. **Additional Figure S3.13.** Medicines prescribed in the three months before and/or after an in-person medication review. **Additional Figure S3.14.** Medicines prescribed in the three months before and/or after a medication review – most frequently prescribed. **Additional Figure S3.15.** Medicines prescribed in the three months before and/or after a medication review – most frequently prescribed, BNF paragraph-level. **Additional Figure S3.16.** Medicines prescribed in the three months before and/or after a medication review – most frequently prescribed, BNF chapter-level.

## Data Availability

The data that support the findings of this study are not publicly available. They were provided by the Clinical Practice Research Datalink (CPRD) under license and according to CPRD’s data governance process. Researchers wishing to access the data must apply to CPRD, and requests are subject to protocol approval (https://cprd.com/data-access). Queries about the study data and results should be directed to the corresponding author (RHJ). The process for cleaning and analysing the data (including code lists and statistical code) is documented in full and is openly available through Zenodo.org (https://doi.org/10.5281/zenodo.7738103).

## Abbreviations

NIHR: National Institute for Heath and Care Research
UK: United Kingdom
NICE: National Institute for Health and Care Excellence
EHR: Electronic health records
CPRD: Clinical Practice Research Datalink
GP: General practitioner
BNF: British National Formulary
BMI: Body mass index
QOF: Quality and Outcomes Framework
COPD: Chronic obstructive pulmonary disorder
TIA: Transient ischaemic attack
HDR-UK: Health Data Research UK
NSAID: Non-steroidal anti-inflammatory drug
SD: Standard deviation
CI: Confidence interval
HR: Hazard ratio
n: Number
