# Supplementary material for: Frequency and impact of medication reviews for people aged 65 years or above in UK primary care: an observational study using electronic health records
Source: BMC Geriatr. 2023 Jul 14;23:435. doi: 10.1186/s12877-023-04143-2 (PMC10347807; doi:10.1186/s12877-023-04143-2)
Supplement: Supplementary file 1 — Additional file 1: Additional Table S1.1. Medication review Read codes. Additional Table S1.2. Staff role groupings. Additional Table S1.3. Consultation type groupings. Additional Table S1.4. Terms used to define specified drug groups. [file 12877_2023_4143_MOESM1_ESM.docx]

Additional File 1

Additional methods

Contents

[Additional Methods S1. Products included in/excluded from the analysis. 2](#_Toc129620643)

[Additional Methods S2. Estimating prescription duration. 3](#_Toc129620644)

[Additional Figure S1.1. Defining maximum prescription count. 5](#_Toc129620645)

[Additional Table S1.1. Medication review Read codes. 6](#_Toc129620646)

[Additional Table S1.2. Staff role groupings. 7](#_Toc129620647)

[Additional Table S1.3. Consultation type groupings. 8](#_Toc129620648)

[Additional Methods S3. Definitions of other variables 9](#_Toc129620649)

[References 12](#_Toc129620650)

# Additional Methods S1. Products included in/excluded from the analysis.

Sources of medicine/product information:

- CPRD dictionaries/lookup files (product, common_dosage)
- CPRD prescription dataset (Therapy files)
- SNOMED-BNF code mapping file (BNF Snomed Mapping data 20220617) available from the [NHS Business Services Authority](https://www.nhsbsa.nhs.uk/prescription-data/understanding-our-data/bnf-snomed-mapping) website
- The DM+D dictionary (NHSBSA dm+d 8.4.0 2021-08-30) available from the [NHS Digital TRUD](https://isd.digital.nhs.uk/trud/user/guest/group/0/home) website

Variables defined for each product in the CPRD dictionary:

- Drug name
- Formulation (drops, spray, inhaled, injected, creams/topical, patches, tablets, unspecified liquid, other, and non-drug)
- BNF code (six digit, BNF paragraph level)
- Repeat prescription (yes/no)
- Product to be used as required (yes/no)

Products excluded from the analysis:

- Products with formulation non-drug (includes dressings, devices, garments, food, and nutritional preparations)
- Homeopathic remedies, bath/shower products
- The following BNF chapters/sections:
  - 3.1.5 ‘Peak flow meters, inhaler devices and nebulisers’
  - 6.1.1.3 ‘Hypodermic equipment’
  - 6.1.6 ‘Diagnostic and monitoring devices for diabetes mellitus’
  - 7.3.4 ‘Contraceptive devices’
  - 7.4.4 ‘Bladder instillations and urological surgery’
  - 9.4 ‘Oral nutrition’
  - 14 ‘Immunological products and vaccines’
  - 15 ‘Anaesthesia’
  - Most products with BNF codes outside the 15 standard chapters (items identifiable as medicines are retained).

# Additional Methods S2. Estimating prescription duration.

The CPRD GOLD prescription data have an issue date (eventdate) but no stop date. There are several variables that can be used to estimate a prescription duration (including qty/daily_dose, numdays, dose_duration). For each prescription record, we defined the start date as the prescription issue date, and the stop date as the start date + estimated duration.

Below is a description of the process we developed to estimate the duration of all prescriptions in the Therapy dataset. The process has different rules depending on whether or not the prescription has the instruction ‘use as required’ (defined in an earlier step). Similar to the approach used in the [Manchester DrugPrep algorithm](https://doi.org/10.1002/pds.4440), it is possible to change some of the parameters of the process. We varied these parameters for different drug types/formulations as summarised in the following table.

|  | Oral glucocorticoids | Antibiotics (tablets) | Antibiotics (non-tablets) | Other products (tablets) | Other products (non-tablets) |
| --- | --- | --- | --- | --- | --- |
| **maxduration** | 365 | 365 | 365 | 365 | 365 |
| **dropqty** | no | no | yes | no | yes |
| **maxdifference** | 28 | 28 | 28 | 28 | 28 |
| **prndefault** | 7 | 7 | 7 | 28 | 28 |
| **default** | 7 | 7 | 7 | 28 | 28 |
| **overlap** | 14 | 14 | 14 | 14 | 14 |
| **maxgap** | 14 | 14 | 14 | 14 | 14 |

**Part 1) Data cleaning**

- Drop duplicate observations (all variables)
- Set qty and daily_dose to missing if qty>99^th^ centile
- Create
  - duration1 = median time between prescription refills (repeat prescriptions only)
  - duration2 = qty / daily_dose *Quantity ÷ daily dose (extracted from dosage text)*
  - duration3 = numdays *Duration sometimes entered by prescriber*
  - duration4 = dose_duration *Duration extracted from dosage text*
- For duration1 to duration4: set to missing if <1 or >[**maxduration**]
- [**optional: dropqty**] remove variable duration2 so not used to estimate duration

**Part 2) Estimate duration for each record**

- If record has dosage instruction ‘use as required’
  - use mean of duration3 and duration4 if the difference between them is <[**maxdifference**]
  - otherwise, use duration1
  - otherwise, set to [**prndefault**]
- If record is not ‘use as required’
  - use mean of closest of duration2, duration3, & duration4 if the difference between them is <[**maxdifference**]
  - otherwise, use duration1
  - otherwise, use median duration for that product for that person
  - otherwise, use [**default**]
- Create start = prescription date, stop = start + duration

**Part 3) Account for overlapping prescriptions and short gaps**

- Where records have overlapping start/stop exposure windows, truncate the earlier record. Add any truncated time to the next available gap, with maximum time carried-forward set to [**overlap**]
- Where there is a gap between records of less than [**maxgap**], extend the stop date of the earlier record to fill in the gap

# Additional Figure S1.1. Defining maximum prescription count.

We defined ‘prescription count’ as the number of medicines with overlapping prescription windows on any particular day. To calculate maximum prescription count before or after a review, we kept prescription records with start dates within the three months before the review (excluding the review date) and three months after the review (including the review date). We calculated the maximum number of overlapping prescriptions in these two windows, as demonstrated in the figure below.

**Additional Figure S1.1 Example: timing of prescriptions issued before and after a medication review.**

The horizontal blocks show when the person had active prescriptions for each medicine. The dotted vertical line shows the date of the medication review.


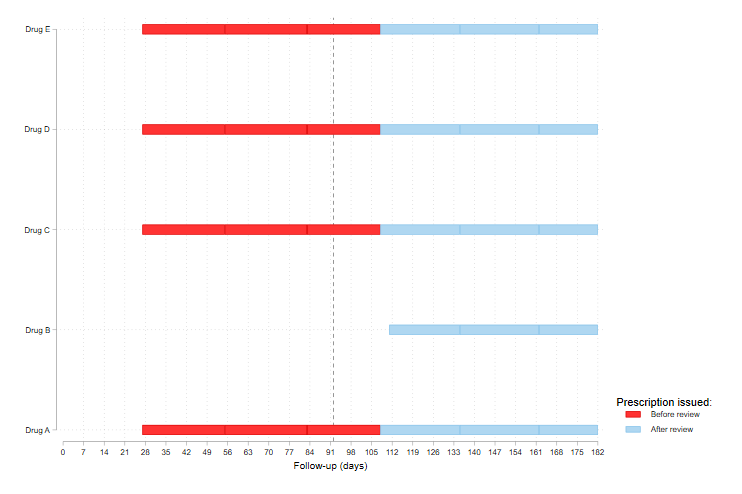


Max prescription count before review = 4

Max prescription count after review = 5

# Additional Table S1.1. Medication review Read codes.

^a^Only the flagged codes were included in the sensitivity analysis.

| **Read code** | **Description** | **Category^a^** |
| --- | --- | --- |
| 8B31400 | medication review | 1 |
| 8B3S.00 | medication review | 1 |
| 8B3x.00 | medication review with patient | 1 |
| 8B3V.00 | medication review done | 1 |
| 8BIC.00 | medication review done by pharmacist | 1 |
| 8B3y.00 | medication review of medical notes | 1 |
| 8BIy.00 | medication review done by nurse | 1 |
| 8BIH.00 | medication review done by doctor | 1 |
| 8BMH.00 | medication review done by pharmacy technician | 1 |
| 8BT..00 | medication review - additional | 1 |
| 8BMY.00 | medication review done by medicines management pharmacist | 1 |
| 8BMX.00 | medication review done by medicines management technician | 1 |
| 8BT2.00 | medication review by practice nurse | 1 |
| 8B31B00 | polypharmacy medication review | 1 |
| 8BIF.00 | epilepsy medication review |  |
| 8B3j.00 | asthma medication review |  |
| 8BI..00 | other medication review |  |
| 8B3l.00 | diabetes medication review |  |
| 8B3k.00 | coronary heart disease medication review |  |
| 8BM0.00 | mental health medication review |  |
| 9H91.00 | depression medication review |  |
| 8B3h.00 | medication review without patient |  |
| 8BT0.00 | concordance and compliance level 2 medication review |  |
| 8BM0100 | antipsychotic medication review |  |
| 8BT3.00 | anticoagulant medication review |  |
| 8BT4.00 | bisphosphonate medication review |  |
| 8BM0200 | dementia medication review |  |

# Additional Table S1.2. Staff role groupings.

| **code** | **description** | **category** |  | **code** | **description** | **category** |
| --- | --- | --- | --- | --- | --- | --- |
| 20 | Practice Manager | admin |  | 16 | Social Worker | other |
| 21 | Fund Manager | admin |  | 18 | Dispenser | other |
| 22 | Business Manager | admin |  | 19 | Non-qualified Dispenser | other |
| 23 | Administrator | admin |  | 26 | Physiotherapist | other |
| 24 | Secretary | admin |  | 27 | Chiropodist | other |
| 25 | Receptionist | admin |  | 28 | Dentist | other |
| 59 | Other Admin & Clerical | admin |  | 29 | Dietician | other |
| 1 | Senior Partner | GP |  | 30 | Counsellor | other |
| 2 | Partner | GP |  | 31 | Osteopath | other |
| 3 | Assistant | GP |  | 32 | Maintenance staff | other |
| 4 | Associate | GP |  | 33 | Other Health Care Professional | other |
| 5 | Non-commercial local rota of less than 10 GPs | GP |  | 35 | Community Medical Officer | other |
| 6 | Commercial Deputising service | GP |  | 37 | Health Education Officer | other |
| 7 | Locum | GP |  | 39 | Stomatherapist | other |
| 8 | GP Registrar | GP |  | 40 | Computer Manager | other |
| 9 | Consultant | GP |  | 41 | Interpreter/Link Worker | other |
| 10 | Sole Practitioner | GP |  | 42 | Chiropractor | other |
| 47 | Salaried Partner | GP |  | 43 | Acupuncturist | other |
| 50 | GP Retainer | GP |  | 44 | Homeopath | other |
| 60 | Clinical Practitioner Access Role | GP |  | 46 | Carer | other |
| 11 | Practice Nurse | nurse |  | 48 | Occupational Therapist | other |
| 12 | Health Visitor | nurse |  | 49 | Speech Therapist | other |
| 13 | Community Nurse | nurse |  | 51 | Phlebotomist | other |
| 14 | Midwife | nurse |  | 52 | Other Medical & Dental | other |
| 15 | Community Psychiatric Nurse | nurse |  | 53 | Other Students | other |
| 34 | Hospital Nurse | nurse |  | 55 | Other Allied Health Professionals | other |
| 36 | School Nurse | nurse |  | 56 | Other Professional Scientific & Technical | other |
| 38 | Contact Tracing Nurse | nurse |  | 57 | Other Healthcare Scientists | other |
| 45 | Mental Handicap Nurse | nurse |  | 58 | Other Additional Clinical Services | other |
| 54 | Other Nursing & Midwifery | nurse |  | 63 | Health Professional Access Role | other |
| 61 | Nurse Access Role | nurse |  | 64 | Healthcare Student Access Role | other |
| 62 | Nurse Manager Access Role | nurse |  | 65 | Biomedical Scientist Access Role | other |
| 17 | Pharmacist | pharmacist |  | 66 | Clinical Coder Access Role | other |
| 0 | Data Not Entered | unknown |  | 67 | Optometrist | other |
|  |  |  |  | 68 | Radiographer | other |

# Additional Table S1.3. Consultation type groupings.

| **Code** | **Description** | **Type** |  | **Code** | **Description** | **Type** |
| --- | --- | --- | --- | --- | --- | --- |
| 1 | Clinic | face-to-face |  | 5 | Mail from patient | other |
| 2 | Night visit, Deputising service | face-to-face |  | 12 | Discharge details | other |
| 3 | Follow-up/routine visit | face-to-face |  | 13 | Letter from Outpatients | other |
| 4 | Night visit, Local rota | face-to-face |  | 14 | Repeat Issue | other |
| 6 | Night visit , practice | face-to-face |  | 15 | Other | other |
| 7 | Out of hours, Practice | face-to-face |  | 16 | Results recording | other |
| 8 | Out of hours, Non Practice | face-to-face |  | 17 | Mail to patient | other |
| 9 | Surgery consultation | face-to-face |  | 18 | Emergency Consultation | other |
| 11 | Acute visit | face-to-face |  | 19 | Administration | other |
| 24 | Children's Home Visit | face-to-face |  | 20 | Casualty Attendance | other |
| 27 | Home Visit | face-to-face |  | 22 | Third Party Consultation | other |
| 28 | Hotel Visit | face-to-face |  | 23 | Hospital Admission | other |
| 30 | Nursing Home Visit | face-to-face |  | 25 | Day Case Report | other |
| 31 | Residential Home Visit | face-to-face |  | 26 | GOS18 Report | other |
| 32 | Twilight Visit | face-to-face |  | 29 | NHS Direct Report | other |
| 34 | Walk-in Centre | face-to-face |  | 33 | Triage | other |
| 36 | Co-op Surgery Consultation | face-to-face |  | 38 | Minor Injury Service | other |
| 37 | Co-op Home Visit | face-to-face |  | 39 | Medicine Management | other |
| 40 | Community Clinic | face-to-face |  | 41 | Community Nursing Note | other |
| 50 | Night Visit | face-to-face |  | 42 | Community Nursing Report | other |
| 10 | Telephone call from a patient | telephone |  | 43 | Data Transferred from other system | other |
| 21 | Telephone call to a patient | telephone |  | 44 | Health Authority Entry | other |
| 35 | Co-op Telephone advice | telephone |  | 45 | Health Visitor Note | other |
| 55 | Telephone Consultation | telephone |  | 46 | Health Visitor Report | other |
| 0 | Data Not Entered | unknown |  | 47 | Hospital Inpatient Report | other |
|  |  |  |  | 48 | Initial Post Discharge Review | other |
|  |  |  |  | 49 | Laboratory Request | other |
|  |  |  |  | 51 | Radiology Request | other |
|  |  |  |  | 52 | Radiology Result | other |
|  |  |  |  | 53 | Referral Letter | other |
|  |  |  |  | 54 | Social Services Report | other |
|  |  |  |  | 56 | Template Entry | other |
|  |  |  |  | 57 | GP to GP communication transaction | other |
|  |  |  |  | 58 | Non-consultation medication data | other |
|  |  |  |  | 59 | Non-consultation data | other |
|  |  |  |  | 60 | ePharmacy message | other |
|  |  |  |  | 61 | Extended Hours | other |

# Additional Methods S3. Definitions of other variables

For all variables (except ethnicity), observations recorded before the practice up-to-standard date were discarded. Unless otherwise stated, indicator variables were defined as ever/never being recorded on or before 01 Jan 2019. Records for diagnoses and related variables were defined using pre-specified code lists which were sourced from existing resources or developed for this study. Items marked * were modified/extended/developed by the team for this study.

- **Age** in 2019. 2019 minus year of birth.
- **Sex**. Male/Female. Person dropped if missing or not classified.
- **Ethnicity**(1)*. Asian or Asian British, Black or Black British, Mixed, White, Chinese or other ethnic group. Where multiple ethnicities were recorded, the most recently recorded was kept. Unlike other variables, data recorded before the up-to-standard date were used (to try to reduce the amount of missing data).
- **Deprivation**. Townsend quintile. Patient postcode-level if available, otherwise practice postcode-level.
- **Practice region**. Defined by CPRD, map to Office for National Statistics regions. Collapsed to Scotland, Wales, Northern Ireland, London, Rest of England in some analyses as the dataset had small numbers of practices in some regions of England.
- **Smoking status**(2). Never, former, current, missing. Uses Read coded information, additional clinical information (amount smoked), and prescriptions for smoking cessation therapy. Most recent status on/before cohort entry.
- **Alcohol use**(3). Non-, former, occasional, moderate, heavy drinker. Based on existing Read code list. Most recent status on/before cohort entry.
- **Body mass index**. Underweight (<18.5 kg/m^2^), healthy (18.5-24.9 kg/m^2^), overweight (25-29.9 kg/m^2^), obese class I (30-34.5 kg/m^2^), obese class II (35-39.9 kg/m^2^), obese class III+ (>=40 kg/m^2^). Calculated from cleaned height and weight values recorded as additional clinical information. Most recent weight on/before cohort entry divided by (median height)^2^.
- **Read-coded diagnoses/health factors**. Considered present if there was a record on or before cohort entry date. Full list:
  - Living in a care home(4, 5)*, atrial fibrillation(6), cancer(7), chronic kidney disease(8), chronic obstructive pulmonary disease(6), coronary heart disease (including angina and myocardial infarction)(8), dementia(6), depression(9-11), anxiety(9), diabetes(6), epilepsy(6), heart failure(6), hypertension(6), hypothyroidism(8), learning disabilities(6), mental health disorder (schizophrenia, bipolar affective disorder, other psychoses)(6, 12), obesity(6), osteoporosis(6), being on the palliative care pathway(13), peripheral arterial disease(12, 14), rheumatoid arthritis(12), stroke or TIA(6), asthma(6), dyslipidaemia (Read code for dyslipidaemia or test result showing high total cholesterol, LDL-cholesterol, or triglycerides)(15), gout(6), glaucoma(6), Parkinson’s disease(6), benign prostatic hyperplasia(6), problems with urinary control (incontinence or retention)(6, 16)*, mobility problems(5), increased risk of blood clots (thrombosis, thrombophilia)(6, 17), severe frailty including recent falls and fractures (Read code or frailty score ‘severe frailty’ or fall/fracture Read code in year prior to baseline)(18).
- **Prescribed medicines according to British National Formulary chapter**: indicators for chapters 1-13 and chapter unknown, according to having an active prescription on 01 Jan 2019.
- **Use of specific medicines:** yes/no based on having a prescription on or in the six months before cohort entry date (definitions in Table S1.1 below)

**Additional Table S1.4. Terms used to define specified drug groups.**

| Variable | Definition |
| --- | --- |
| NSAIDs | BNF 10.01.01 (Non-steroidal anti-inflammatory drugs) |
| Oral anticoagulants | BNF 02.08.02 (Oral anticoagulants)  *exclude result drug name ‘pentosan’*  *Note ‘phenindione’ not present in dataset* |
| Aspirin/antiplatelet medicines | BNF 02.09.** (Antiplatelet drugs)  *or*  Drug name “aspirin” |
| Renin-angiotensin system drugs  includes combination renin-angiotensin + diuretic (+ calcium channel blocker) | BNF 02.05.05 (Drugs affecting the renin-angiotensin system) |
| Diuretics | BNF 02.02.** (Diuretics, incs subsections) |
| Opioids  Includes combination opioid + non-opioid analgesics | BNF 04.07.02 (Opioid analgesics)  or  Drug name “dihydrocodeine” or “codeine” or “dextropropoxyphene” |
| Benzodiazepines and Z-drugs | Drug name "alprazolam" or "chlordiazepoxide" or "clobazam" or "clonazepam" or "diazepam" or "flurazepam" or "loprazolam" or "lorazepam" or "lormetazepam" or "midazolam" or "nitrazepam" or "temazepam"  *or*  Drug name “zolpidem” or “zopiclone” |
| Gabapentinoids | Drug name “pregabalin” or “gabapentin” |
| Inhaled long-acting beta-agonists and corticosteroids | BNF 03.02.** (Corticosteroids)  *or*  Drug name “formoterol” or “eformoterol” or “salmeterol” or “indacaterol” or “olodaterol” or “bambuterol” or “vilanterol” |
| Antidepressants | BNF 4.3.** (Antidepressant drugs) |
| Antipsychotics | BNF 4.2.1 (Antipsychotic drugs)  *or*  BNF 4.2.2 (Antipsychotic depot injections)  *exclude result drug name ‘prochlorperazine’* |
| Lithium | Drug name “lithium” |
| Bisphosphonates | BNF 06.06.02 (Bisphosphonates and other dugs affecting bone metabolism) |
| Anticholinergic medicines  <https://www.pharmdlive.com/blog/anticholinergic-medications-in-the-beers-criteria/>  2019 Beer’s Criteria  <https://agsjournals.onlinelibrary.wiley.com/doi/10.1111/jgs.15767>  plus Coupland 2019  <https://doi.org/10.1001/jamainternmed.2019.0677> | **Drug names (2019 Beer’s criteria):**  “disopyramide” or “amitriptyline” or “amoxapine” or “clomipramine” or “desipramine” or “doxepin” or “imipramine” or “nortriptyline” or “paroxetine” or “protriptyline” or “trimipramine” or “prochlorperazine” or “promethazine” or “pyrilamine” or “triprolidine” or “brompheniramine” or “carbinoxamine” or “chlorpheniramine” or “clemastine” or “cyproheptadine” or “dexbrompheniramine” or “dexchlorpheniramine” or “dimenhydrinate” or “diphenhydramine” (*tablets only*) or “doxylamine” or “hydroxyzine” or “meclizine” or “clidinium-chlordiazepoxide” or “dicyclomine” or “homatropine” or “hyoscyamine” or “methscopolamine” or “propantheline” or “darifenacin” or “fesoterodine” or “flavoxate” or “oxybutynin” or “solifenacin” or “tolterodine” or “trospium” or “benztropine” or “trihexyphenidyl” or “chlorpromazine” or “clozapine” or “loxapine” or “olanzapine” or “perphenazine” or “thioridazine” or “trifluoperazine” or “atropine” (*excl. drops*) or “belladonna” or “scopolamine” or “cyclobenzaprine” or “orphenadrine”  **Drug names (Coupland 2019):**  “dosulepin” or “lofepramine” or “cyclizine” or “dimenhydrinate” or “promazine” or “azatadine” or “chlorphenamine” or “trimeprazine” or “alimemazine” or “propiverine” or “benzatropine” or “orphenadrine” or “methotrimeprazine” or “levomepromazine” or “pericyazine” or “perphenazine” or “pimozide” or “quetiapine” or “alverine” or “dicyclomine” or “dicycloverine” or “propantheline” or “hyoscine” or “carbamazepine” or “oxcarbazepine” or “methocarbamol” or “tizanidine” or “glycopyrrolate” or “glycopyrronium” or “ipratropium” |

# References

1. Wright AK, Kontopantelis E, Emsley R, Buchan I, Sattar N, Rutter MK, et al. Life Expectancy and Cause-Specific Mortality in Type 2 Diabetes: A Population-Based Cohort Study Quantifying Relationships in Ethnic Subgroups. Diabetes Care. 2017;40(3):338-45. doi:10.2337/dc16-1616.

2. Joseph RM, Movahedi M, Dixon WG, Symmons DP. Smoking-Related Mortality in Patients With Early Rheumatoid Arthritis: A Retrospective Cohort Study Using the Clinical Practice Research Datalink. Arthritis Care Res (Hoboken). 2016;68(11):1598-606. doi:10.1002/acr.22882.

3. Bell S, Daskalopoulou M, Rapsomaniki E, George J, Britton A, Bobak M, et al. Association between clinically recorded alcohol consumption and initial presentation of 12 cardiovascular diseases: population based cohort study using linked health records. BMJ. 2017;356:j909. doi:10.1136/bmj.j909.

4. Schultze A, Bates C, Cockburn J, MacKenna B, Nightingale E, Curtis HJ, et al. Identifying Care Home Residents in Electronic Health Records - An OpenSAFELY Short Data Report. Wellcome Open Res. 2021;6:90. doi:10.12688/wellcomeopenres.16737.1.

5. Joseph RM, Jack RH, Morriss R, Knaggs RD, Butler D, Hollis C, et al. The risk of all-cause and cause-specific mortality in people prescribed mirtazapine: an active comparator cohort study using electronic health records. BMC Med. 2022;20(1):43. doi:10.1186/s12916-022-02247-x.

6. Denaxas S, Gonzalez-Izquierdo A, Direk K, Fitzpatrick NK, Fatemifar G, Banerjee A, et al. UK phenomics platform for developing and validating electronic health record phenotypes: CALIBER. J Am Med Inform Assoc. 2019;26(12):1545-59. doi:10.1093/jamia/ocz105.

7. Khan NF, Perera R, Harper S, Rose PW. Adaptation and validation of the Charlson Index for Read/OXMIS coded databases. BMC Fam Pract. 2010;11:1. doi:10.1186/1471-2296-11-1.

8. Zghebi SS, Reeves D, Grigoroglou C, McMillan B, Ashcroft DM, Parisi R, et al. Clinical code usage in UK general practice: a cohort study exploring 18 conditions over 14 years. BMJ Open. 2022;12(7):e051456. doi:10.1136/bmjopen-2021-051456.

9. John A, McGregor J, Fone D, Dunstan F, Cornish R, Lyons RA, et al. Case-finding for common mental disorders of anxiety and depression in primary care: an external validation of routinely collected data. BMC Med Inform Decis Mak. 2016;16:35. doi:10.1186/s12911-016-0274-7.

10. Martinez C, Rietbrock S, Wise L, Ashby D, Chick J, Moseley J, et al. Antidepressant treatment and the risk of fatal and non-fatal self harm in first episode depression: nested case-control study. BMJ. 2005;330(7488):389. doi:10.1136/bmj.330.7488.389.

11. Carreira H, Williams R, Strongman H, Bhaskaran K. Identification of mental health and quality of life outcomes in primary care databases in the UK: a systematic review. BMJ Open. 2019;9(7):e029227. doi:10.1136/bmjopen-2019-029227.

12. University of Oxford for the Bennett Institute for Applied Data Science. OpenCodelists. 2022. <https://www.opencodelists.org/>. Accessed 14 Mar 2023.

13. NHS Digital. Quality and Outcomes Framework (QOF) business rules v 38 2017-2018 October code release. 2017. <https://webarchive.nationalarchives.gov.uk/ukgwa/20220117164934/https://digital.nhs.uk/data-and-information/data-collections-and-data-sets/data-collections/quality-and-outcomes-framework-qof/quality-and-outcome-framework-qof-business-rules/quality-and-outcomes-framework-qof-business-rules-v-38-2017-2018-october-code-release>. Accessed 14 Mar 2023.

14. Iwagami M, Caplin B, Smeeth L, Tomlinson LA, Nitsch D. Chronic kidney disease and cause-specific hospitalisation: a matched cohort study using primary and secondary care patient data. Br J Gen Pract. 2018;68(673):e512-e23. doi:10.3399/bjgp18X697973.

15. Weng S, Kai J, Akyea R, Qureshi N. Detection of familial hypercholesterolaemia: external validation of the FAMCAT clinical case-finding algorithm to identify patients in primary care. Lancet Public Health. 2019;4(5):e256-e64. doi:10.1016/S2468-2667(19)30061-1.

16. Grant RL, Drennan VM, Rait G, Petersen I, Iliffe S. First diagnosis and management of incontinence in older people with and without dementia in primary care: a cohort study using The Health Improvement Network primary care database. PLoS Med. 2013;10(8):e1001505. doi:10.1371/journal.pmed.1001505.

17. Stocks SJ, Kontopantelis E, Akbarov A, Rodgers S, Avery AJ, Ashcroft DM. Examining variations in prescribing safety in UK general practice: cross sectional study using the Clinical Practice Research Datalink. BMJ. 2015;351:h5501. doi:10.1136/bmj.h5501.

18. NHS Digital. Quality and Outcomes Framework (QOF) business rules v47.0 2022-2023 baseline release. 2022. <https://digital.nhs.uk/data-and-information/data-collections-and-data-sets/data-collections/quality-and-outcomes-framework-qof#quality-and-outcome-framework-business-rules>. Accessed 14 Mar 2023.
