# Supplementary material for: Frequency and impact of medication reviews for people aged 65 years or above in UK primary care: an observational study using electronic health records
Source: BMC Geriatr. 2023 Jul 14;23:435. doi: 10.1186/s12877-023-04143-2 (PMC10347807; doi:10.1186/s12877-023-04143-2)

Additional File 2

Additional results.

Contents

[Supplementary Table S2.1. Full baseline characteristics of the study population as of 01 January 2019. 2](#_Toc129613404)

[Supplementary Table S2.2. Full results, association between baseline factors and having a medication review in 2019, Cox regression. 4](#_Toc129613405)

[Supplementary Table S2.3. Sensitivity analysis: association between baseline factors and having a medication review in 2019, Cox regression using conservative definition of medication reviews. 7](#_Toc129613406)

[Supplementary Table S2.4. Characteristics and mean change in prescription count for people with a medication review and at least three months of subsequent follow-up. 10](#_Toc129613407)

[Supplementary Table S2.5. Twenty most frequently prescribed medicines only before and only after a medication review. 12](#_Toc129613408)

[Supplementary Figure S2.1. Distribution of change (difference) in prescription count before (A) and after (B) dropping extreme values. 14](#_Toc129613409)

[Supplementary Table S2.6. Cross-tabulation of maximum prescription count before vs after a medication review. 15](#_Toc129613410)

[Supplementary Figure S2.2. Bar charts showing the mean change in prescription count by demographic and other characteristics. 16](#_Toc129613411)

# Additional Table S2.1. Full baseline characteristics of the study population as of 01 January 2019.

Results for people aged 65 years or older with at least one ongoing prescription on 01 January 2019. COPD chronic obstructive pulmonary disease, TIA transient ischaemic attack, NSAIDs non-steroidal anti-inflammatory drugs

|  | **Medication review in 2019** | |  |
| --- | --- | --- | --- |
|  | **No** | **Yes** | **Whole study population** |
| **Overall count (number of people)** | 286,200 | 305,526 | 591,726 |
| **Uncensored follow-up (person-years)** | 259,764 | 296,570 | 556,334 |
| **Age group (years) in 2019** |  |  |  |
| 65-74 | 149,168 (52.1%) | 147,918 (48.4%) | 297,086 (50.2%) |
| 75-84 | 96,114 (33.6%) | 110,534 (36.2%) | 206,648 (34.9%) |
| 85-94 | 36,970 (12.9%) | 43,127 (14.1%) | 80,097 (13.5%) |
| 95+ | 3,948 (1.4%) | 3,947 (1.3%) | 7,895 (1.3%) |
| **Sex** |  |  |  |
| Male | 130,550 (45.6%) | 138,847 (45.4%) | 269,397 (45.5%) |
| Female | 155,650 (54.4%) | 166,679 (54.6%) | 322,329 (54.5%) |
| **Ethnicity** |  |  |  |
| Asian/British Asian | 2,201 (0.8%) | 2,184 (0.7%) | 4,385 (0.7%) |
| Black/Black British | 569 (0.2%) | 484 (0.2%) | 1,053 (0.2%) |
| Mixed | 282 (0.1%) | 229 (0.1%) | 511 (0.1%) |
| Other | 1,072 (0.4%) | 1,033 (0.3%) | 2,105 (0.4%) |
| White | 143,017 (50.0%) | 140,015 (45.8%) | 283,032 (47.8%) |
| Missing | 139,059 (48.6%) | 161,581 (52.9%) | 300,640 (50.8%) |
| **Townsend Score quintile** |  |  |  |
| Quintile 1 (least deprived) | 42,823 (15.0%) | 44,588 (14.6%) | 87,411 (14.8%) |
| Quintile 2 | 50,671 (17.7%) | 52,117 (17.1%) | 102,788 (17.4%) |
| Quintile 3 | 80,077 (28.0%) | 87,105 (28.5%) | 167,182 (28.3%) |
| Quintile 4 | 74,446 (26.0%) | 79,073 (25.9%) | 153,519 (25.9%) |
| Quintile 5 (most deprived) | 38,183 (13.3%) | 42,643 (14.0%) | 80,826 (13.7%) |
| **Practice region** |  |  |  |
| North West | 15,561 (5.4%) | 11,113 (3.6%) | 26,674 (4.5%) |
| Yorkshire & The Humber | 620 (0.2%) | 485 (0.2%) | 1,105 (0.2%) |
| West Midlands | 13,049 (4.6%) | 12,803 (4.2%) | 25,852 (4.4%) |
| East of England | 2,202 (0.8%) | 1,268 (0.4%) | 3,470 (0.6%) |
| South East | 12,984 (4.5%) | 6,523 (2.1%) | 19,507 (3.3%) |
| South West | 38,146 (13.3%) | 20,250 (6.6%) | 58,396 (9.9%) |
| London | 4,369 (1.5%) | 3,956 (1.3%) | 8,325 (1.4%) |
| Scotland | 74,115 (25.9%) | 94,792 (31.0%) | 168,907 (28.5%) |
| Wales | 101,940 (35.6%) | 132,131 (43.2%) | 234,071 (39.6%) |
| Northern Ireland | 23,214 (8.1%) | 22,205 (7.3%) | 45,419 (7.7%) |
| **Smoking status** |  |  |  |
| Never | 110,988 (38.8%) | 111,752 (36.6%) | 222,740 (37.6%) |
| Former | 141,763 (49.5%) | 159,640 (52.3%) | 301,403 (50.9%) |
| Current | 29,303 (10.2%) | 30,734 (10.1%) | 60,037 (10.1%) |
| Missing | 4,146 (1.4%) | 3,400 (1.1%) | 7,546 (1.3%) |
| **Alcohol intake** |  |  |  |
| Non-drinker | 39,585 (13.8%) | 42,445 (13.9%) | 82,030 (13.9%) |
| Former drinker | 34,139 (11.9%) | 44,345 (14.5%) | 78,484 (13.3%) |
| Occasional | 75,375 (26.3%) | 85,986 (28.1%) | 161,361 (27.3%) |
| Moderate | 31,026 (10.8%) | 36,694 (12.0%) | 67,720 (11.4%) |
| Heavy | 6,679 (2.3%) | 6,929 (2.3%) | 13,608 (2.3%) |
| Missing | 99,396 (34.7%) | 89,127 (29.2%) | 188,523 (31.9%) |
| **BMI category** |  |  |  |
| Underweight (<18.5 kg/m^2^) | 5,048 (1.8%) | 5,129 (1.7%) | 10,177 (1.7%) |
| Healthy (18.5-24.9 kg/m^2^) | 73,228 (25.6%) | 73,408 (24.0%) | 146,636 (24.8%) |
| Overweight (25-29.9 kg/m^2^) | 96,445 (33.7%) | 103,519 (33.9%) | 199,964 (33.8%) |
| Obese class 1 (30-34.5 kg/m^2^) | 48,384 (16.9%) | 57,380 (18.8%) | 105,764 (17.9%) |
| Obese class 2 (35-39.9 kg/m^2^) | 16,079 (5.6%) | 19,953 (6.5%) | 36,032 (6.1%) |
| Obese class 3+ (40+ kg/m^2^) | 6,825 (2.4%) | 8,837 (2.9%) | 15,662 (2.6%) |
| Missing | 40,191 (14.0%) | 37,300 (12.2%) | 77,491 (13.1%) |
| **Number of ongoing prescriptions at baseline** |  |  |  |
| 1 | 42,825 (15.0%) | 24,454 (8.0%) | 67,279 (11.4%) |
| 2-4 | 114,980 (40.2%) | 106,088 (34.7%) | 221,068 (37.4%) |
| 5-9 | 101,209 (35.4%) | 130,820 (42.8%) | 232,029 (39.2%) |
| 10-14 | 23,437 (8.2%) | 37,335 (12.2%) | 60,772 (10.3%) |
| 15-19 | 3,338 (1.2%) | 6,028 (2.0%) | 9,366 (1.6%) |
| 20+ | 411 (0.1%) | 801 (0.3%) | 1,212 (0.2%) |
| **Medication review in previous year** | 120,457 (42.1%) | 207,071 (67.8%) | 327,528 (55.4%) |
| **Medication review in previous year (conservative definition)** | 61,295 (21.4%) | 100,657 (32.9%) | 161,952 (27.4%) |
| **Living in a care home** | 3,892 (1.4%) | 7,569 (2.5%) | 11,461 (1.9%) |
| **Diagnoses / clinical indicators** |  |  |  |
| Atrial fibrillation | 25,388 (8.9%) | 36,836 (12.1%) | 62,224 (10.5%) |
| Cancer | 57,405 (20.1%) | 62,107 (20.3%) | 119,512 (20.2%) |
| Chronic kidney disease | 42,616 (14.9%) | 52,546 (17.2%) | 95,162 (16.1%) |
| COPD | 23,862 (8.3%) | 30,908 (10.1%) | 54,770 (9.3%) |
| Coronary heart disease | 33,533 (11.7%) | 44,986 (14.7%) | 78,519 (13.3%) |
| Dementia | 11,972 (4.2%) | 14,401 (4.7%) | 26,373 (4.5%) |
| Depression | 43,848 (15.3%) | 53,489 (17.5%) | 97,337 (16.4%) |
| Anxiety | 25,782 (9.0%) | 30,558 (10.0%) | 56,340 (9.5%) |
| Diabetes | 40,091 (14.0%) | 54,350 (17.8%) | 94,441 (16.0%) |
| Epilepsy | 4,183 (1.5%) | 5,447 (1.8%) | 9,630 (1.6%) |
| Heart failure | 11,846 (4.1%) | 16,882 (5.5%) | 28,728 (4.9%) |
| Hypertension | 126,155 (44.1%) | 149,230 (48.8%) | 275,385 (46.5%) |
| Hypothyroidism | 24,460 (8.5%) | 28,207 (9.2%) | 52,667 (8.9%) |
| Learning disability | 765 (0.3%) | 903 (0.3%) | 1,668 (0.3%) |
| Schizophrenia, bipolar affective disorder, or other psychosis | 2,182 (0.8%) | 2,957 (1.0%) | 5,139 (0.9%) |
| Obesity | 27,509 (9.6%) | 35,331 (11.6%) | 62,840 (10.6%) |
| Osteoporosis | 28,066 (9.8%) | 33,260 (10.9%) | 61,326 (10.4%) |
| On palliative care pathway | 3,704 (1.3%) | 3,596 (1.2%) | 7,300 (1.2%) |
| Peripheral arterial disease | 8,111 (2.8%) | 10,908 (3.6%) | 19,019 (3.2%) |
| Rheumatoid arthritis | 4,283 (1.5%) | 6,059 (2.0%) | 10,342 (1.7%) |
| Stroke or TIA | 25,325 (8.8%) | 33,080 (10.8%) | 58,405 (9.9%) |
| Asthma | 27,104 (9.5%) | 35,234 (11.5%) | 62,338 (10.5%) |
| Dyslipidaemia | 157,673 (55.1%) | 172,685 (56.5%) | 330,358 (55.8%) |
| Gout | 20,422 (7.1%) | 24,428 (8.0%) | 44,850 (7.6%) |
| Glaucoma | 12,303 (4.3%) | 14,076 (4.6%) | 26,379 (4.5%) |
| Parkinson’s disease | 2,433 (0.9%) | 3,274 (1.1%) | 5,707 (1.0%) |
| Benign prostatic hyperplasia | 19,274 (6.7%) | 22,754 (7.4%) | 42,028 (7.1%) |
| Urinary incontinence or retention | 25,319 (8.8%) | 31,736 (10.4%) | 57,055 (9.6%) |
| Mobility problems | 77,784 (27.2%) | 97,175 (31.8%) | 174,959 (29.6%) |
| Thrombosis or thrombophilia | 41,491 (14.5%) | 53,279 (17.4%) | 94,770 (16.0%) |
| Severe frailty, recent fall, recent fracture | 13,094 (4.6%) | 16,278 (5.3%) | 29,372 (5.0%) |
| **Medicines prescribed in the 6 months prior to baseline** |  |  |  |
| NSAIDs | 21,485 (7.5%) | 23,446 (7.7%) | 44,931 (7.6%) |
| Oral anticoagulants | 26,574 (9.3%) | 39,351 (12.9%) | 65,925 (11.1%) |
| Aspirin/antiplatelet medicines | 67,694 (23.7%) | 84,330 (27.6%) | 152,024 (25.7%) |
| Renin-angiotensin system drugs | 108,410 (37.9%) | 133,328 (43.6%) | 241,738 (40.9%) |
| Diuretics | 64,065 (22.4%) | 82,012 (26.8%) | 146,077 (24.7%) |
| Opioids (including combination painkillers) | 65,708 (23.0%) | 83,406 (27.3%) | 149,114 (25.2%) |
| Antidepressants | 53,880 (18.8%) | 71,215 (23.3%) | 125,095 (21.1%) |
| Antipsychotics | 4,803 (1.7%) | 6,622 (2.2%) | 11,425 (1.9%) |
| Bisphosphonates | 16,805 (5.9%) | 20,572 (6.7%) | 37,377 (6.3%) |
| Benzodiazepines and Z-drugs | 20,085 (7.0%) | 26,171 (8.6%) | 46,256 (7.8%) |
| Gabapentinoids | 12,944 (4.5%) | 19,134 (6.3%) | 32,078 (5.4%) |
| Inhaled long-acting beta-antagonists and corticosteroids | 36,131 (12.6%) | 47,782 (15.6%) | 83,913 (14.2%) |
| Lithium | 470 (0.2%) | 697 (0.2%) | 1,167 (0.2%) |
| Anticholinergic medicines | 49,935 (17.4%) | 64,667 (21.2%) | 114,602 (19.4%) |
| **Active prescriptions at baseline, BNF Chapter-level** |  |  |  |
| 1 (Gastro-intestinal system) | 110,385 (38.6%) | 138,613 (45.4%) | 248,998 (42.1%) |
| 2 (Cardiovascular system) | 207,770 (72.6%) | 244,021 (79.9%) | 451,791 (76.4%) |
| 3 (Respiratory system) | 50,812 (17.8%) | 65,141 (21.3%) | 115,953 (19.6%) |
| 4 (Central nervous system) | 104,828 (36.6%) | 133,617 (43.7%) | 238,445 (40.3%) |
| 5 (Infections) | 16,430 (5.7%) | 21,201 (6.9%) | 37,631 (6.4%) |
| 6 (Endocrine system) | 86,905 (30.4%) | 109,017 (35.7%) | 195,922 (33.1%) |
| 7 (Obstetrics, gynaecology, and urinary-tract disorders) | 36,863 (12.9%) | 44,402 (14.5%) | 81,265 (13.7%) |
| 8 (Malignant disease and immunosuppression) | 7,280 (2.5%) | 8,466 (2.8%) | 15,746 (2.7%) |
| 9 (Nutrition and blood) | 56,665 (19.8%) | 69,625 (22.8%) | 126,290 (21.3%) |
| 10 (Musculoskeletal and joint disease) | 35,272 (12.3%) | 44,858 (14.7%) | 80,130 (13.5%) |
| 11 (Eye) | 34,632 (12.1%) | 40,734 (13.3%) | 75,366 (12.7%) |
| 12 (Ear, nose, and oropharynx) | 17,553 (6.1%) | 20,360 (6.7%) | 37,913 (6.4%) |
| 13 (Skin) | 37,143 (13.0%) | 45,033 (14.7%) | 82,176 (13.9%) |
| Unspecified | 4,241 (1.5%) | 5,078 (1.7%) | 9,319 (1.6%) |

# Additional Table S2.2. Full results, association between baseline factors and having a medication review in 2019, Cox regression.

The adjusted models (columns 4-8) are adjusted for the factors with results shown in each column. COPD chronic obstructive pulmonary disease, TIA transient ischaemic attack, NSAIDs non-steroidal anti-inflammatory drugs, BNF British National Formulary

|  | **Hazard ratios (95% confidence intervals), p-value** | | | | | | |
| --- | --- | --- | --- | --- | --- | --- | --- |
|  | Unadjusted | Age-sex adjusted | Adjusted model 1 | Adjusted model 2 | Adjusted model 3 | Adjusted model 4 | Adjusted model final |
| **Age (vs 65, per 10-year increase)** | 1.12 (1.10, 1.13), p<0.001 | .. | .. | .. | .. | .. | .. |
| **Age group, years (vs 65-74)** |  |  |  |  |  |  |  |
| 75-84 | 1.13 (1.11, 1.15), p<0.001 | 1.13 (1.11, 1.15), p<0.001 | 1.05 (1.03, 1.06), p<0.001 | 1.03 (1.02, 1.04), p<0.001 | 1.04 (1.03, 1.06), p<0.001 | 1.04 (1.03, 1.06), p<0.001 | 1.03 (1.02, 1.05), p<0.001 |
| 85-94 | 1.22 (1.19, 1.26), p<0.001 | 1.22 (1.19, 1.26), p<0.001 | 1.07 (1.05, 1.09), p<0.001 | 1.03 (1.00, 1.05), p=0.037 | 1.06 (1.04, 1.09), p<0.001 | 1.07 (1.04, 1.09), p<0.001 | 1.04 (1.01, 1.06), p=0.006 |
| 95+ | 1.26 (1.20, 1.32), p<0.001 | 1.26 (1.20, 1.33), p<0.001 | 1.04 (0.99, 1.08), p=0.088 | 1.00 (0.95, 1.04), p=0.869 | 1.05 (1.00, 1.10), p=0.034 | 1.04 (1.00, 1.09), p=0.081 | 1.02 (0.97, 1.07), p=0.516 |
| **Sex (female vs male)** | 1.01 (0.99, 1.02), p=0.315 | 0.99 (0.98, 1.01), p=0.304 | 1.00 (0.99, 1.01), p=0.999 | 1.00 (0.99, 1.01), p=0.494 | 1.00 (0.99, 1.01), p=0.718 | 0.99 (0.98, 1.00), p=0.116 | 1.00 (0.99, 1.01), p=0.781 |
| **Townsend quintile (vs 1, least deprived)** |  |  |  |  |  |  |  |
| Quintile 2 | 0.99 (0.81, 1.21), p=0.935 | 0.99 (0.81, 1.22), p=0.938 | 0.95 (0.81, 1.11), p=0.496 | 0.94 (0.81, 1.11), p=0.481 | 0.95 (0.81, 1.11), p=0.490 | 0.94 (0.80, 1.11), p=0.480 | 0.94 (0.80, 1.11), p=0.477 |
| Quintile 3 | 1.03 (0.85, 1.25), p=0.756 | 1.03 (0.85, 1.25), p=0.754 | 0.97 (0.84, 1.12), p=0.685 | 0.97 (0.84, 1.12), p=0.701 | 0.97 (0.84, 1.12), p=0.653 | 0.97 (0.84, 1.12), p=0.644 | 0.97 (0.84, 1.12), p=0.678 |
| Quintile 4 | 1.02 (0.82, 1.26), p=0.891 | 1.02 (0.82, 1.26), p=0.870 | 0.96 (0.81, 1.13), p=0.604 | 0.96 (0.81, 1.13), p=0.612 | 0.95 (0.81, 1.13), p=0.579 | 0.95 (0.81, 1.12), p=0.567 | 0.96 (0.81, 1.13), p=0.594 |
| Quintile 5 | 1.04 (0.84, 1.30), p=0.700 | 1.05 (0.84, 1.30), p=0.669 | 0.98 (0.83, 1.16), p=0.842 | 0.98 (0.83, 1.16), p=0.786 | 0.98 (0.83, 1.16), p=0.818 | 0.98 (0.83, 1.16), p=0.794 | 0.98 (0.82, 1.15), p=0.776 |
| **Practice region (vs Scotland)** |  |  |  |  |  |  |  |
| Wales | 1.01 (0.87, 1.17), p=0.904 | 1.01 (0.87, 1.17), p=0.942 | 1.00 (0.88, 1.13), p=0.986 | 0.99 (0.87, 1.13), p=0.938 | 1.00 (0.88, 1.13), p=0.949 | 1.00 (0.88, 1.13), p=0.965 | 0.99 (0.87, 1.13), p=0.913 |
| Northern Ireland | 0.82 (0.64, 1.06), p=0.131 | 0.82 (0.64, 1.06), p=0.128 | 0.83 (0.67, 1.03), p=0.085 | 0.82 (0.66, 1.02), p=0.068 | 0.82 (0.66, 1.02), p=0.078 | 0.82 (0.66, 1.02), p=0.079 | 0.81 (0.65, 1.01), p=0.064 |
| London | 0.54 (0.39, 0.74), p<0.001 | 0.53 (0.38, 0.74), p<0.001 | 0.62 (0.47, 0.82), p=0.001 | 0.62 (0.47, 0.81), p=0.001 | 0.62 (0.47, 0.81), p=0.001 | 0.62 (0.47, 0.81), p=0.001 | 0.62 (0.47, 0.81), p=0.001 |
| Rest of England | 0.72 (0.58, 0.88), p=0.001 | 0.71 (0.58, 0.87), p=0.001 | 0.79 (0.66, 0.93), p=0.006 | 0.77 (0.65, 0.91), p=0.003 | 0.78 (0.66, 0.93), p=0.005 | 0.78 (0.66, 0.93), p=0.005 | 0.77 (0.65, 0.91), p=0.003 |
| **Number of ongoing prescriptions at baseline (vs 1)** |  |  |  |  |  |  |  |
| 2-4 | 1.45 (1.42, 1.49), p<0.001 | 1.44 (1.41, 1.48), p<0.001 | 1.32 (1.29, 1.36), p<0.001 | 1.29 (1.25, 1.32), p<0.001 | 1.29 (1.26, 1.32), p<0.001 | 1.25 (1.22, 1.28), p<0.001 | 1.27 (1.24, 1.30), p<0.001 |
| 5-9 | 1.88 (1.81, 1.95), p<0.001 | 1.84 (1.77, 1.92), p<0.001 | 1.58 (1.53, 1.64), p<0.001 | 1.47 (1.41, 1.52), p<0.001 | 1.47 (1.43, 1.52), p<0.001 | 1.40 (1.36, 1.45), p<0.001 | 1.41 (1.37, 1.46), p<0.001 |
| 10-14 | 2.25 (2.14, 2.37), p<0.001 | 2.20 (2.09, 2.32), p<0.001 | 1.81 (1.73, 1.90), p<0.001 | 1.59 (1.52, 1.67), p<0.001 | 1.60 (1.54, 1.66), p<0.001 | 1.52 (1.46, 1.58), p<0.001 | 1.48 (1.43, 1.55), p<0.001 |
| 15-19 | 2.51 (2.35, 2.67), p<0.001 | 2.46 (2.31, 2.63), p<0.001 | 1.98 (1.87, 2.09), p<0.001 | 1.67 (1.57, 1.77), p<0.001 | 1.67 (1.59, 1.75), p<0.001 | 1.61 (1.52, 1.69), p<0.001 | 1.51 (1.44, 1.59), p<0.001 |
| 20+ | 2.83 (2.56, 3.13), p<0.001 | 2.81 (2.54, 3.11), p<0.001 | 2.27 (2.07, 2.50), p<0.001 | 1.86 (1.69, 2.05), p<0.001 | 1.84 (1.68, 2.02), p<0.001 | 1.81 (1.65, 1.99), p<0.001 | 1.64 (1.49, 1.80), p<0.001 |
| **Medication review in previous year** | 2.05 (1.89, 2.21), p<0.001 | 2.03 (1.88, 2.20), p<0.001 | 1.85 (1.71, 2.01), p<0.001 | 1.83 (1.69, 1.99), p<0.001 | 1.84 (1.70, 1.99), p<0.001 | 1.84 (1.70, 2.00), p<0.001 | 1.83 (1.69, 1.98), p<0.001 |
| **Living in a care home** | 1.98 (1.83, 2.15), p<0.001 | 1.87 (1.73, 2.03), p<0.001 | 1.53 (1.42, 1.64), p<0.001 | 1.51 (1.40, 1.62), p<0.001 | 1.53 (1.42, 1.64), p<0.001 | 1.53 (1.42, 1.65), p<0.001 | 1.51 (1.40, 1.62), p<0.001 |
| **Diagnoses/clinical indicators** |  |  |  |  |  |  |  |
| Atrial fibrillation | 1.33 (1.29, 1.37), p<0.001 | 1.29 (1.25, 1.33), p<0.001 | .. | 1.14 (1.11, 1.16), p<0.001 | .. | .. | 1.05 (1.03, 1.08), p<0.001 |
| Cancer | 1.05 (1.03, 1.07), p<0.001 | 1.02 (1.00, 1.04), p=0.017 | .. | 1.01 (0.99, 1.02), p=0.297 | .. | .. | 1.01 (0.99, 1.02), p=0.235 |
| Chronic kidney disease | 1.19 (1.15, 1.23), p<0.001 | 1.14 (1.10, 1.18), p<0.001 | .. | 1.03 (1.01, 1.06), p=0.015 | .. | .. | 1.03 (1.01, 1.06), p=0.018 |
| COPD | 1.19 (1.16, 1.23), p<0.001 | 1.19 (1.16, 1.23), p<0.001 | .. | 1.03 (1.01, 1.06), p=0.003 | .. | .. | 1.02 (1.00, 1.05), p=0.082 |
| Coronary heart disease | 1.23 (1.20, 1.26), p<0.001 | 1.21 (1.18, 1.24), p<0.001 | .. | 1.03 (1.01, 1.05), p=0.010 | .. | .. | 1.04 (1.02, 1.06), p<0.001 |
| Dementia | 1.29 (1.19, 1.39), p<0.001 | 1.21 (1.12, 1.30), p<0.001 | .. | 1.02 (0.98, 1.06), p=0.324 | .. | .. | 1.02 (0.98, 1.06), p=0.322 |
| Depression | 1.13 (1.10, 1.17), p<0.001 | 1.16 (1.12, 1.20), p<0.001 | .. | 1.05 (1.03, 1.08), p<0.001 | .. | .. | 1.03 (1.01, 1.06), p=0.009 |
| Anxiety | 1.11 (1.05, 1.17), p<0.001 | 1.12 (1.06, 1.18), p<0.001 | .. | 1.04 (1.00, 1.08), p=0.057 | .. | .. | 1.03 (0.99, 1.07), p=0.145 |
| Diabetes | 1.24 (1.20, 1.28), p<0.001 | 1.24 (1.20, 1.28), p<0.001 | .. | 1.08 (1.05, 1.11), p<0.001 | .. | .. | 1.08 (1.06, 1.11), p<0.001 |
| Epilepsy | 1.18 (1.15, 1.22), p<0.001 | 1.19 (1.15, 1.23), p<0.001 | .. | 1.06 (1.03, 1.09), p<0.001 | .. | .. | 1.06 (1.03, 1.10), p<0.001 |
| Heart failure | 1.32 (1.29, 1.37), p<0.001 | 1.28 (1.24, 1.32), p<0.001 | .. | 1.02 (1.00, 1.05), p=0.086 | .. | .. | 1.01 (0.99, 1.04), p=0.297 |
| Hypertension | 1.15 (1.11, 1.18), p<0.001 | 1.13 (1.10, 1.17), p<0.001 | .. | 1.06 (1.03, 1.08), p<0.001 | .. | .. | 1.05 (1.03, 1.08), p<0.001 |
| Hypothyroidism | 1.08 (1.05, 1.10), p<0.001 | 1.07 (1.04, 1.10), p<0.001 | .. | 1.02 (1.00, 1.04), p=0.109 | .. | .. | 1.02 (1.00, 1.05), p=0.036 |
| Learning disability | 1.12 (1.02, 1.23), p=0.014 | 1.16 (1.06, 1.27), p=0.002 | .. | 0.96 (0.88, 1.05), p=0.385 | .. | .. | 0.96 (0.88, 1.05), p=0.373 |
| Schizophrenia, bipolar affective disorder, or other psychosis | 1.23 (1.17, 1.29), p<0.001 | 1.24 (1.19, 1.30), p<0.001 |  | 1.06 (1.02, 1.11), p=0.003 |  |  | 1.03 (0.99, 1.07), p=0.200 |
| Obesity | 1.13 (1.06, 1.21), p<0.001 | 1.15 (1.08, 1.22), p<0.001 | .. | 0.99 (0.95, 1.04), p=0.798 | .. | .. | 0.99 (0.94, 1.04), p=0.695 |
| Osteoporosis | 1.11 (1.08, 1.14), p<0.001 | 1.08 (1.05, 1.11), p<0.001 | .. | 1.01 (0.99, 1.03), p=0.408 | .. | .. | 1.01 (0.99, 1.04), p=0.213 |
| On palliative care pathway | 1.34 (1.24, 1.44), p<0.001 | 1.27 (1.18, 1.37), p<0.001 | .. | 1.02 (0.97, 1.09), p=0.421 | .. | .. | 1.02 (0.96, 1.08), p=0.551 |
| Peripheral arterial disease | 1.22 (1.18, 1.26), p<0.001 | 1.20 (1.16, 1.24), p<0.001 | .. | 1.03 (1.01, 1.06), p=0.011 | .. | .. | 1.03 (1.01, 1.06), p=0.015 |
| Rheumatoid arthritis | 1.25 (1.19, 1.30), p<0.001 | 1.25 (1.20, 1.31), p<0.001 | .. | 1.12 (1.08, 1.17), p<0.001 | .. | .. | 1.13 (1.09, 1.17), p<0.001 |
| Stroke or TIA | 1.21 (1.19, 1.24), p<0.001 | 1.18 (1.16, 1.20), p<0.001 | .. | 1.02 (1.00, 1.04), p=0.035 | .. | .. | 1.03 (1.01, 1.05), p=0.001 |
| Asthma | 1.17 (1.14, 1.21), p<0.001 | 1.18 (1.14, 1.22), p<0.001 | .. | 1.06 (1.04, 1.09), p<0.001 | .. | .. | 1.05 (1.02, 1.07), p=0.001 |
| Dyslipidaemia | 1.04 (1.00, 1.07), p=0.023 | 1.04 (1.01, 1.08), p=0.007 | .. | 0.99 (0.97, 1.01), p=0.523 | .. | .. | 0.99 (0.97, 1.01), p=0.527 |
| Gout | 1.11 (1.08, 1.14), p<0.001 | 1.10 (1.07, 1.13), p<0.001 | .. | 1.01 (0.99, 1.03), p=0.416 | .. | .. | 1.01 (0.99, 1.03), p=0.425 |
| Glaucoma | 1.08 (1.05, 1.11), p<0.001 | 1.04 (1.01, 1.07), p=0.004 | .. | 1.02 (1.00, 1.05), p=0.083 | .. | .. | 1.03 (1.01, 1.05), p=0.017 |
| Parkinson’s disease | 1.27 (1.22, 1.33), p<0.001 | 1.24 (1.19, 1.29), p<0.001 | .. | 1.12 (1.07, 1.16), p<0.001 | .. | .. | 1.12 (1.08, 1.17), p<0.001 |
| Benign prostatic hyperplasia | 1.09 (1.06, 1.13), p<0.001 | 1.08 (1.04, 1.12), p<0.001 | .. | 1.04 (1.01, 1.07), p=0.010 | .. | .. | 1.04 (1.01, 1.07), p=0.005 |
| Urinary incontinence or retention | 1.17 (1.13, 1.22), p<0.001 | 1.16 (1.12, 1.20), p<0.001 | .. | 1.07 (1.04, 1.09), p<0.001 | .. | .. | 1.06 (1.04, 1.09), p<0.001 |
| Mobility problems | 1.25 (1.20, 1.30), p<0.001 | 1.21 (1.16, 1.27), p<0.001 | .. | 1.02 (0.98, 1.06), p=0.298 | .. | .. | 1.01 (0.98, 1.05), p=0.511 |
| Thrombosis or thrombophilia | 1.21 (1.18, 1.23), p<0.001 | 1.18 (1.16, 1.20), p<0.001 | .. | 1.01 (0.99, 1.03), p=0.422 | .. | .. | 0.99 (0.97, 1.01), p=0.387 |
| Severe frailty, recent fall, recent fracture | 1.22 (1.18, 1.26), p<0.001 | 1.18 (1.14, 1.21), p<0.001 | .. | 1.06 (1.04, 1.09), p<0.001 | .. | .. | 1.06 (1.03, 1.08), p<0.001 |
| **Medicines prescribed in the 6 months prior to baseline** |  |  |  |  |  |  |  |
| NSAIDs | 1.00 (0.97, 1.02), p=0.811 | 1.02 (1.00, 1.05), p=0.072 | .. | .. | 1.00 (0.98, 1.02), p=0.997 | .. | 1.01 (0.98, 1.03), p=0.482 |
| Oral anticoagulants | 1.34 (1.30, 1.38), p<0.001 | 1.30 (1.26, 1.34), p<0.001 | .. | .. | 1.16 (1.13, 1.20), p<0.001 | .. | 1.12 (1.08, 1.15), p<0.001 |
| Aspirin/antiplatelet medicines | 1.17 (1.15, 1.19), p<0.001 | 1.15 (1.13, 1.17), p<0.001 | .. | .. | 1.01 (0.99, 1.02), p=0.204 | .. | 1.00 (0.98, 1.01), p=0.618 |
| Renin-angiotensin system drugs | 1.17 (1.15, 1.19), p<0.001 | 1.17 (1.15, 1.19), p<0.001 | .. | .. | 1.05 (1.03, 1.06), p<0.001 | .. | 1.03 (1.01, 1.05), p<0.001 |
| Diuretics | 1.21 (1.19, 1.23), p<0.001 | 1.18 (1.16, 1.20), p<0.001 | .. | .. | 1.01 (0.99, 1.02), p=0.338 | .. | 1.00 (0.99, 1.02), p=0.807 |
| Opioids (including combination painkillers) | 1.20 (1.17, 1.23), p<0.001 | 1.20 (1.17, 1.22), p<0.001 | .. | .. | 1.01 (1.00, 1.03), p=0.080 | .. | 1.01 (1.00, 1.03), p=0.099 |
| Antidepressants | 1.24 (1.21, 1.27), p<0.001 | 1.25 (1.23, 1.28), p<0.001 | .. | .. | 1.06 (1.05, 1.08), p<0.001 | .. | 1.05 (1.03, 1.06), p<0.001 |
| Antipsychotics | 1.39 (1.33, 1.45), p<0.001 | 1.38 (1.32, 1.44), p<0.001 | .. | .. | 1.06 (1.03, 1.10), p<0.001 | .. | 1.05 (1.02, 1.09), p=0.004 |
| Bisphosphonates | 1.13 (1.11, 1.15), p<0.001 | 1.10 (1.08, 1.13), p<0.001 | .. | .. | 1.00 (0.98, 1.02), p=0.950 | .. | 1.00 (0.98, 1.02), p=0.899 |
| Benzodiazepines and Z-drugs | 1.22 (1.18, 1.26), p<0.001 | 1.20 (1.17, 1.24), p<0.001 | .. | .. | 1.02 (1.00, 1.05), p=0.026 | .. | 1.02 (1.00, 1.04), p=0.045 |
| Gabapentinoids | 1.31 (1.28, 1.35), p<0.001 | 1.32 (1.28, 1.36), p<0.001 | .. | .. | 1.11 (1.08, 1.13), p<0.001 | .. | 1.10 (1.08, 1.13), p<0.001 |
| Inhaled long-acting beta-antagonists and corticosteroids | 1.20 (1.17, 1.23), p<0.001 | 1.21 (1.18, 1.24), p<0.001 | .. | .. | 1.06 (1.04, 1.08), p<0.001 | .. | 1.04 (1.02, 1.06), p<0.001 |
| Lithium | 1.28 (1.19, 1.39), p<0.001 | 1.30 (1.20, 1.41), p<0.001 | .. | .. | 1.07 (0.99, 1.15), p=0.086 | .. | 1.06 (0.98, 1.14), p=0.178 |
| Anticholinergic medicines | 1.21 (1.18, 1.23), p<0.001 | 1.21 (1.18, 1.23), p<0.001 | .. | .. | 1.01 (1.00, 1.03), p=0.036 | .. | 1.01 (1.00, 1.03), p=0.036 |
| Active prescriptions at baseline, BNF Chapter-level |  |  |  |  |  |  |  |
| 1 (Gastro-intestinal system) | 1.25 (1.22, 1.27), p<0.001 | 1.23 (1.21, 1.25), p<0.001 | .. | .. | .. | 1.03 (1.02, 1.04), p<0.001 | .. |
| 2 (Cardiovascular system) | 1.34 (1.31, 1.37), p<0.001 | 1.32 (1.28, 1.35), p<0.001 | .. | .. | .. | 1.11 (1.09, 1.13), p<0.001 | .. |
| 3 (Respiratory system) | 1.19 (1.16, 1.22), p<0.001 | 1.19 (1.17, 1.22), p<0.001 | .. | .. | .. | 1.05 (1.03, 1.06), p<0.001 | .. |
| 4 (Central nervous system) | 1.28 (1.25, 1.31), p<0.001 | 1.27 (1.24, 1.30), p<0.001 | .. | .. | .. | 1.07 (1.05, 1.08), p<0.001 | .. |
| 5 (Infections) | 1.20 (1.17, 1.23), p<0.001 | 1.19 (1.16, 1.22), p<0.001 | .. | .. | .. | 1.03 (1.01, 1.05), p=0.001 | .. |
| 6 (Endocrine system) | 1.21 (1.19, 1.23), p<0.001 | 1.19 (1.18, 1.21), p<0.001 | .. | .. | .. | 1.05 (1.04, 1.06), p<0.001 | .. |
| 7 (Obstetrics, gynaecology, and urinary-tract disorders) | 1.10 (1.08, 1.11), p<0.001 | 1.10 (1.08, 1.11), p<0.001 | .. | .. | .. | 1.00 (0.99, 1.01), p=0.873 | .. |
| 8 (Malignant disease and immunosuppression) | 1.10 (1.07, 1.12), p<0.001 | 1.09 (1.06, 1.11), p<0.001 | .. | .. | .. | 1.03 (1.00, 1.05), p=0.039 | .. |
| 9 (Nutrition and blood) | 1.19 (1.16, 1.22), p<0.001 | 1.16 (1.13, 1.19), p<0.001 | .. | .. | .. | 1.00 (0.98, 1.02), p=0.868 | .. |
| 10 (Musculoskeletal and joint disease) | 1.16 (1.13, 1.18), p<0.001 | 1.15 (1.13, 1.17), p<0.001 | .. | .. | .. | 1.02 (1.00, 1.03), p=0.019 | .. |
| 11 (Eye) | 1.09 (1.07, 1.11), p<0.001 | 1.06 (1.05, 1.08), p<0.001 | .. | .. | .. | 0.99 (0.98, 1.00), p=0.132 | .. |
| 12 (Ear, nose, and oropharynx) | 1.06 (1.04, 1.09), p<0.001 | 1.07 (1.04, 1.09), p<0.001 | .. | .. | .. | 0.97 (0.95, 0.99), p=0.005 | .. |
| 13 (Skin) | 1.14 (1.12, 1.17), p<0.001 | 1.12 (1.10, 1.15), p<0.001 | .. | .. | .. | 1.00 (0.98, 1.01), p=0.540 | .. |
| Unspecified | 1.10 (1.05, 1.16), p<0.001 | 1.09 (1.04, 1.15), p<0.001 | .. | .. | .. | 0.98 (0.94, 1.02), p=0.374 | .. |

# Additional Table S2.3. Sensitivity analysis: association between baseline factors and having a medication review in 2019, Cox regression using conservative definition of medication reviews.

The Adjusted model (column 4) is adjusted for the factors with results shown. N number, COPD chronic obstructive pulmonary disease, TIA transient ischaemic attack, NSAIDs non-steroidal anti-inflammatory drugs, BNF British National Formulary.

|  | **Hazard ratios (95% confidence intervals), p-value** | | |
| --- | --- | --- | --- |
|  | Unadjusted | Age-sex adjusted | Adjusted model |
| **Age (vs 65, per 10-year increase)** | 1.07 (1.05, 1.09), p<0.001 | .. | .. |
| **Age group, years (vs 65-74)** |  |  |  |
| 75-84 | 1.11 (1.09, 1.13), p<0.001 | 1.11 (1.09, 1.13), p<0.001 | 1.04 (1.02, 1.06), p<0.001 |
| 85-94 | 1.12 (1.08, 1.16), p<0.001 | 1.12 (1.08, 1.16), p<0.001 | 1.02 (0.99, 1.05), p=0.300 |
| 95+ | 1.08 (1.00, 1.16), p=0.054 | 1.08 (1.00, 1.16), p=0.052 | 0.95 (0.89, 1.01), p=0.106 |
| **Sex (female vs male)** | 1.01 (0.99, 1.02), p=0.435 | 1.00 (0.98, 1.02), p=0.983 | 1.01 (0.99, 1.02), p=0.476 |
| **Townsend quintile (vs 1, least deprived)** |  |  |  |
| Quintile 2 | 1.06 (0.82, 1.38), p=0.636 | 1.06 (0.82, 1.38), p=0.634 | 1.06 (0.87, 1.29), p=0.546 |
| Quintile 3 | 0.95 (0.75, 1.22), p=0.704 | 0.95 (0.75, 1.22), p=0.703 | 0.95 (0.79, 1.14), p=0.570 |
| Quintile 4 | 0.84 (0.65, 1.09), p=0.187 | 0.84 (0.65, 1.09), p=0.191 | 0.89 (0.73, 1.09), p=0.261 |
| Quintile 5 | 0.97 (0.72, 1.29), p=0.818 | 0.97 (0.72, 1.30), p=0.831 | 0.99 (0.80, 1.24), p=0.961 |
| **Practice region (vs Scotland)** |  |  |  |
| Wales | 1.41 (1.17, 1.70), p<0.001 | 1.40 (1.16, 1.70), p<0.001 | 1.29 (1.10, 1.50), p=0.001 |
| Northern Ireland | 0.64 (0.45, 0.91), p=0.014 | 0.64 (0.45, 0.91), p=0.014 | 0.66 (0.49, 0.90), p=0.008 |
| London | 1.03 (0.68, 1.55), p=0.891 | 1.03 (0.68, 1.55), p=0.899 | 1.05 (0.74, 1.49), p=0.776 |
| Rest of England | 1.25 (0.97, 1.60), p=0.085 | 1.24 (0.97, 1.60), p=0.087 | 1.18 (0.96, 1.44), p=0.113 |
| **Number of ongoing prescriptions at baseline (vs 1)** |  |  |  |
| 2-4 | 1.39 (1.35, 1.43), p<0.001 | 1.38 (1.34, 1.42), p<0.001 | 1.24 (1.21, 1.27), p<0.001 |
| 5-9 | 1.71 (1.63, 1.79), p<0.001 | 1.69 (1.61, 1.77), p<0.001 | 1.36 (1.31, 1.42), p<0.001 |
| 10-14 | 1.91 (1.79, 2.03), p<0.001 | 1.89 (1.77, 2.01), p<0.001 | 1.40 (1.33, 1.46), p<0.001 |
| 15-19 | 2.06 (1.90, 2.22), p<0.001 | 2.04 (1.88, 2.21), p<0.001 | 1.41 (1.32, 1.51), p<0.001 |
| 20+ | 2.09 (1.84, 2.38), p<0.001 | 2.08 (1.83, 2.37), p<0.001 | 1.41 (1.25, 1.59), p<0.001 |
| **Medication review in previous year** | 2.68 (2.47, 2.90), p<0.001 | 2.67 (2.46, 2.90), p<0.001 | 2.46 (2.27, 2.67), p<0.001 |
| **Living in a care home** | 1.54 (1.36, 1.74), p<0.001 | 1.51 (1.34, 1.70), p<0.001 | 1.42 (1.28, 1.56), p<0.001 |
| **Diagnoses/clinical indicators** |  |  |  |
| Atrial fibrillation | 1.18 (1.14, 1.22), p<0.001 | 1.15 (1.11, 1.20), p<0.001 | 1.04 (1.00, 1.07), p=0.027 |
| Cancer | 1.04 (1.02, 1.07), p=0.001 | 1.03 (1.00, 1.05), p=0.032 | 1.00 (0.98, 1.02), p=0.787 |
| Chronic kidney disease | 1.13 (1.08, 1.19), p<0.001 | 1.11 (1.05, 1.16), p<0.001 | 1.02 (0.98, 1.05), p=0.332 |
| COPD | 1.18 (1.13, 1.23), p<0.001 | 1.17 (1.12, 1.22), p<0.001 | 1.09 (1.05, 1.13), p<0.001 |
| Coronary heart disease | 1.15 (1.11, 1.19), p<0.001 | 1.14 (1.10, 1.18), p<0.001 | 1.03 (1.00, 1.06), p=0.062 |
| Dementia | 1.13 (1.03, 1.23), p=0.009 | 1.08 (0.99, 1.18), p=0.074 | 0.97 (0.91, 1.03), p=0.307 |
| Depression | 1.18 (1.13, 1.23), p<0.001 | 1.20 (1.15, 1.25), p<0.001 | 1.06 (1.03, 1.09), p<0.001 |
| Anxiety | 1.17 (1.11, 1.24), p<0.001 | 1.18 (1.12, 1.25), p<0.001 | 1.04 (1.00, 1.08), p=0.075 |
| Diabetes | 1.23 (1.16, 1.29), p<0.001 | 1.22 (1.16, 1.29), p<0.001 | 1.08 (1.03, 1.13), p=0.001 |
| Epilepsy | 1.05 (1.00, 1.10), p=0.040 | 1.05 (1.01, 1.11), p=0.027 | 0.99 (0.95, 1.03), p=0.688 |
| Heart failure | 1.21 (1.16, 1.26), p<0.001 | 1.19 (1.14, 1.24), p<0.001 | 1.01 (0.97, 1.05), p=0.678 |
| Hypertension | 1.18 (1.13, 1.23), p<0.001 | 1.17 (1.12, 1.22), p<0.001 | 1.07 (1.04, 1.11), p<0.001 |
| Hypothyroidism | 1.05 (1.01, 1.08), p=0.007 | 1.04 (1.01, 1.08), p=0.015 | 1.01 (0.98, 1.04), p=0.541 |
| Learning disability | 1.07 (0.94, 1.21), p=0.318 | 1.09 (0.96, 1.24), p=0.190 | 1.01 (0.90, 1.14), p=0.827 |
| Schizophrenia, bipolar affective disorder, or other psychosis | 1.10 (1.03, 1.17), p=0.004 | 1.11 (1.04, 1.18), p=0.002 | 0.97 (0.91, 1.03), p=0.280 |
| Obesity | 1.06 (0.98, 1.14), p=0.171 | 1.06 (0.98, 1.15), p=0.118 | 1.01 (0.95, 1.07), p=0.865 |
| Osteoporosis | 1.06 (1.02, 1.09), p=0.001 | 1.04 (1.01, 1.07), p=0.020 | 1.03 (1.00, 1.06), p=0.037 |
| On palliative care pathway | 1.20 (1.07, 1.34), p=0.002 | 1.17 (1.05, 1.31), p=0.005 | 0.99 (0.90, 1.09), p=0.853 |
| Peripheral arterial disease | 1.11 (1.07, 1.16), p<0.001 | 1.10 (1.06, 1.15), p<0.001 | 1.02 (0.99, 1.05), p=0.237 |
| Rheumatoid arthritis | 1.12 (1.07, 1.17), p<0.001 | 1.12 (1.07, 1.17), p<0.001 | 1.04 (1.00, 1.08), p=0.067 |
| Stroke or TIA | 1.13 (1.10, 1.16), p<0.001 | 1.11 (1.08, 1.14), p<0.001 | 1.03 (1.00, 1.06), p=0.055 |
| Asthma | 1.10 (1.06, 1.14), p<0.001 | 1.11 (1.07, 1.15), p<0.001 | 1.02 (0.99, 1.05), p=0.267 |
| Dyslipidaemia | 1.06 (1.01, 1.10), p=0.007 | 1.06 (1.02, 1.10), p=0.004 | 0.98 (0.96, 1.01), p=0.167 |
| Gout | 1.14 (1.10, 1.18), p<0.001 | 1.13 (1.09, 1.17), p<0.001 | 1.02 (1.00, 1.05), p=0.089 |
| Glaucoma | 1.04 (1.00, 1.08), p=0.026 | 1.02 (0.99, 1.06), p=0.263 | 0.99 (0.96, 1.02), p=0.341 |
| Parkinson’s disease | 1.16 (1.09, 1.24), p<0.001 | 1.14 (1.08, 1.21), p<0.001 | 1.07 (1.01, 1.14), p=0.014 |
| Benign prostatic hyperplasia | 1.09 (1.04, 1.14), p<0.001 | 1.08 (1.03, 1.14), p=0.002 | 1.04 (1.00, 1.08), p=0.035 |
| Urinary incontinence or retention | 1.16 (1.11, 1.21), p<0.001 | 1.15 (1.10, 1.20), p<0.001 | 1.04 (1.01, 1.07), p=0.018 |
| Mobility problems | 1.07 (1.02, 1.12), p=0.009 | 1.04 (0.99, 1.10), p=0.138 | 0.96 (0.93, 1.00), p=0.069 |
| Thrombosis or thrombophilia | 1.13 (1.10, 1.15), p<0.001 | 1.11 (1.08, 1.14), p<0.001 | 0.99 (0.97, 1.02), p=0.700 |
| Severe frailty, recent fall, recent fracture | 1.18 (1.13, 1.23), p<0.001 | 1.16 (1.11, 1.21), p<0.001 | 1.02 (0.99, 1.06), p=0.168 |
| **Medicines prescribed in the 6 months prior to baseline** |  |  |  |
| NSAIDs | 1.05 (1.01, 1.09), p=0.026 | 1.07 (1.02, 1.11), p=0.002 | 1.02 (0.99, 1.06), p=0.222 |
| Oral anticoagulants | 1.18 (1.14, 1.22), p<0.001 | 1.16 (1.11, 1.20), p<0.001 | 1.02 (0.98, 1.06), p=0.396 |
| Aspirin/antiplatelet medicines | 1.11 (1.08, 1.14), p<0.001 | 1.09 (1.07, 1.12), p<0.001 | 0.99 (0.97, 1.01), p=0.298 |
| Renin-angiotensin system drugs | 1.18 (1.16, 1.21), p<0.001 | 1.18 (1.15, 1.21), p<0.001 | 1.03 (1.01, 1.05), p=0.004 |
| Diuretics | 1.15 (1.12, 1.17), p<0.001 | 1.13 (1.11, 1.16), p<0.001 | 1.00 (0.98, 1.02), p=0.909 |
| Opioids (including combination painkillers) | 1.16 (1.12, 1.20), p<0.001 | 1.16 (1.12, 1.20), p<0.001 | 1.02 (1.00, 1.05), p=0.033 |
| Antidepressants | 1.25 (1.21, 1.29), p<0.001 | 1.26 (1.22, 1.30), p<0.001 | 1.08 (1.05, 1.11), p<0.001 |
| Antipsychotics | 1.17 (1.10, 1.25), p<0.001 | 1.17 (1.10, 1.24), p<0.001 | 1.01 (0.96, 1.06), p=0.818 |
| Bisphosphonates | 1.07 (1.04, 1.10), p<0.001 | 1.05 (1.02, 1.08), p=0.001 | 0.98 (0.95, 1.01), p=0.144 |
| Benzodiazepines and Z-drugs | 1.15 (1.09, 1.20), p<0.001 | 1.14 (1.09, 1.19), p<0.001 | 1.04 (1.01, 1.07), p=0.016 |
| Gabapentinoids | 1.26 (1.22, 1.31), p<0.001 | 1.27 (1.22, 1.31), p<0.001 | 1.07 (1.04, 1.11), p<0.001 |
| Inhaled long-acting beta-antagonists and corticosteroids | 1.11 (1.07, 1.14), p<0.001 | 1.11 (1.07, 1.14), p<0.001 | 0.97 (0.94, 0.99), p=0.018 |
| Lithium | 1.15 (1.03, 1.28), p=0.011 | 1.16 (1.04, 1.30), p=0.007 | 1.07 (0.96, 1.18), p=0.227 |
| Anticholinergic medicines | 1.18 (1.15, 1.21), p<0.001 | 1.18 (1.15, 1.21), p<0.001 | 1.02 (1.00, 1.04), p=0.050 |
| **Active prescriptions at baseline, BNF Chapter-level** |  |  |  |
| 1 (Gastro-intestinal system) | 1.20 (1.17, 1.23), p<0.001 | 1.19 (1.16, 1.22), p<0.001 | .. |
| 2 (Cardiovascular system) | 1.31 (1.27, 1.36), p<0.001 | 1.30 (1.26, 1.34), p<0.001 | .. |
| 3 (Respiratory system) | 1.11 (1.08, 1.14), p<0.001 | 1.11 (1.08, 1.14), p<0.001 | .. |
| 4 (Central nervous system) | 1.23 (1.19, 1.27), p<0.001 | 1.23 (1.19, 1.27), p<0.001 | .. |
| 5 (Infections) | 1.17 (1.14, 1.21), p<0.001 | 1.16 (1.13, 1.20), p<0.001 | .. |
| 6 (Endocrine system) | 1.16 (1.13, 1.18), p<0.001 | 1.15 (1.12, 1.18), p<0.001 | .. |
| 7 (Obstetrics, gynaecology, and urinary-tract disorders) | 1.10 (1.08, 1.13), p<0.001 | 1.11 (1.08, 1.13), p<0.001 | .. |
| 8 (Malignant disease and immunosuppression) | 1.03 (0.99, 1.07), p=0.142 | 1.02 (0.99, 1.06), p=0.227 | .. |
| 9 (Nutrition and blood) | 1.13 (1.09, 1.16), p<0.001 | 1.11 (1.08, 1.15), p<0.001 | .. |
| 10 (Musculoskeletal and joint disease) | 1.13 (1.10, 1.16), p<0.001 | 1.13 (1.10, 1.16), p<0.001 | .. |
| 11 (Eye) | 1.08 (1.05, 1.10), p<0.001 | 1.06 (1.04, 1.09), p<0.001 | .. |
| 12 (Ear, nose, and oropharynx) | 1.07 (1.04, 1.11), p<0.001 | 1.08 (1.05, 1.11), p<0.001 | .. |
| 13 (Skin) | 1.08 (1.05, 1.11), p<0.001 | 1.07 (1.04, 1.10), p<0.001 | .. |
| Unspecified | 1.09 (1.03, 1.15), p=0.003 | 1.09 (1.03, 1.15), p=0.003 | .. |

# Additional Table S2.4. Characteristics and mean change in prescription count for people with a medication review and at least three months of subsequent follow-up.

*Results are shown before and after excluding people with no prescriptions in the three months before a review, and with a change in count more than three standard deviations from the mean. Change in prescription count is calculated as the maximum prescription count in the three months after a medication review minus the maximum prescription count in the three months before a medication review. SD standard deviation

|  | Before excluding people* | | After excluding people* | |
| --- | --- | --- | --- | --- |
|  | Count (%) | Mean (SD) change in prescription count | Count (%) | Mean (SD) change in prescription count |
| **Number** | 309,207 | 0.17 (1.41) | 291,947 | 0.13 (1.11) |
| **Age group (years)** |  |  |  |  |
| 65-74 | 153,143 (49.5%) | 0.19 (1.34) | 143,205 (49.1%) | 0.13 (1.06) |
| 75-84 | 110,754 (35.8%) | 0.17 (1.44) | 105,576 (36.2%) | 0.14 (1.13) |
| 85-94 | 41,743 (13.5%) | 0.13 (1.56) | 39,772 (13.6%) | 0.12 (1.21) |
| 95+ | 3,567 (1.2%) | 0.06 (1.59) | 3,394 (1.2%) | 0.06 (1.28) |
| **Sex** |  |  |  |  |
| Male | 140,851 (45.6%) | 0.19 (1.40) | 133,006 (45.6%) | 0.14 (1.08) |
| Female | 168,356 (54.4%) | 0.16 (1.42) | 158,941 (54.4%) | 0.13 (1.14) |
| **Maximum prescription count in the three months before the review** |  |  |  |  |
| 0 | 11,949 (3.9%) | 1.71 (2.13) | .. | .. |
| 1 | 23,627 (7.6%) | 0.48 (1.20) | 23,261 (8.0%) | 0.38 (0.88) |
| 2-4 | 103,697 (33.5%) | 0.26 (1.11) | 102,758 (35.2%) | 0.22 (0.97) |
| 5-9 | 128,460 (41.5%) | 0.06 (1.33) | 126,336 (43.3%) | 0.09 (1.14) |
| 10-14 | 35,441 (11.5%) | -0.24 (1.71) | 34,080 (11.7%) | -0.11 (1.35) |
| 15-19 | 5,424 (1.8%) | -0.65 (2.07) | 4,995 (1.7%) | -0.33 (1.46) |
| 20-24 | 609 (0.2%) | -1.16 (2.67) | 517 (0.2%) | -0.45 (1.61) |
| **Ethnicity** |  |  |  |  |
| Asian/British Asian | 2,140 (0.7%) | 0.28 (1.73) | 1,986 (0.7%) | 0.17 (1.17) |
| Black/Black British | 492 (0.2%) | 0.39 (2.08) | 425 (0.1%) | 0.16 (1.23) |
| Mixed | 242 (0.1%) | 0.71 (2.20) | 206 (0.1%) | 0.22 (1.18) |
| Other | 1,066 (0.3%) | 0.23 (1.62) | 974 (0.3%) | 0.12 (1.04) |
| White | 140,924 (45.6%) | 0.17 (1.42) | 133,228 (45.6%) | 0.13 (1.12) |
| Missing | 164,343 (53.1%) | 0.18 (1.40) | 155,128 (53.1%) | 0.13 (1.10) |
| **Townsend score quintile** |  |  |  |  |
| Quintile 1 (least deprived) | 45,331 (14.7%) | 0.18 (1.36) | 42,498 (14.6%) | 0.13 (1.10) |
| Quintile 2 | 52,740 (17.1%) | 0.19 (1.43) | 49,663 (17.0%) | 0.14 (1.12) |
| Quintile 3 | 88,012 (28.5%) | 0.18 (1.36) | 83,645 (28.7%) | 0.14 (1.08) |
| Quintile 4 | 79,942 (25.9%) | 0.16 (1.43) | 75,710 (25.9%) | 0.12 (1.12) |
| Quintile 5 (most deprived) | 43,182 (14.0%) | 0.17 (1.50) | 40,431 (13.8%) | 0.13 (1.14) |
| **CPRD practice region** |  |  |  |  |
| North West | 11,243 (3.6%) | 0.09 (1.42) | 10,756 (3.7%) | 0.08 (1.13) |
| Yorkshire & The Humber | 482 (0.2%) | 0.09 (1.90) | 447 (0.2%) | 0.00 (1.26) |
| West Midlands | 12,338 (4.0%) | 0.19 (1.36) | 11,818 (4.0%) | 0.15 (1.08) |
| East of England | 1,104 (0.4%) | 0.23 (1.56) | 1,009 (0.3%) | 0.17 (1.17) |
| London | 6,380 (2.1%) | 0.38 (1.71) | 5,692 (1.9%) | 0.20 (1.12) |
| South East | 18,875 (6.1%) | 0.21 (1.37) | 17,823 (6.1%) | 0.15 (1.10) |
| South West | 3,886 (1.3%) | 0.17 (1.47) | 3,667 (1.3%) | 0.11 (1.13) |
| Wales | 96,736 (31.3%) | 0.18 (1.31) | 92,952 (31.8%) | 0.14 (1.06) |
| Scotland | 135,663 (43.9%) | 0.15 (1.45) | 126,556 (43.3%) | 0.11 (1.13) |
| Northern Ireland | 22,500 (7.3%) | 0.23 (1.54) | 21,227 (7.3%) | 0.19 (1.19) |
| **CPRD practice region, country-level** |  |  |  |  |
| Scotland | 135,663 (43.9%) | 0.15 (1.45) | 126,556 (43.3%) | 0.11 (1.13) |
| Wales | 96,736 (31.3%) | 0.18 (1.31) | 92,952 (31.8%) | 0.14 (1.06) |
| Northern Ireland | 22,500 (7.3%) | 0.23 (1.54) | 21,227 (7.3%) | 0.19 (1.19) |
| London | 6,380 (2.1%) | 0.38 (1.71) | 5,692 (1.9%) | 0.20 (1.12) |
| Rest of England | 47,928 (15.5%) | 0.17 (1.40) | 45,520 (15.6%) | 0.13 (1.11) |
| **Staff role** |  |  |  |  |
| Pharmacist | 49,282 (15.9%) | 0.12 (1.43) | 47,135 (16.1%) | 0.09 (1.10) |
| General practitioner (GP) | 208,054 (67.3%) | 0.20 (1.40) | 195,769 (67.1%) | 0.15 (1.11) |
| Nurse | 25,968 (8.4%) | 0.17 (1.37) | 24,871 (8.5%) | 0.13 (1.09) |
| Other | 17,036 (5.5%) | 0.08 (1.49) | 15,816 (5.4%) | 0.07 (1.18) |
| Admin | 3,932 (1.3%) | 0.14 (1.44) | 3,687 (1.3%) | 0.13 (1.11) |
| Missing | 4,935 (1.6%) | 0.19 (1.51) | 4,669 (1.6%) | 0.15 (1.18) |
| **Consultation type** |  |  |  |  |
| Face to face | 131,379 (42.5%) | 0.20 (1.39) | 123,793 (42.4%) | 0.15 (1.12) |
| Telephone | 9,656 (3.1%) | 0.20 (1.41) | 9,093 (3.1%) | 0.14 (1.11) |
| Other | 167,976 (54.3%) | 0.15 (1.43) | 158,890 (54.4%) | 0.11 (1.10) |
| Missing | 196 (0.1%) | 0.35 (2.03) | 171 (0.1%) | 0.20 (1.15) |

# Additional Table S2.5. Top twenty medicines most frequently stopped or started after a medication review.

Includes repeat prescriptions issued in the three months before or three months after a medication review in 2019. N number, BNF British National Formulary, NSAIDs non-steroidal anti-inflammatory drugs.

| Prescribing period with respect to medication review: | **Before only (stopped)** | | **After only (started)** | | **Before and after (continued)** | **Stopped or continued** | | **Started or continued** | |  |
| --- | --- | --- | --- | --- | --- | --- | --- | --- | --- | --- |
|  | Rank | N (%) people | Rank | N (%) people | N (%) people | Rank | N (%) people | Rank | N (%) people | % Change |
| **Drug substance (tablets only)** |  |  |  |  |  |  |  |  |  |  |
| Atorvastatin | 7 | 2,875 (0.9%) | 1 | 7,721 (2.5%) | 77,268 (25.0%) | 1 | 80,143 (25.9%) | 1 | 84,989 (27.5%) | +1.6 |
| Paracetamol | 1 | 5,437 (1.8%) | 2 | 7,158 (2.3%) | 35,793 (11.6%) | 8 | 41,230 (13.3%) | 8 | 42,951 (13.9%) | +0.6 |
| Omeprazole | 2 | 4,339 (1.4%) | 3 | 6,243 (2.0%) | 58,247 (18.8%) | 3 | 62,586 (20.2%) | 2 | 64,490 (20.9%) | +0.6 |
| Amlodipine | 6 | 3,027 (1.0%) | 4 | 4,998 (1.6%) | 55,206 (17.9%) | 4 | 58,233 (18.8%) | 4 | 60,204 (19.5%) | +0.6 |
| Codeine + Paracetamol | 4 | 3,466 (1.1%) | 5 | 3,984 (1.3%) | 24,336 (7.9%) | 13 | 27,802 (9.0%) | 13 | 28,320 (9.2%) | +0.2 |
| Lansoprazole | 8 | 2,092 (0.7%) | 6 | 3,907 (1.3%) | 35,790 (11.6%) | 10 | 37,882 (12.3%) | 10 | 39,697 (12.8%) | +0.6 |
| Bisoprolol | 18 | 1,431 (0.5%) | 7 | 3,596 (1.2%) | 50,280 (16.3%) | 6 | 51,711 (16.7%) | 6 | 53,876 (17.4%) | +0.7 |
| Aspirin | 3 | 3,718 (1.2%) | 8 | 3,529 (1.1%) | 53,031 (17.2%) | 5 | 56,749 (18.4%) | 5 | 56,560 (18.3%) | -0.1 |
| Furosemide | 9 | 2,011 (0.7%) | 9 | 3,137 (1.0%) | 24,682 (8.0%) | 14 | 26,693 (8.6%) | 14 | 27,819 (9.0%) | +0.4 |
| Calcium Carbonate + Colecalciferol | 11 | 1,950 (0.6%) | 10 | 2,851 (0.9%) | 15,935 (5.2%) | 20 | 17,885 (5.8%) | 19 | 18,786 (6.1%) | +0.3 |
| Ramipril | 12 | 1,811 (0.6%) | 11 | 2,737 (0.9%) | 44,266 (14.3%) | 7 | 46,077 (14.9%) | 7 | 47,003 (15.2%) | +0.3 |
| Clopidogrel | 21 | 1,254 (0.4%) | 12 | 2,362 (0.8%) | 22,211 (7.2%) | 15 | 23,465 (7.6%) | 15 | 24,573 (7.9%) | +0.4 |
| Folic Acid | 13 | 1,701 (0.6%) | 13 | 2,258 (0.7%) | 10,450 (3.4%) | 30 | 12,151 (3.9%) | 29 | 12,708 (4.1%) | +0.2 |
| Metformin | 17 | 1,474 (0.5%) | 14 | 2,207 (0.7%) | 33,149 (10.7%) | 11 | 34,623 (11.2%) | 11 | 35,356 (11.4%) | +0.2 |
| Apixaban | 52 | 480 (0.2%) | 15 | 2,198 (0.7%) | 12,028 (3.9%) | 28 | 12,508 (4.0%) | 23 | 14,226 (4.6%) | +0.6 |
| Simvastatin | 5 | 3,455 (1.1%) | 16 | 2,100 (0.7%) | 59,940 (19.4%) | 2 | 63,395 (20.5%) | 3 | 62,040 (20.1%) | -0.4 |
| Alendronic Acid | 15 | 1,635 (0.5%) | 17 | 2,003 (0.6%) | 13,029 (4.2%) | 23 | 14,664 (4.7%) | 22 | 15,032 (4.9%) | +0.1 |
| Tamsulosin | 22 | 1,132 (0.4%) | 18 | 1,962 (0.6%) | 19,902 (6.4%) | 17 | 21,034 (6.8%) | 17 | 21,864 (7.1%) | +0.3 |
| Ferrous Fumarate | 20 | 1,343 (0.4%) | 19 | 1,955 (0.6%) | 5,382 (1.7%) | 46 | 6,725 (2.2%) | 44 | 7,337 (2.4%) | +0.2 |
| Amitriptyline | 16 | 1,558 (0.5%) | 20 | 1,948 (0.6%) | 16,153 (5.2%) | 21 | 17,711 (5.7%) | 20 | 18,101 (5.9%) | +0.1 |
| Ranitidine | 14 | 1,690 (0.5%) | 21 | 1,924 (0.6%) | 10,907 (3.5%) | 27 | 12,597 (4.1%) | 28 | 12,831 (4.1%) | +0.1 |
| Bendroflumethiazide | 10 | 2,001 (0.6%) | 28 | 1,183 (0.4%) | 28,659 (9.3%) | 12 | 30,660 (9.9%) | 12 | 29,842 (9.7%) | -0.3 |
| Warfarin | 19 | 1,352 (0.4%) | 45 | 776 (0.3%) | 13,349 (4.3%) | 22 | 14,701 (4.8%) | 24 | 14,125 (4.6%) | -0.2 |
|  |  |  |  |  |  |  |  |  |  |  |
| **BNF Paragraph** |  |  |  |  |  |  |  |  |  |  |
| 04.07.01 (Non-Opioid Analgesics and Compound Preparations) | 1 | 8,057 (2.6%) | 1 | 10,301 (3.3%) | 65,379 (21.1%) | 8 | 73,436 (23.7%) | 8 | 75,680 (24.5%) | +0.7 |
| 01.03.05 (Proton Pump Inhibitors) | 5 | 5,742 (1.9%) | 2 | 9,433 (3.1%) | 101,846 (32.9%) | 2 | 107,588 (34.8%) | 2 | 111,279 (36.0%) | +1.2 |
| 21.22.00 (Emollients) | 3 | 6,703 (2.2%) | 3 | 8,579 (2.8%) | 15,949 (5.2%) | 21 | 22,652 (7.3%) | 19 | 24,528 (7.9%) | +0.6 |
| 13.02.01 (Emollients) | 4 | 6,703 (2.2%) | 4 | 8,579 (2.8%) | 15,949 (5.2%) | 22 | 22,652 (7.3%) | 20 | 24,528 (7.9%) | +0.6 |
| 03.01.01 (Adrenoceptor Agonists) | 2 | 6,807 (2.2%) | 5 | 8,391 (2.7%) | 28,748 (9.3%) | 15 | 35,555 (11.5%) | 15 | 37,139 (12.0%) | +0.5 |
| 02.12.04 (Statins) | 17 | 2,888 (0.9%) | 6 | 7,746 (2.5%) | 77,147 (24.9%) | 5 | 80,035 (25.9%) | 4 | 84,893 (27.5%) | +1.6 |
| 09.06.04 (Vitamin D) | 9 | 4,015 (1.3%) | 7 | 6,998 (2.3%) | 43,941 (14.2%) | 9 | 47,956 (15.5%) | 9 | 50,939 (16.5%) | +1.0 |
| 01.06.04 (Osmotic Laxatives) | 6 | 4,457 (1.4%) | 8 | 6,041 (2.0%) | 14,707 (4.8%) | 25 | 19,164 (6.2%) | 25 | 20,748 (6.7%) | +0.5 |
| 02.06.02 (Calcium-Channel Blockers) | 10 | 3,994 (1.3%) | 9 | 5,711 (1.8%) | 82,129 (26.6%) | 3 | 86,123 (27.9%) | 3 | 87,840 (28.4%) | +0.6 |
| 02.05.05 (Renin-Angiotensin System Drugs) | 11 | 3,919 (1.3%) | 10 | 5,281 (1.7%) | 122,836 (39.7%) | 1 | 126,755 (41.0%) | 1 | 128,117 (41.4%) | +0.4 |
| 03.02.00 (Corticosteroids (Respiratory)) | 7 | 4,232 (1.4%) | 11 | 4,949 (1.6%) | 32,831 (10.6%) | 14 | 37,063 (12.0%) | 14 | 37,780 (12.2%) | +0.2 |
| 11.08.01 (Tear Deficiency, Eye Lubricant and Astringent) | 13 | 3,790 (1.2%) | 12 | 4,866 (1.6%) | 22,704 (7.3%) | 18 | 26,494 (8.6%) | 18 | 27,570 (8.9%) | +0.3 |
| 02.09.00 (Antiplatelet Drugs) | 12 | 3,917 (1.3%) | 13 | 4,680 (1.5%) | 74,287 (24.0%) | 7 | 78,204 (25.3%) | 7 | 78,967 (25.5%) | +0.2 |
| 09.01.02 (Drugs Used in Megaloblastic Anaemias) | 14 | 3,425 (1.1%) | 14 | 4,510 (1.5%) | 13,563 (4.4%) | 27 | 16,988 (5.5%) | 27 | 18,073 (5.8%) | +0.4 |
| 02.04.00 (Beta-Adrenoceptor Blocking Drugs) | 21 | 2,316 (0.7%) | 15 | 3,992 (1.3%) | 76,727 (24.8%) | 6 | 79,043 (25.6%) | 6 | 80,719 (26.1%) | +0.5 |
| 02.06.01 (Nitrates) | 15 | 3,141 (1.0%) | 16 | 3,925 (1.3%) | 13,681 (4.4%) | 28 | 16,822 (5.4%) | 28 | 17,606 (5.7%) | +0.3 |
| 10.03.02 (Rubefacients, Topical NSAIDs, Capsaicin and Poultices) | 16 | 3,065 (1.0%) | 17 | 3,872 (1.3%) | 11,342 (3.7%) | 32 | 14,407 (4.7%) | 31 | 15,214 (4.9%) | +0.3 |
| 02.08.02 (Oral Anticoagulants) | 36 | 1,199 (0.4%) | 18 | 3,551 (1.1%) | 36,970 (12.0%) | 13 | 38,169 (12.3%) | 11 | 40,521 (13.1%) | +0.8 |
| 12.02.01 (Drugs Used in Nasal Allergy) | 18 | 2,793 (0.9%) | 19 | 3,513 (1.1%) | 10,485 (3.4%) | 37 | 13,278 (4.3%) | 36 | 13,998 (4.5%) | +0.2 |
| 02.12.00 (Lipid-Regulating Drugs) | 8 | 4,228 (1.4%) | 20 | 3,498 (1.1%) | 77,279 (25.0%) | 4 | 81,507 (26.4%) | 5 | 80,777 (26.1%) | -0.2 |
| 02.02.01 (Thiazides and Related Diuretics) | 19 | 2,664 (0.9%) | 30 | 2,207 (0.7%) | 37,647 (12.2%) | 11 | 40,311 (13.0%) | 13 | 39,854 (12.9%) | -0.1 |
| 03.01.02 (Antimuscarinic Bronchodilators) | 20 | 2,481 (0.8%) | 38 | 1,650 (0.5%) | 12,582 (4.1%) | 30 | 15,063 (4.9%) | 35 | 14,232 (4.6%) | -0.3 |
|  |  |  |  |  |  |  |  |  |  |  |
| **BNF Chapter** |  |  |  |  |  |  |  |  |  |  |
| Chapter 01 (Gastro-Intestinal System) | 2 | 7,870 (2.5%) | 1 | 12,453 (4.0%) | 130,197 (42.1%) | 2 | 138,067 (44.7%) | 2 | 142,650 (46.1%) | +1.5 |
| Chapter 04 (Central Nervous System) | 3 | 7,303 (2.4%) | 2 | 11,151 (3.6%) | 126,663 (41.0%) | 3 | 133,966 (43.3%) | 3 | 137,814 (44.6%) | +1.2 |
| Chapter 09 (Nutrition and Blood) | 4 | 6,818 (2.2%) | 3 | 11,040 (3.6%) | 61,318 (19.8%) | 5 | 68,136 (22.0%) | 5 | 72,358 (23.4%) | +1.4 |
| Chapter 13 (Skin) | 1 | 7,899 (2.6%) | 4 | 10,112 (3.3%) | 21,340 (6.9%) | 10 | 29,239 (9.5%) | 10 | 31,452 (10.2%) | +0.7 |
| Chapter 02 (Cardiovascular System) | 10 | 3,596 (1.2%) | 5 | 7,710 (2.5%) | 239,557 (77.5%) | 1 | 243,153 (78.6%) | 1 | 247,267 (80.0%) | +1.3 |
| Chapter 03 (Respiratory System) | 6 | 4,861 (1.6%) | 6 | 7,111 (2.3%) | 58,316 (18.9%) | 6 | 63,177 (20.4%) | 6 | 65,427 (21.2%) | +0.7 |
| Chapter 06 (Endocrine System) | 8 | 3,928 (1.3%) | 7 | 6,537 (2.1%) | 102,990 (33.3%) | 4 | 106,918 (34.6%) | 4 | 109,527 (35.4%) | +0.8 |
| Chapter 10 (Musculoskeletal and Joint Diseases) | 5 | 4,941 (1.6%) | 8 | 6,296 (2.0%) | 36,940 (11.9%) | 8 | 41,881 (13.5%) | 8 | 43,236 (14.0%) | +0.4 |
| Chapter 11 (Eye) | 7 | 4,094 (1.3%) | 9 | 5,608 (1.8%) | 36,785 (11.9%) | 9 | 40,879 (13.2%) | 9 | 42,393 (13.7%) | +0.5 |
| Chapter 07 (Obstetrics, Gynaecology and Urinary-Tract Disorders) | 9 | 3,634 (1.2%) | 10 | 5,441 (1.8%) | 40,220 (13.0%) | 7 | 43,854 (14.2%) | 7 | 45,661 (14.8%) | +0.6 |
| Chapter 12 (Ear, Nose and Oropharynx) | 11 | 3,228 (1.0%) | 11 | 4,090 (1.3%) | 12,392 (4.0%) | 11 | 15,620 (5.1%) | 11 | 16,482 (5.3%) | +0.3 |
| Chapter 05 (Infections) | 12 | 2,315 (0.7%) | 12 | 2,523 (0.8%) | 12,873 (4.2%) | 12 | 15,188 (4.9%) | 12 | 15,396 (5.0%) | +0.1 |
| Chapter 99 (Chapter Unknown) | 13 | 1,313 (0.4%) | 13 | 1,710 (0.6%) | 2,693 (0.9%) | 14 | 4,006 (1.3%) | 14 | 4,403 (1.4%) | +0.1 |
| Chapter 08 (Malignant Disease and Immunosuppression) | 14 | 555 (0.2%) | 14 | 854 (0.3%) | 7,119 (2.3%) | 13 | 7,674 (2.5%) | 13 | 7,973 (2.6%) | +0.1 |

# Additional Figure S2.1. Distribution of change (difference) in prescription count before (A) and after (B) dropping extreme values.

People were dropped if they had 0 prescriptions in the three months before their medication review and if their change in prescription count was >3 standard deviations from the mean. Change in prescription count is calculated as the maximum prescription count in the three months after a medication review minus the maximum prescription count in the three months before a medication review.


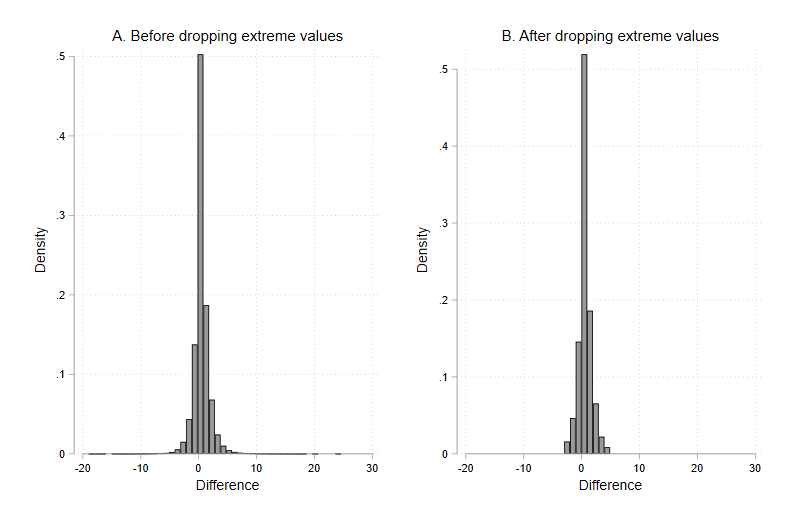


# Additional Table S2.6. Cross-tabulation of maximum prescription count before vs after a medication review.

Categorised maximum prescription count in the three months before vs three months after a medication review.

|  | Before review | | | | | | |
| --- | --- | --- | --- | --- | --- | --- | --- |
| After review | 1 | 2-4 | 5-9 | 10-14 | 15-19 | 20+ | Total |
| 0 | 1,349 (5.8%) | 1,013 (1.0%) | .. | .. | .. | .. | 2,362 (0.8%) |
| 1 | 15,215 (65.4%) | 3,510 (3.4%) | .. | .. | .. | .. | 18,725 (6.4%) |
| 2-4 | 6,395 (27.5%) | 84,217 (82.0%) | 8,914 (7.1%) | .. | .. | .. | 99,526 (34.1%) |
| 5-9 | 302 (1.3%) | 14,018 (13.6%) | 109,762 (86.9%) | 5,404 (15.9%) | .. | .. | 129,486 (44.4%) |
| 10-14 | .. | .. | 7,660 (6.1%) | 27,128 (79.6%) | 1,182 (23.7%) | .. | 35,970 (12.3%) |
| 15-19 | .. | .. | .. | 1,548 (4.5%) | 3,634 (72.8%) | 131 (25.3%) | 5,313 (1.8%) |
| 20+ | .. | .. | .. | .. | 179 (3.6%) | 386 (74.7%) | 565 (0.2%) |
| Total | 23,261 (100%) | 102,758 (100%) | 126,336 (100%) | 34,080 (100%) | 4,995 (100%) | 517 (100%) | 291,947 (100%) |

# Additional Figure S2.2. Bar charts showing the mean change in prescription count by demographic and other characteristics.

Change in prescription count is calculated as the maximum prescription count in the three months after a medication review minus the maximum prescription count in the three months before a medication review. GP general practitioner.


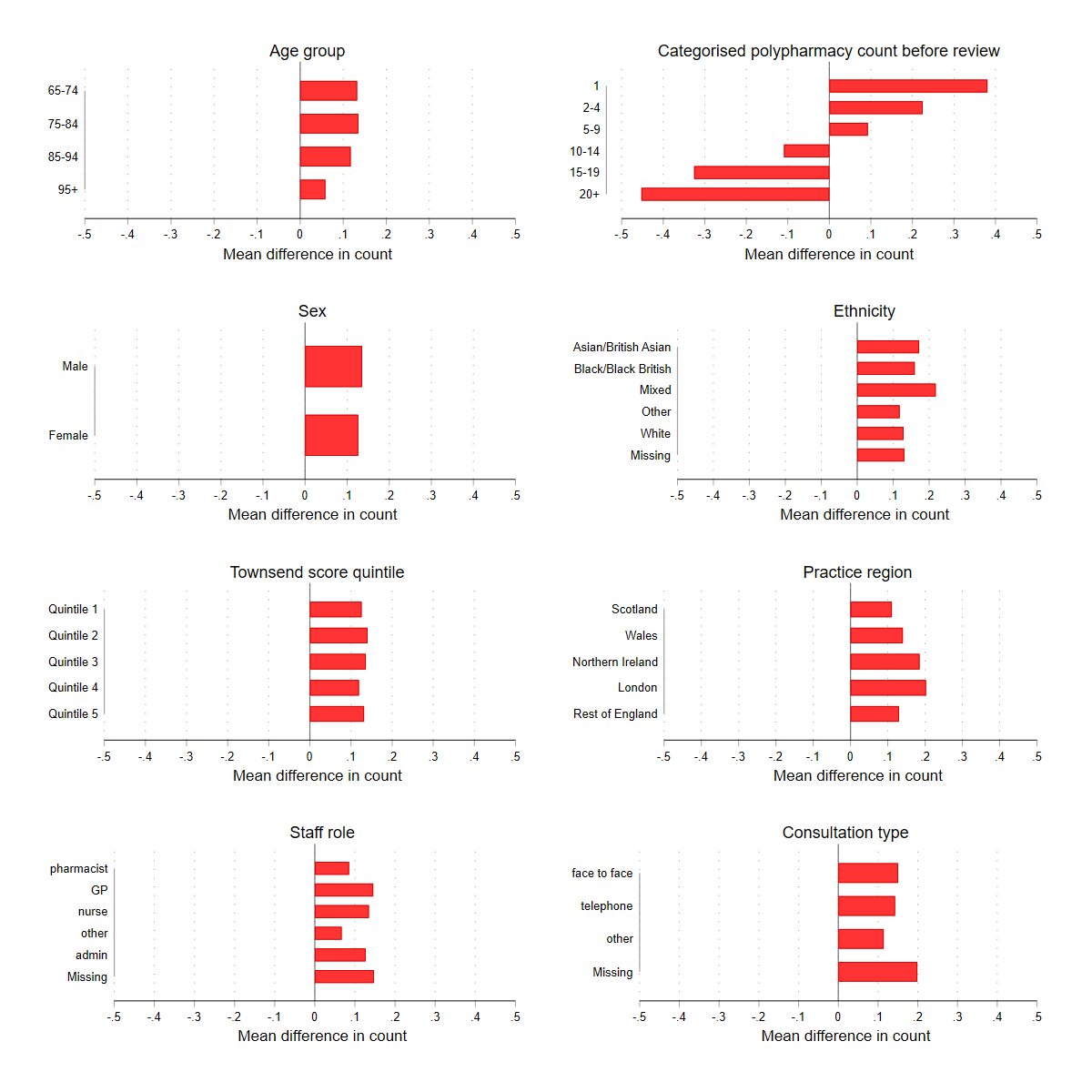

Supplement: Supplementary file 2 — Additional file 2: Additional Table S2.1. Full baseline characteristics of the study population as of 01 January 2019. Additional Table S2.2. Full results, association between baseline factors and having a medication review in 2019, Cox regression. Additional Table S2.3. Sensitivity analysis: association between baseline factors and having a medication review in 2019, Cox regression using conservative definition of medication reviews. Additional Table S2.4. Characteristics and mean change in prescription count for people with a medication review and at least three months of subsequent follow-up. Additional Table S2.5. Top twenty medicines most frequently stopped or started after a medication review. Additional Figure S2.1. Distribution of change (difference) in prescription count before (A) and after (B) dropping extreme values. Additional Table S2.6. Cross-tabulation of maximum prescription count before vs after a medication review. Additional Figure S2.2. Bar charts showing the mean change in prescription count by demographic and other characteristics. [file 12877_2023_4143_MOESM2_ESM.docx]
