# Supplementary material for: Frequency and impact of medication reviews for people aged 65 years or above in UK primary care: an observational study using electronic health records
Source: BMC Geriatr. 2023 Jul 14;23:435. doi: 10.1186/s12877-023-04143-2 (PMC10347807; doi:10.1186/s12877-023-04143-2)

Additional File 3

Additional figures displaying changes in the types of medicines prescribed before and after a medication review, including results for specific chapters of the British National Formulary (BNF) and sensitivity analyses.

Contents

[Additional Figure S3.1. Medicines prescribed in the three months before and/or after a medication review – **BNF paragraph-level**. 2](#_Toc129620896)

[Additional Figure S3.2. Medicines prescribed in the three months before and/or after a medication review – **BNF chapter-level**. 2](#_Toc129620897)

[Additional Figure S3.3. Medicines prescribed in the three months before and/or after a medication review – **all formulations**. 3](#_Toc129620898)

[Additional Figure S3.4. Medicines prescribed in the three months before and/or after a medication review – **all prescription types**. 4](#_Toc129620899)

[Additional Figure S3.5. Medicines prescribed in the three months before and/or after a medication review, **by BNF chapter**. 5](#_Toc129620900)

[Additional Figure S3.6. **Psychotropic medicines** prescribed in the three months before and/or after a medication review. 12](#_Toc129620901)

[Additional Figure S3.7. **Opioids** prescribed in the three months before and/or after a medication review. 12](#_Toc129620902)

[Additional Figure S3.8. **Anticholinergic medicines** prescribed in the three months before and/or after a medication review. 13](#_Toc129620903)

[Additional Figure S3.9. **Gabapentinoids** prescribed in the three months before and/or after a medication review. 13](#_Toc129620904)

[Additional Figure S3.10. Medicines prescribed in the **six** **months** before and/or after a medication review. 14](#_Toc129620905)

[Additional Figure S3.11. Medicines prescribed in the **three months** before and/or **one-four** months after a medication review. 14](#_Toc129620906)

[Additional Figure S3.12. Medicines prescribed in the **one month** before and/or after a medication review. 15](#_Toc129620907)

[Additional Figure S3.13. Medicines prescribed in the three months before and/or after an **in-person** medication review. 15](#_Toc129620908)

[Additional Figure S3.14. Medicines prescribed in the three months before and/or after a medication review – **most frequently prescribed**. 16](#_Toc129620909)

[Additional Figure S3.15. Medicines prescribed in the three months before and/or after a medication review – **most frequently prescribed, BNF paragraph-level**. 16](#_Toc129620910)

[Additional Figure S3.16. Medicines prescribed in the three months before and/or after a medication review – **most frequently prescribed, BNF chapter-level**. 17](#_Toc129620911)

# Additional Figure S3.1. Medicines prescribed in the three months before and/or after a medication review – **BNF paragraph-level**.

The figure is sorted according to the medicines most frequently ‘started’, i.e., prescribed only after the medication review. The top 20 most frequently ‘stopped’ or ‘started’ medicines are shown. Only prescriptions issued as a repeat prescription are included. BNF British National Formulary.


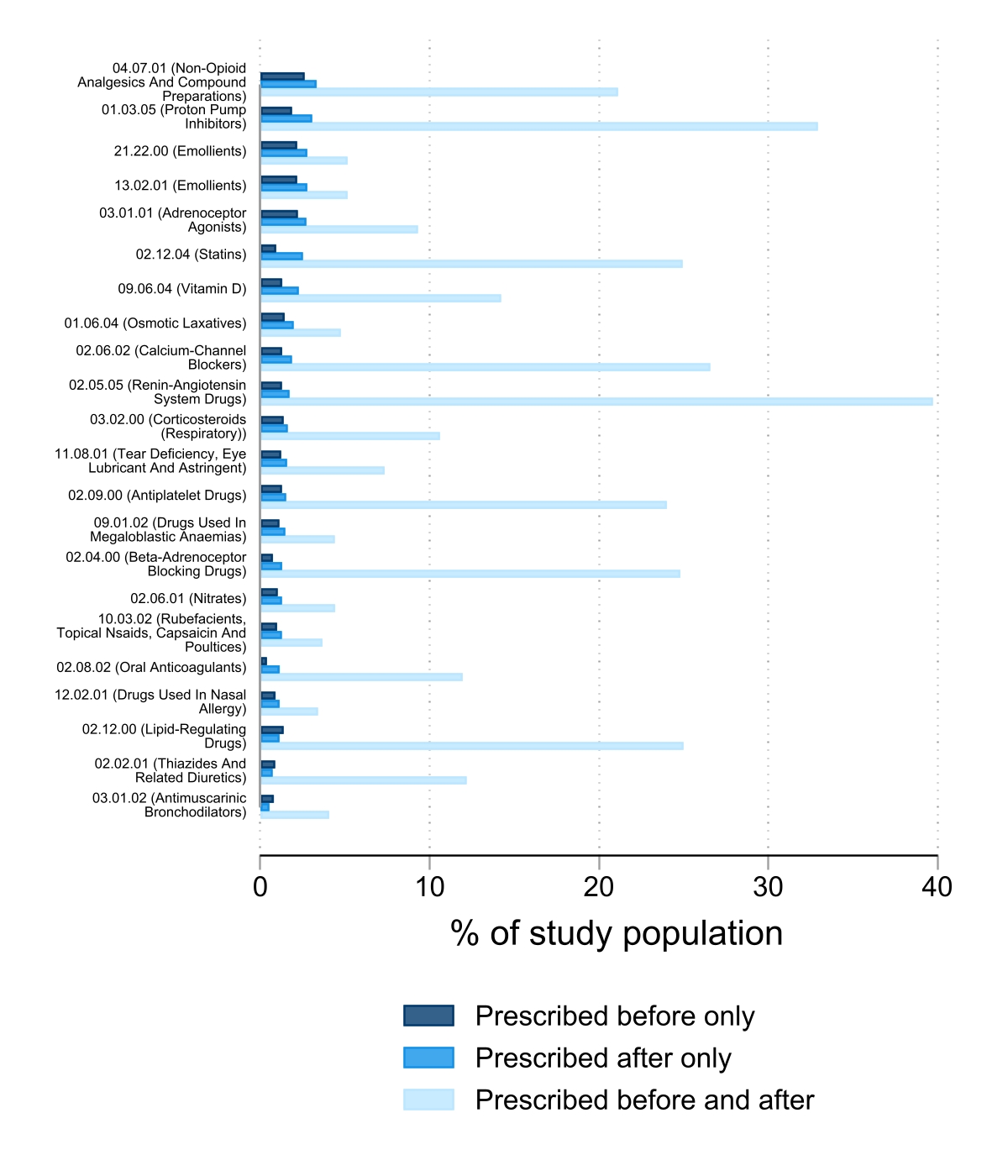


# Additional Figure S3.2. Medicines prescribed in the three months before and/or after a medication review – **BNF chapter-level**.

The figure is sorted according to the medicines most frequently ‘started’, i.e., prescribed only after the medication review. Only prescriptions issued as a repeat prescription are included. BNF British National Formulary.


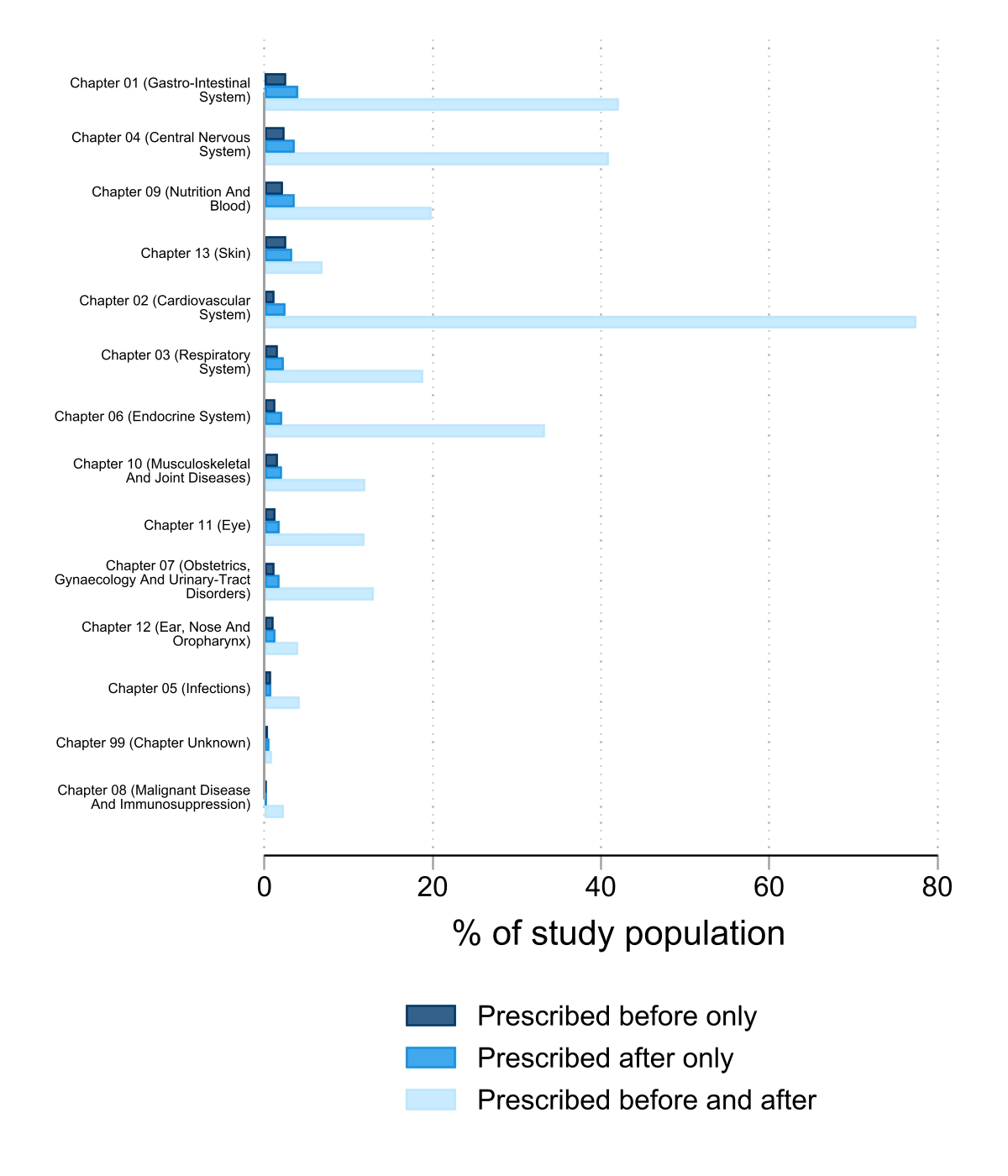


# Additional Figure S3.3. Medicines prescribed in the three months before and/or after a medication review – **all formulations**.

The figure is sorted according to the medicines most frequently ‘started’, i.e., prescribed only after the medication review. The top 20 most frequently ‘stopped’ or ‘started’ medicines are shown. Only prescriptions issued as a repeat prescription are included.


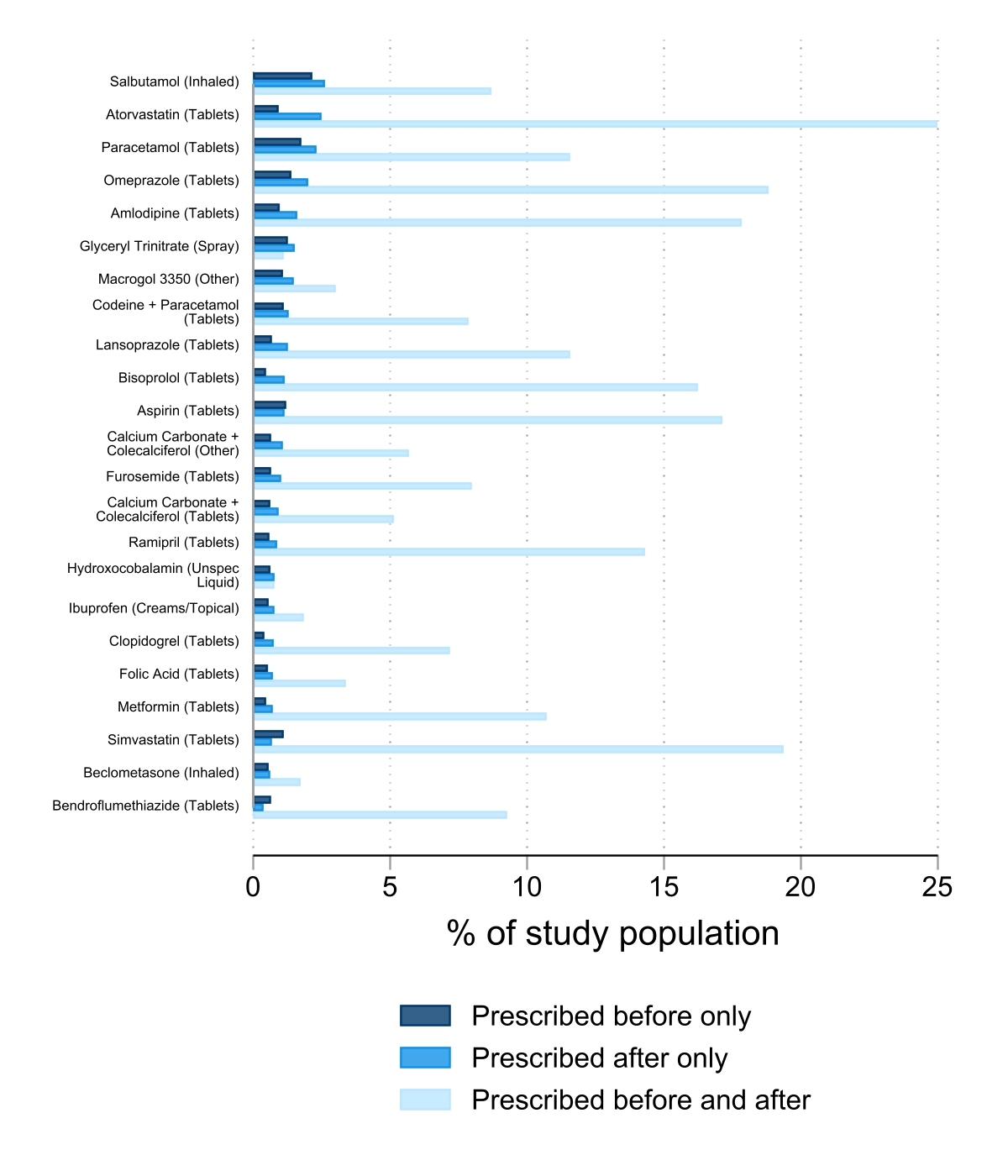


# Additional Figure S3.4. Medicines prescribed in the three months before and/or after a medication review – **all prescription types**.

The figure is sorted according to the medicines most frequently ‘started’, i.e., prescribed only after the medication review. The top 20 most frequently ‘stopped’ or ‘started’ medicines are shown. These figures include prescriptions issued as part of a repeat script and prescriptions that are not part of a repeat script.


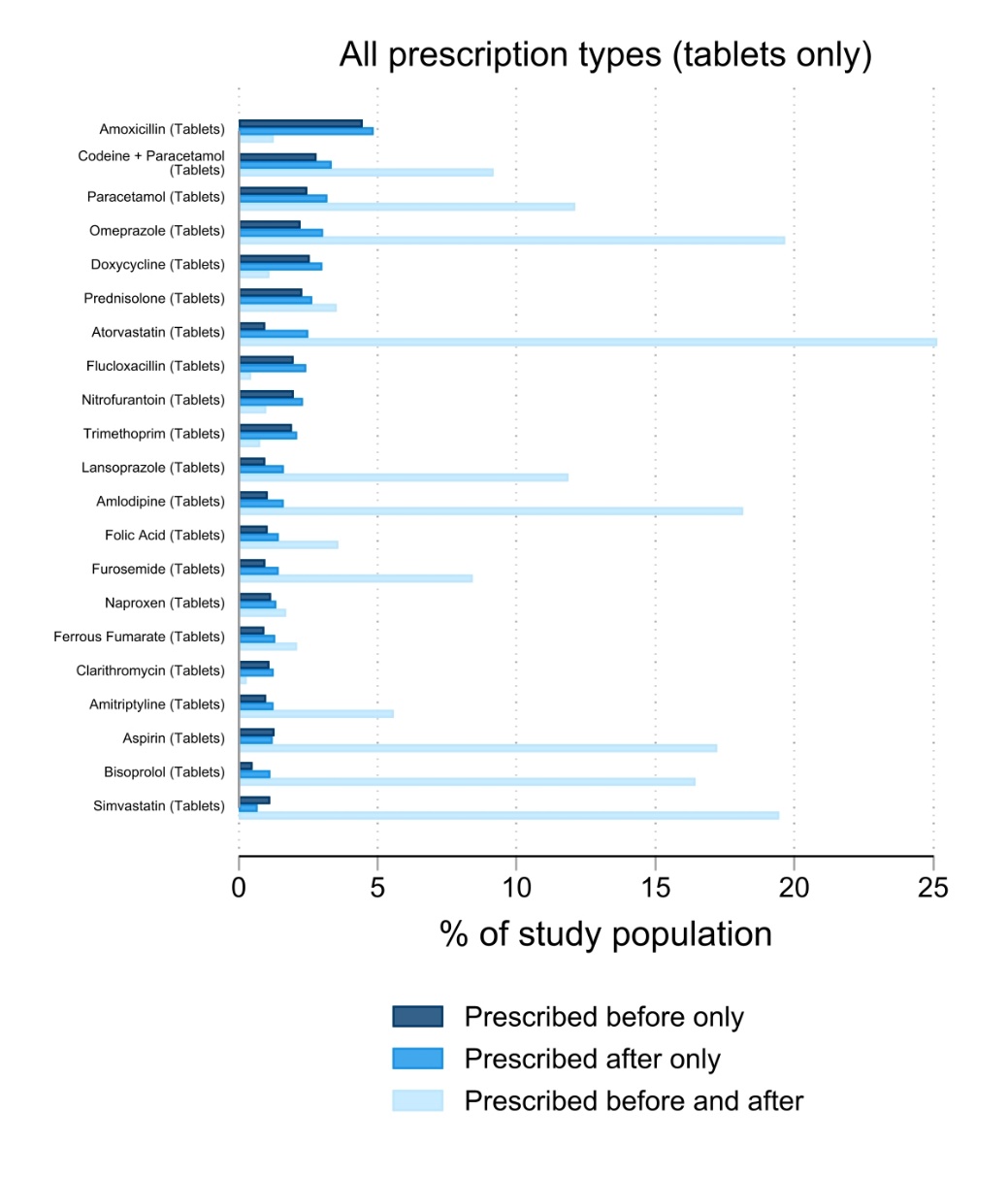


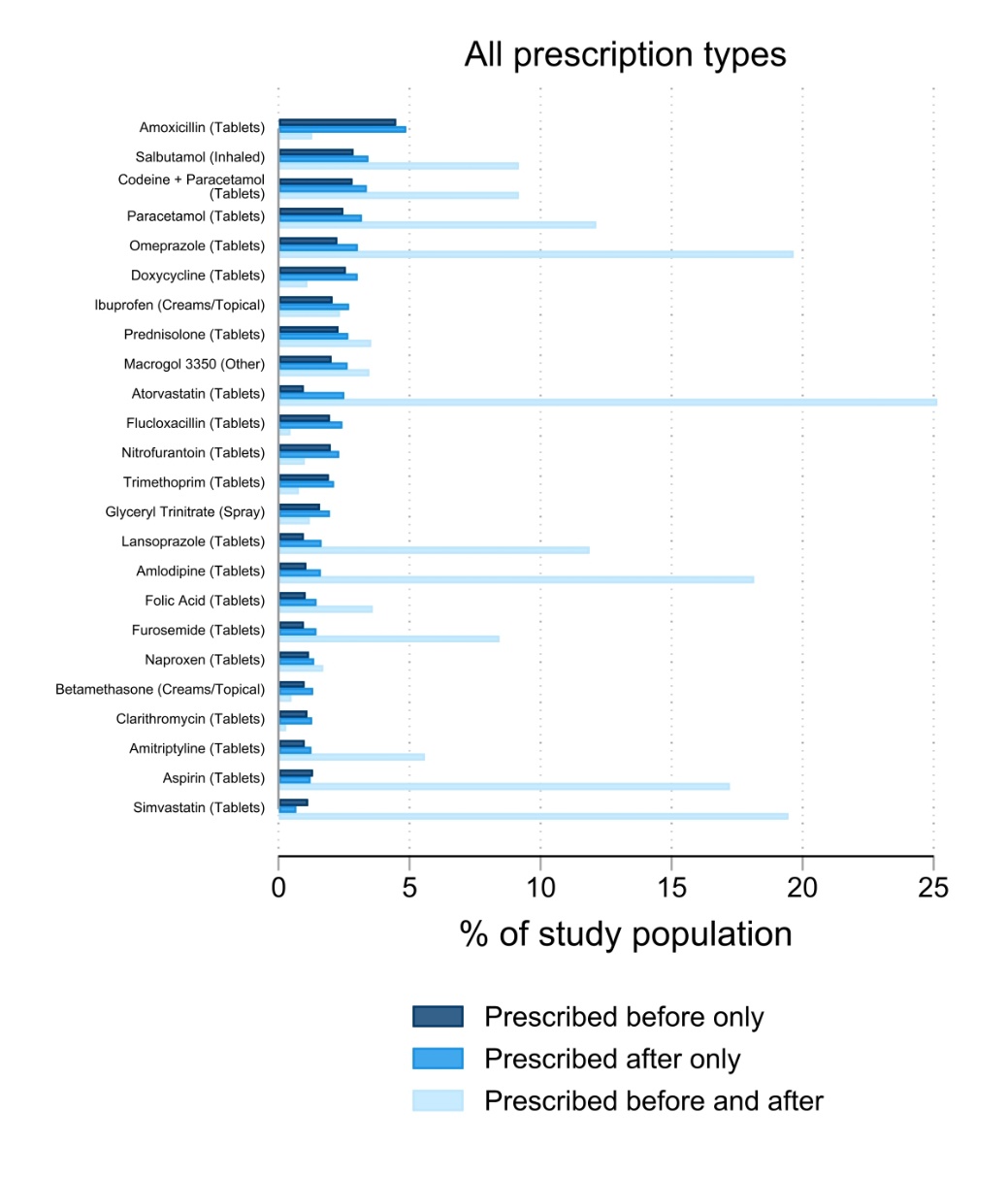


# Additional Figure S3.5. Medicines prescribed in the three months before and/or after a medication review, **by BNF chapter**.

The figures are sorted according to the medicines most frequently ‘started’, i.e., prescribed only after the medication review. The top 15 most frequently ‘stopped’ or ‘started’ medicines are shown. Only prescriptions issued as a repeat prescription are included. BNF British National Formulary.


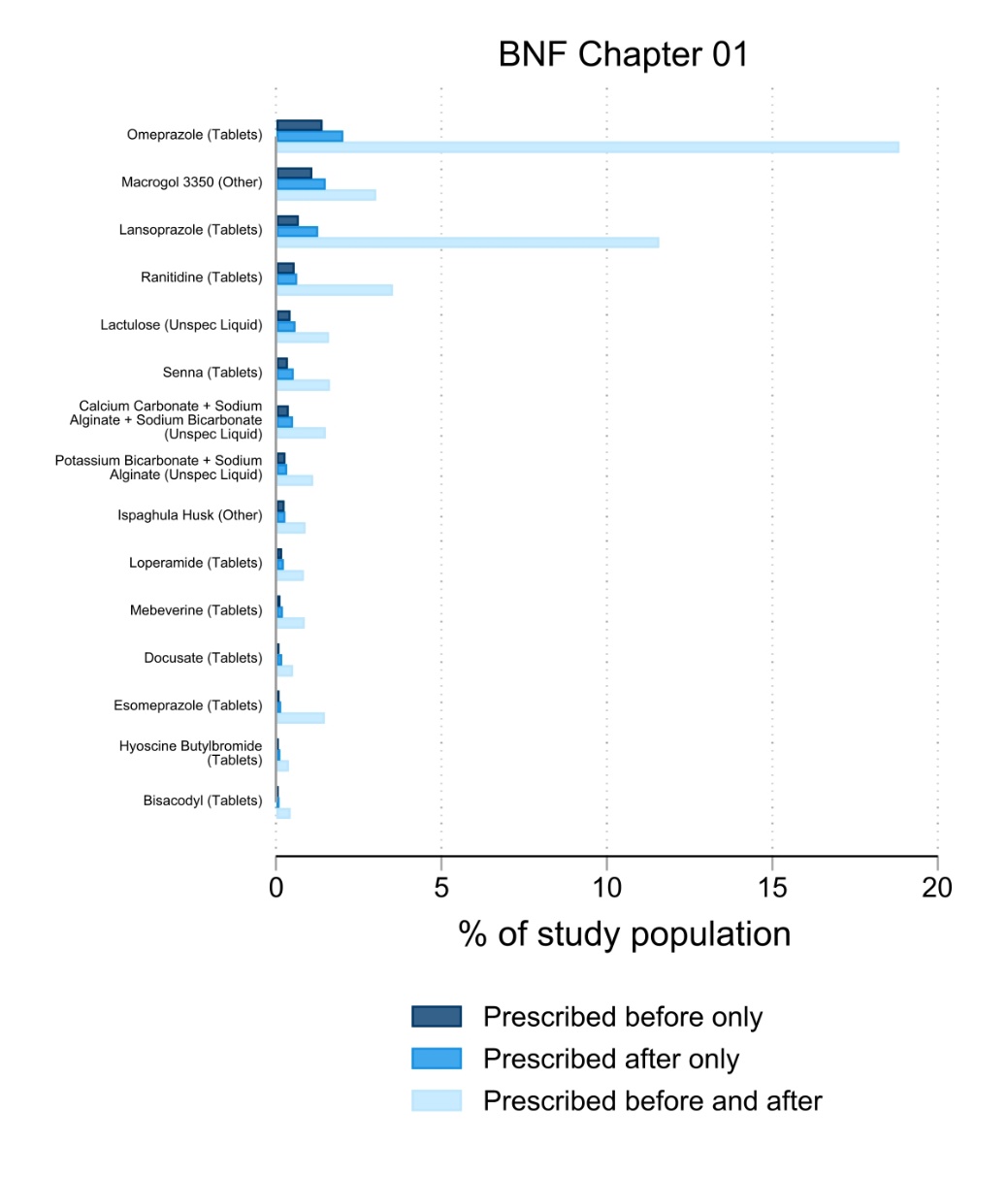


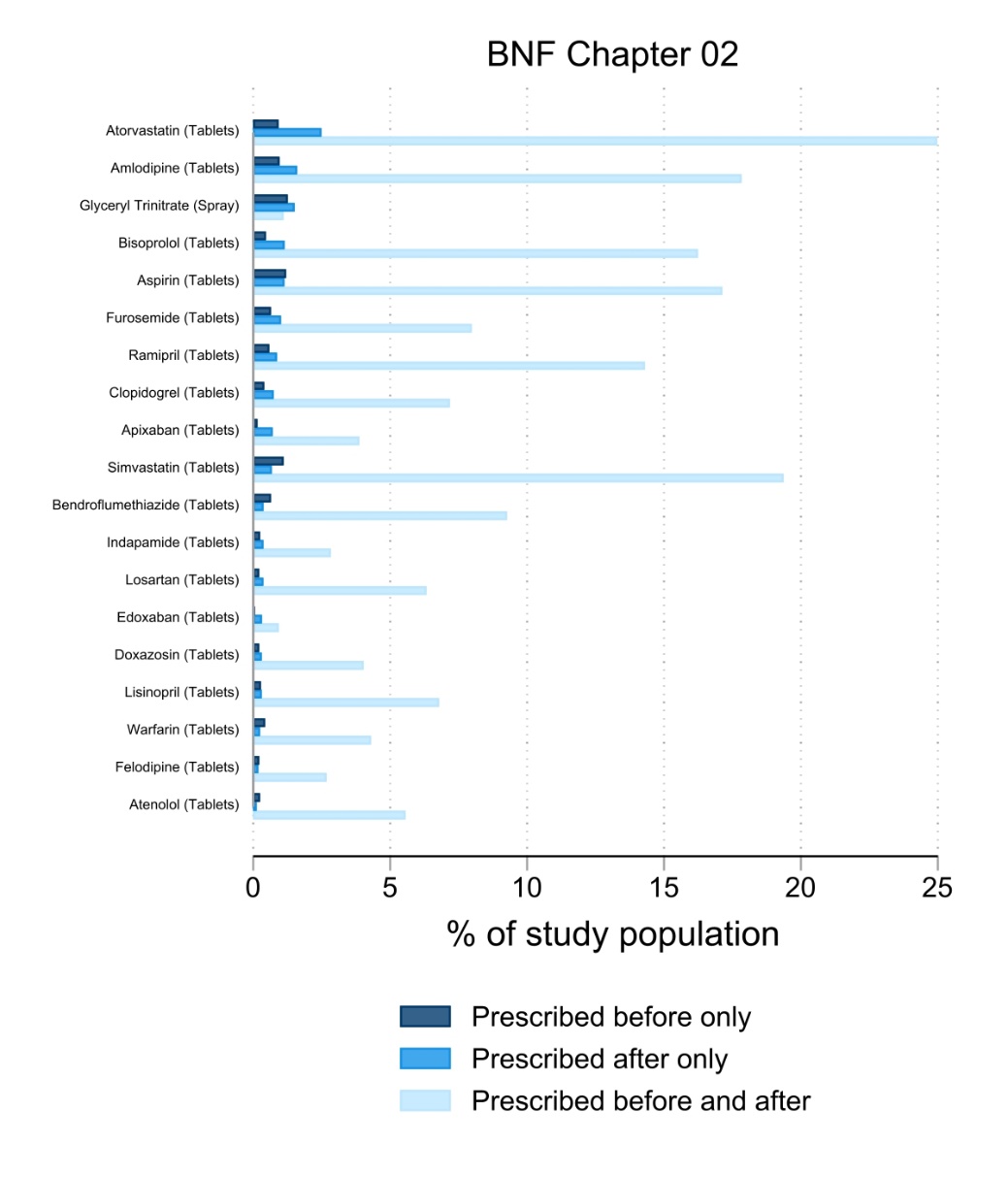


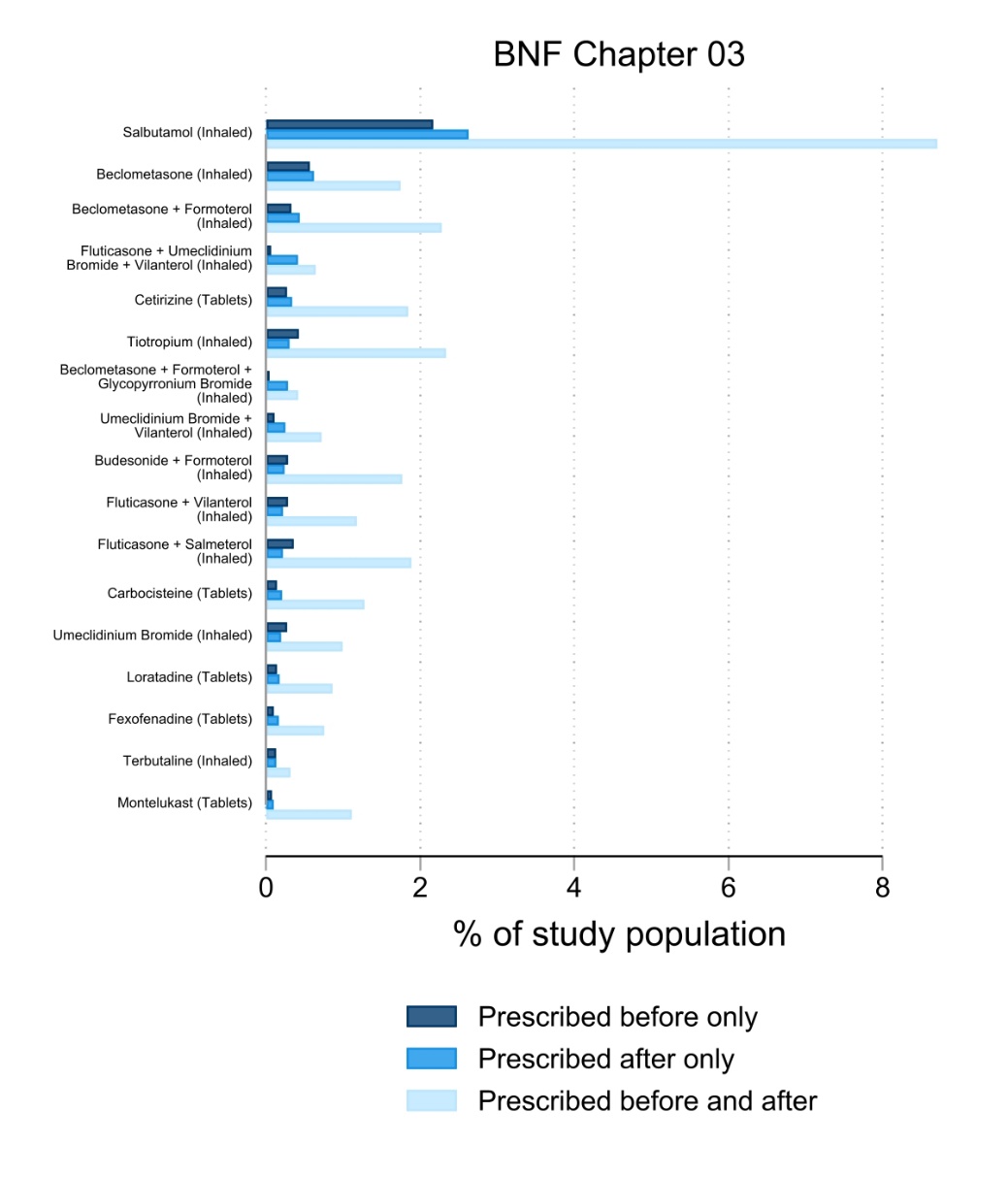


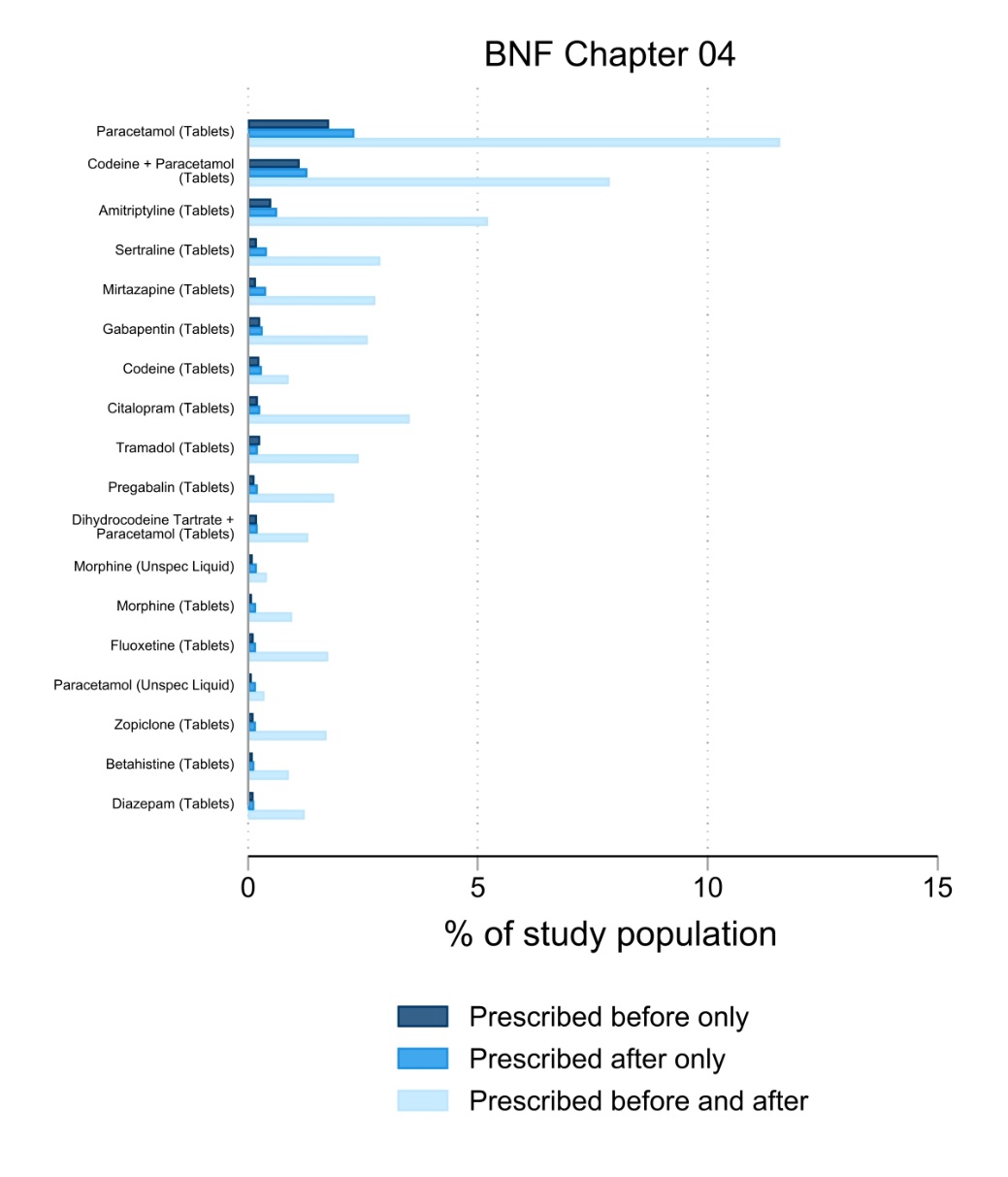


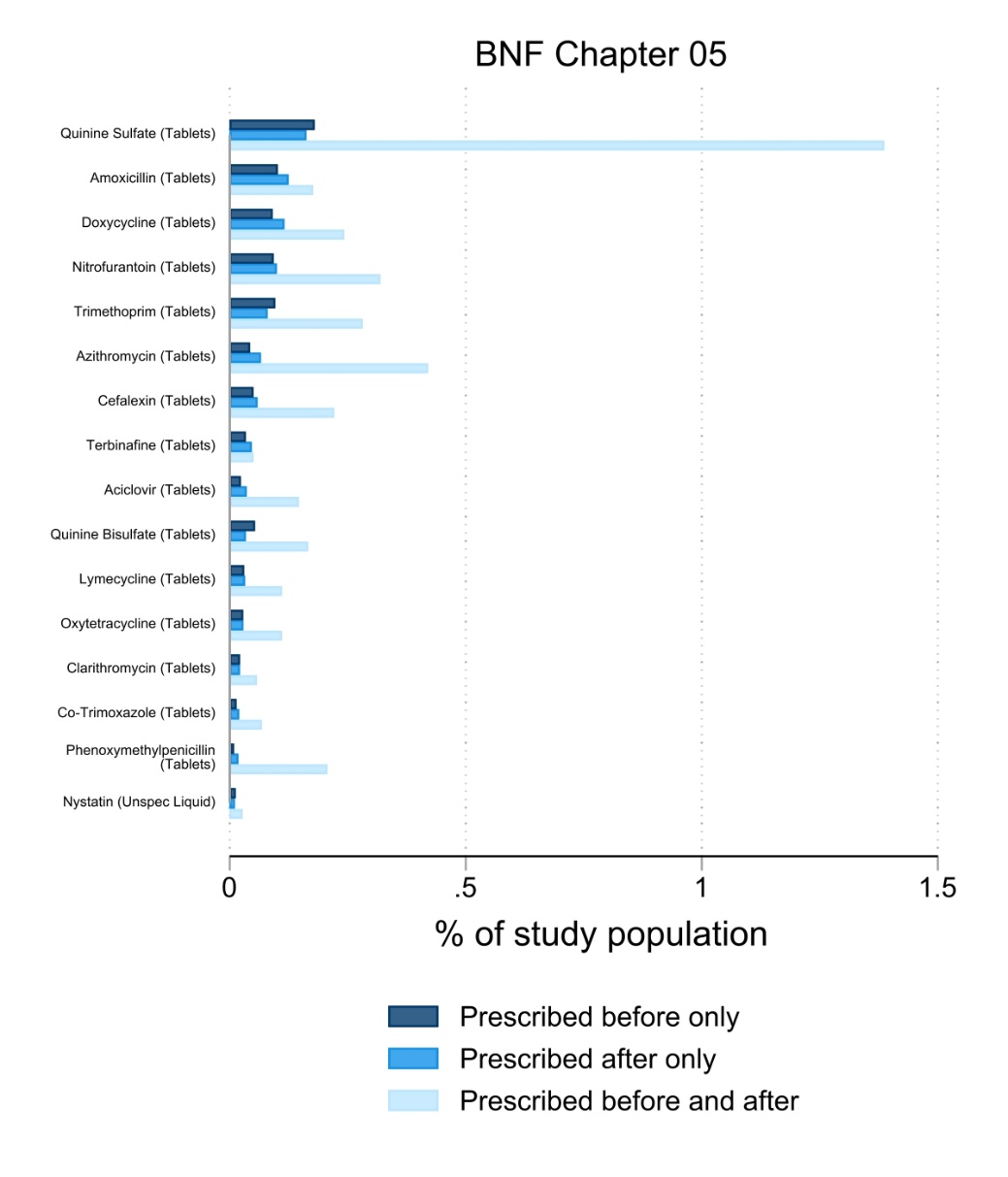


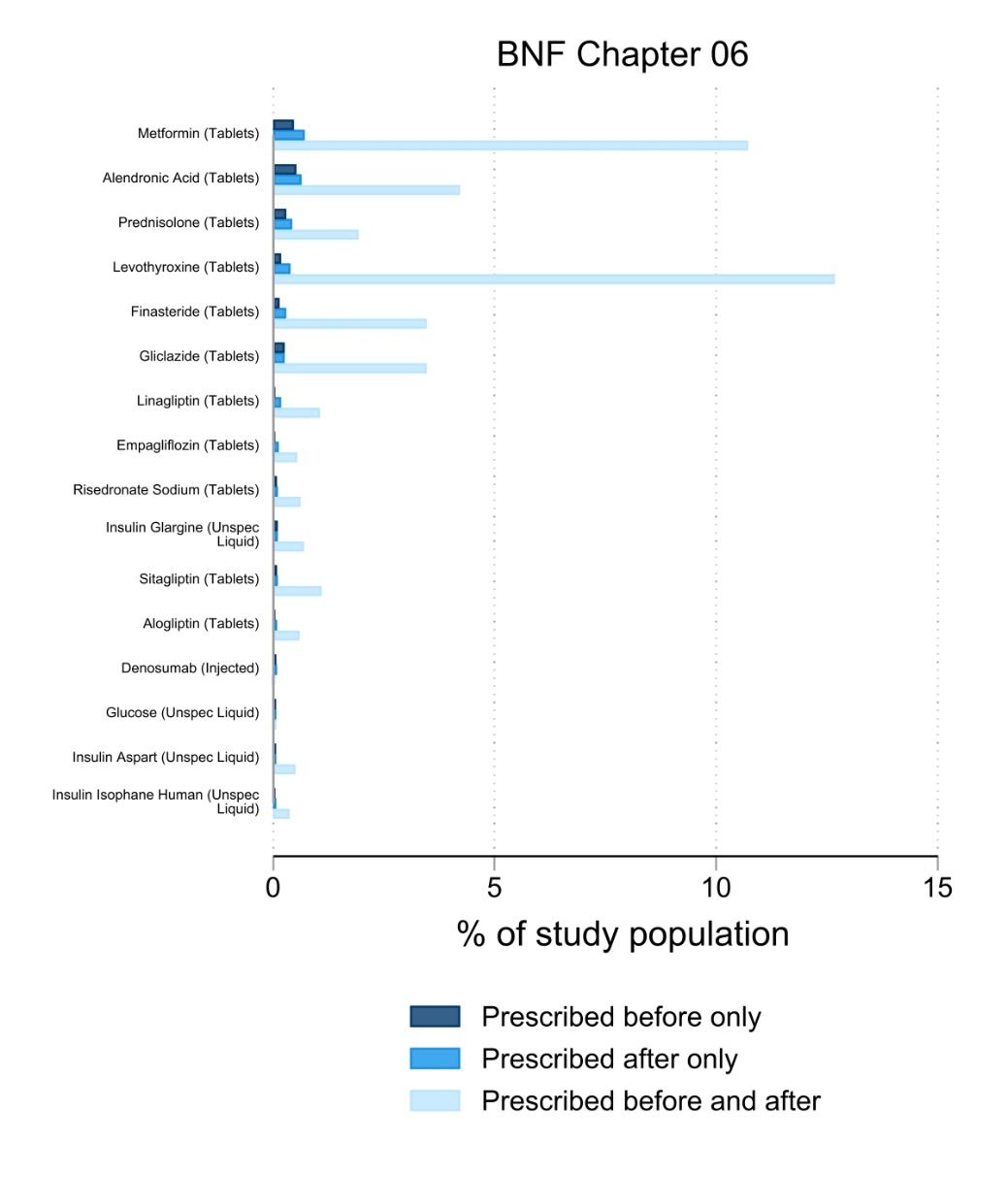


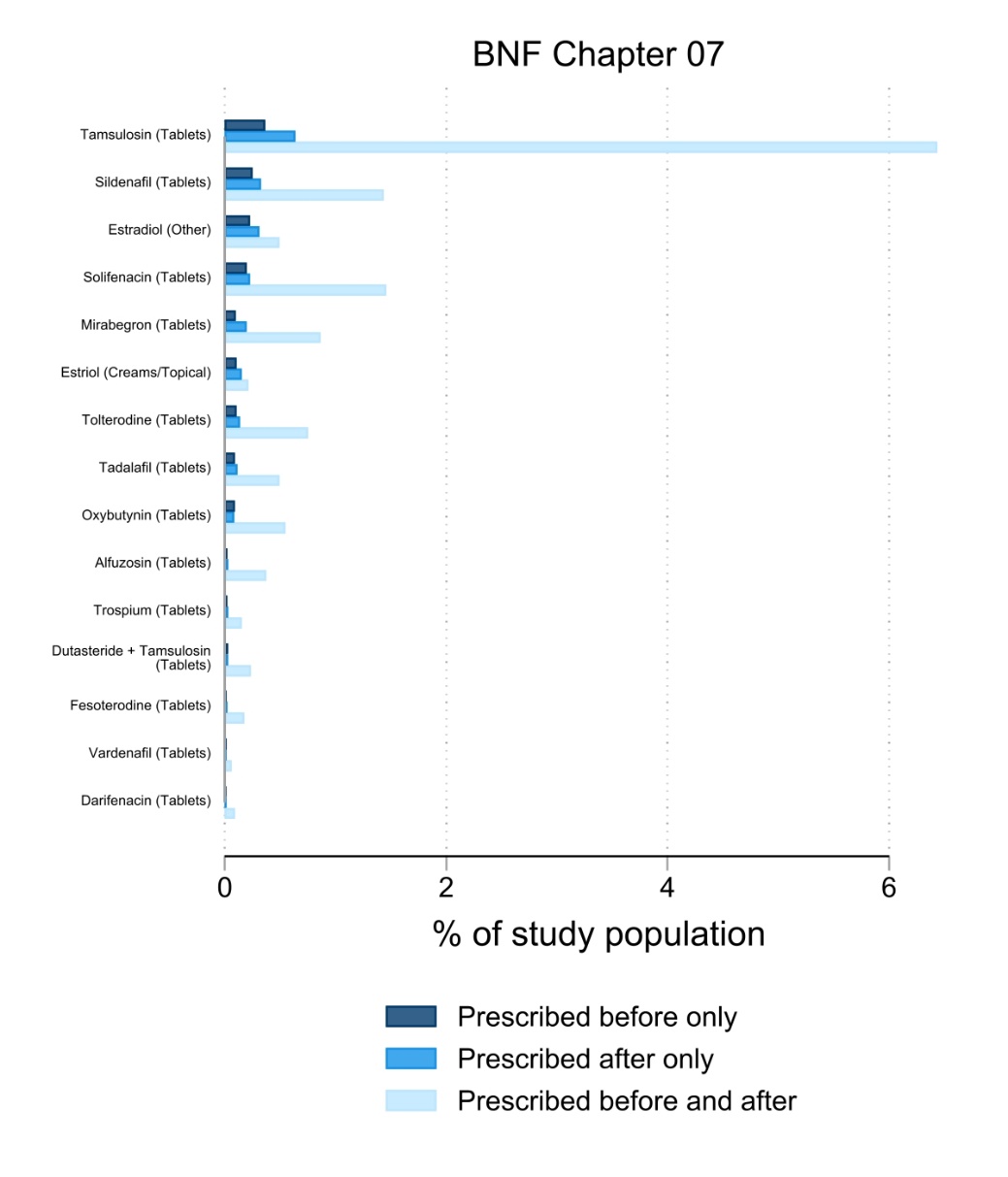


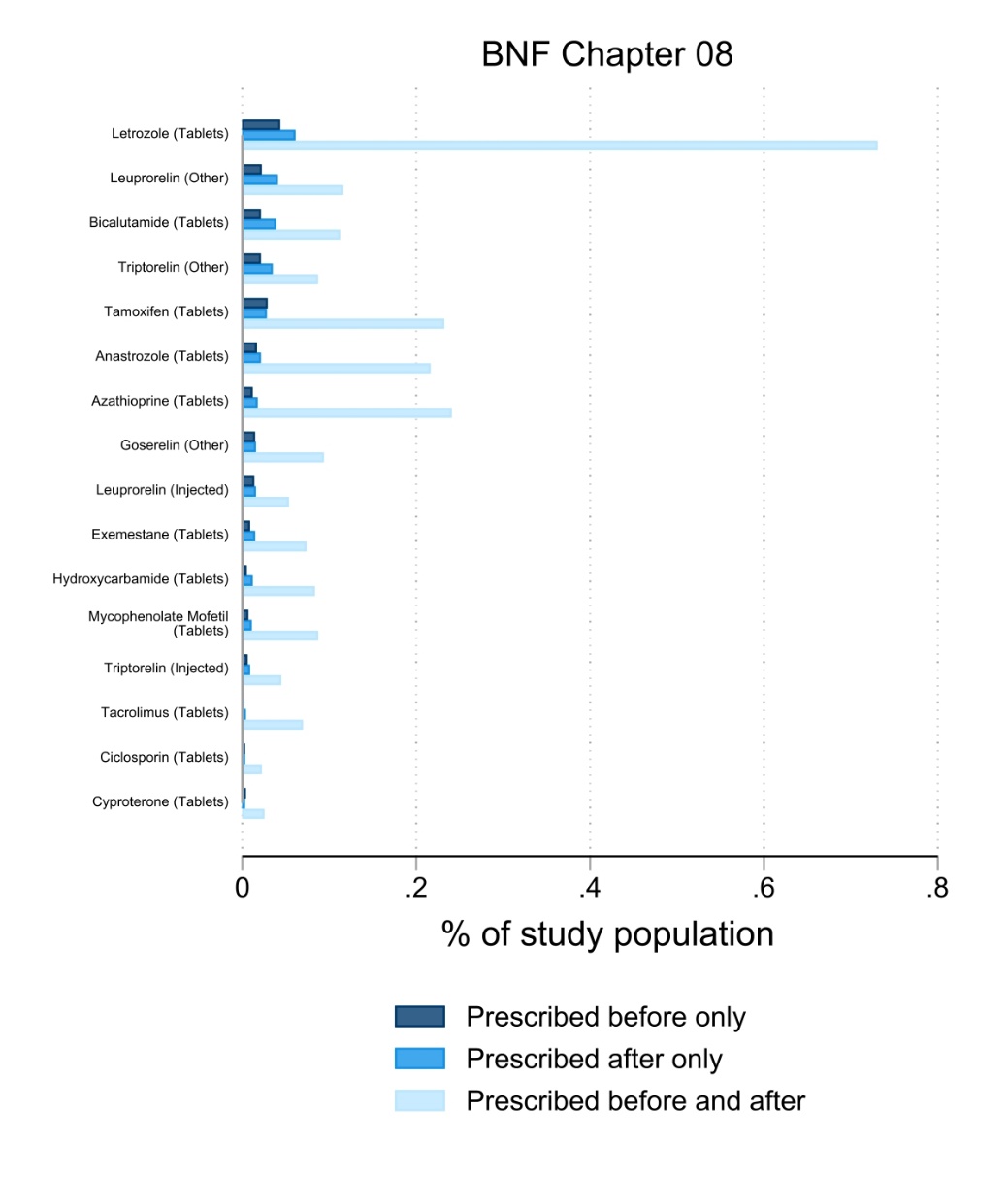


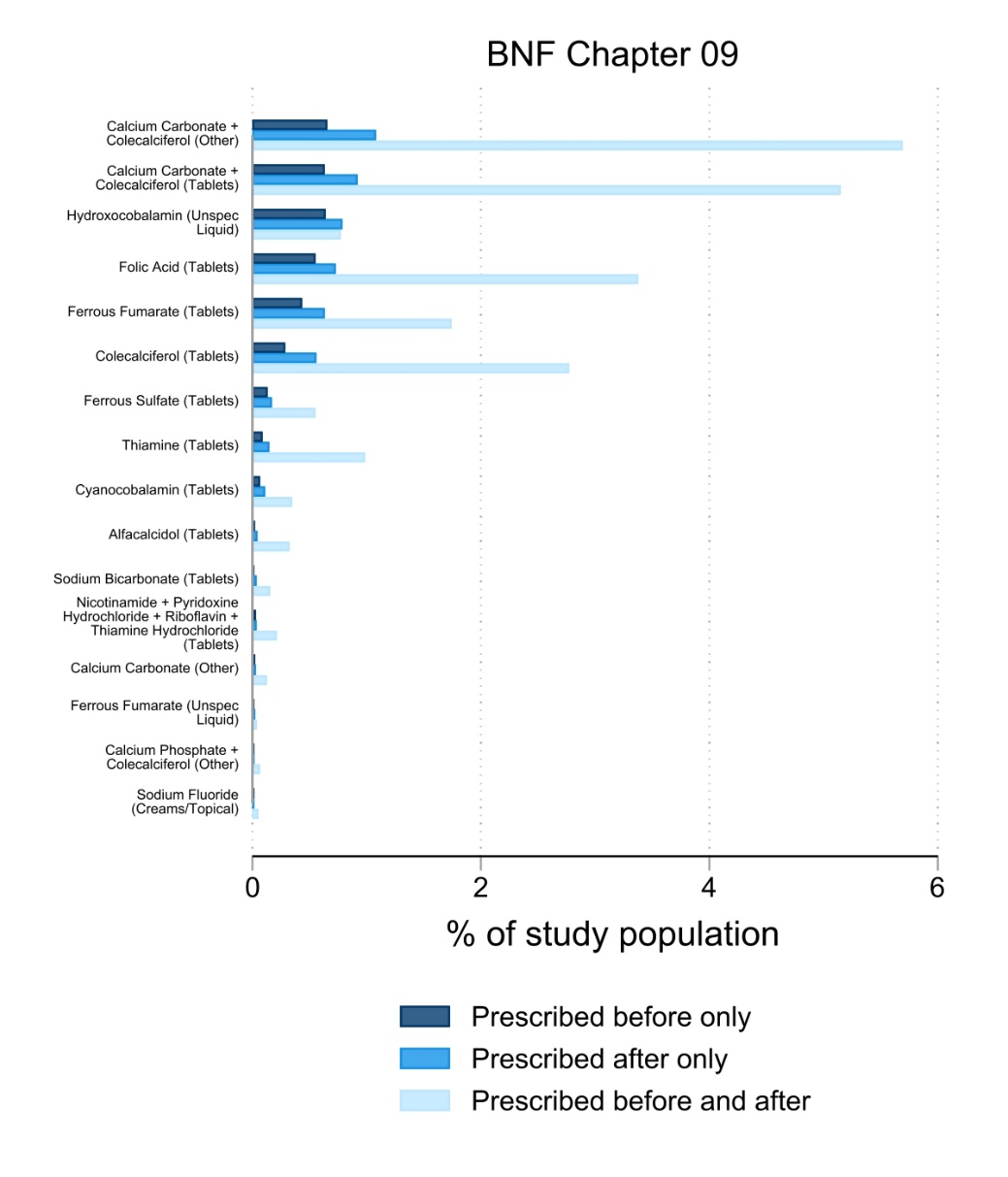


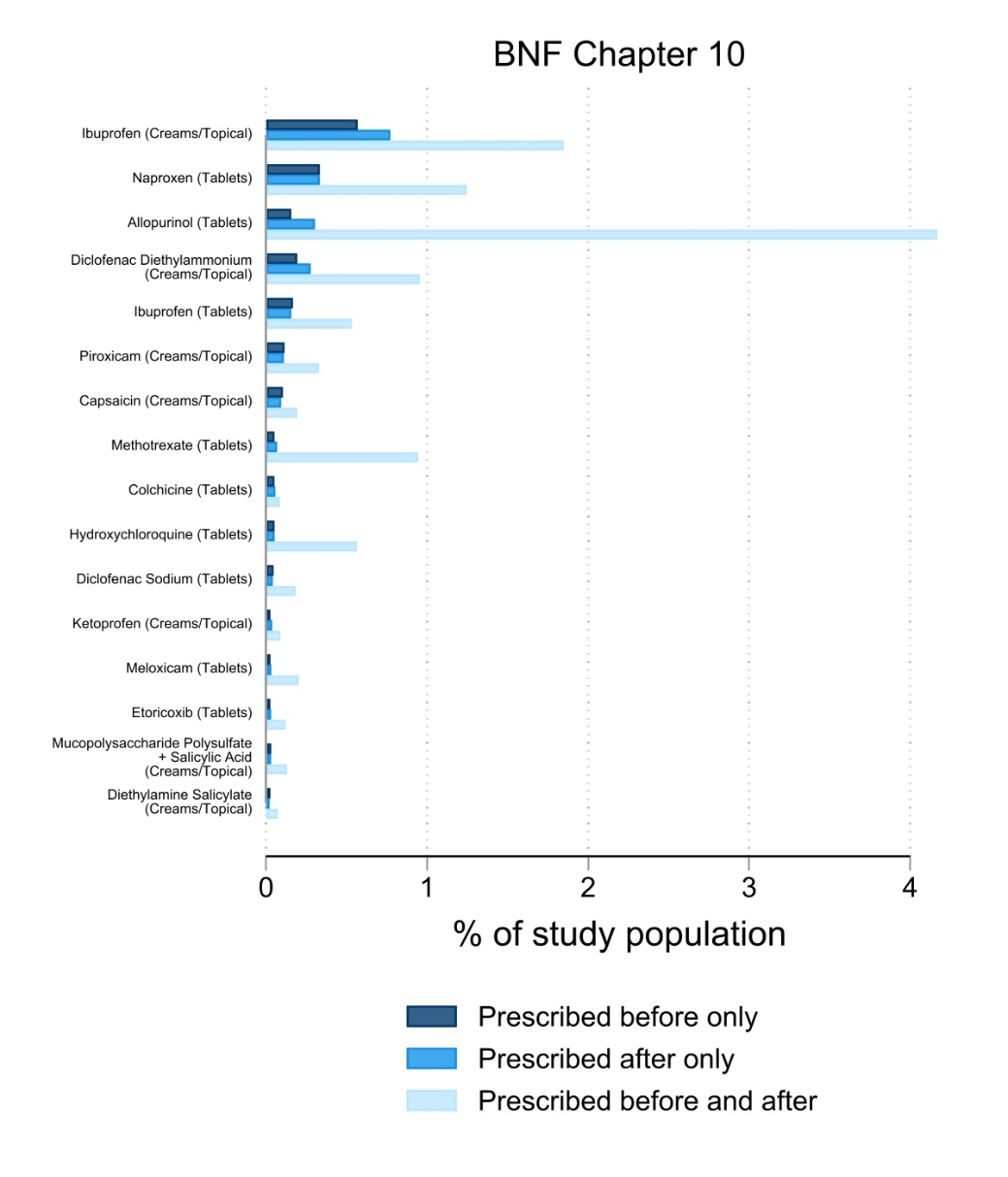


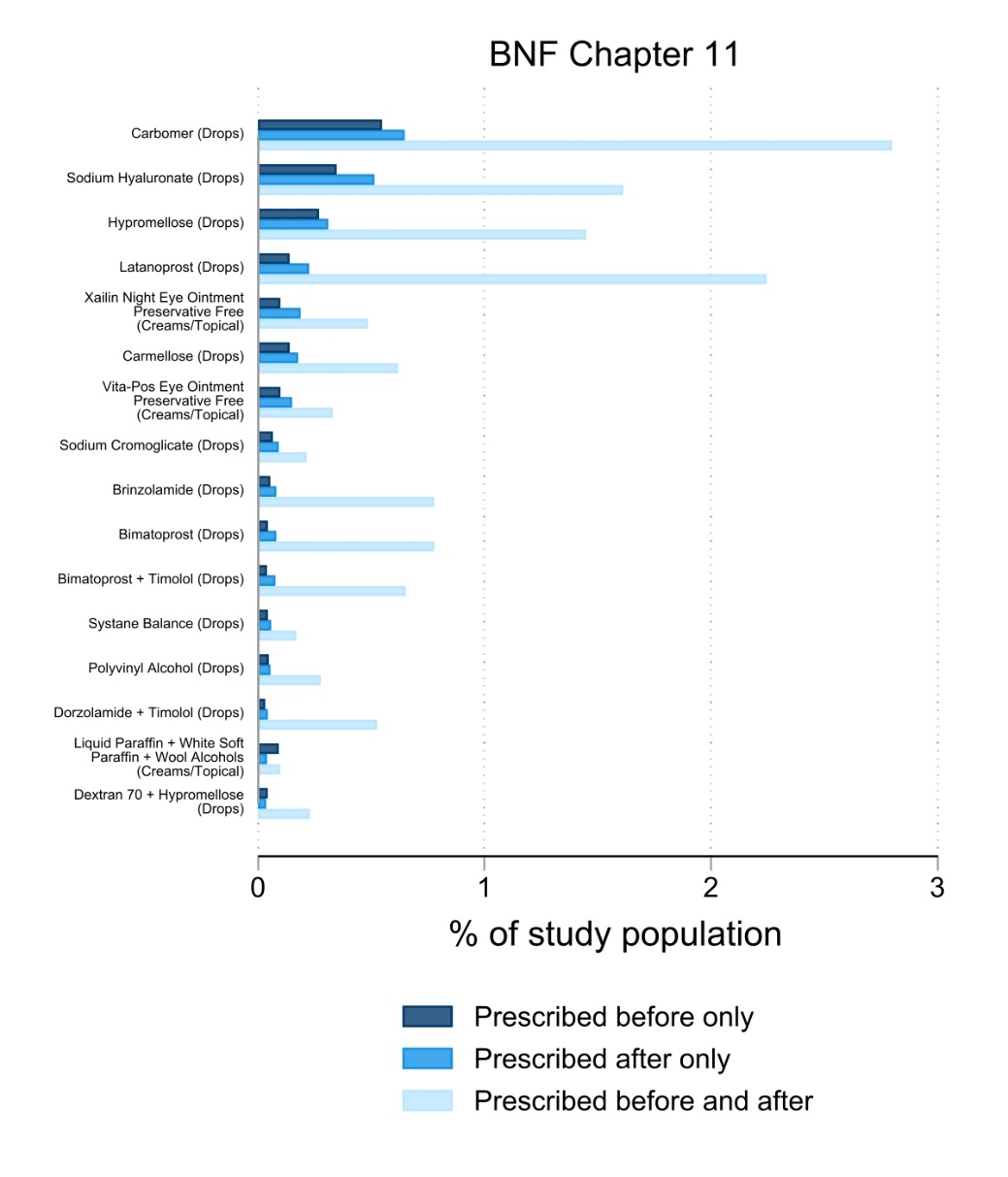


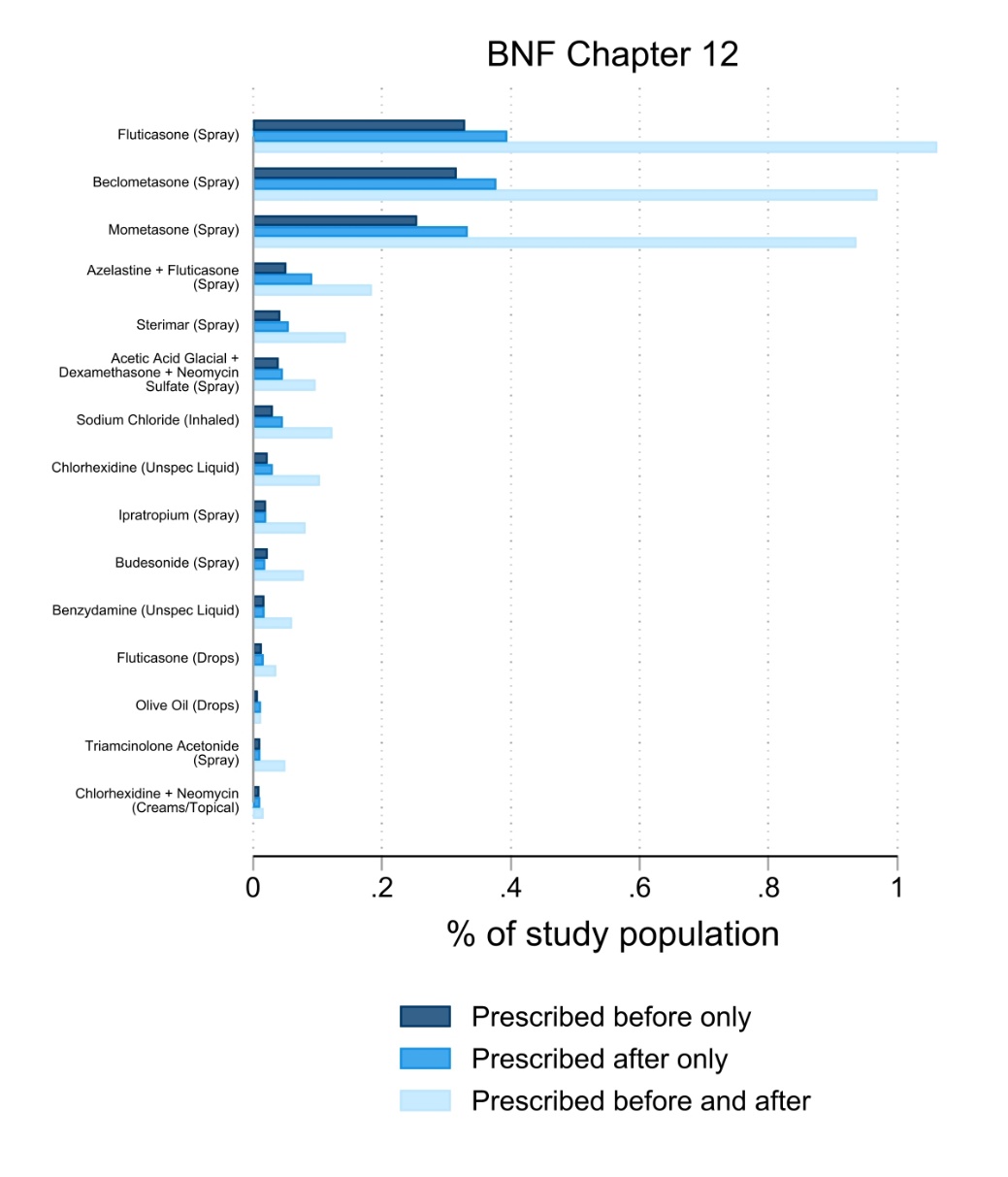


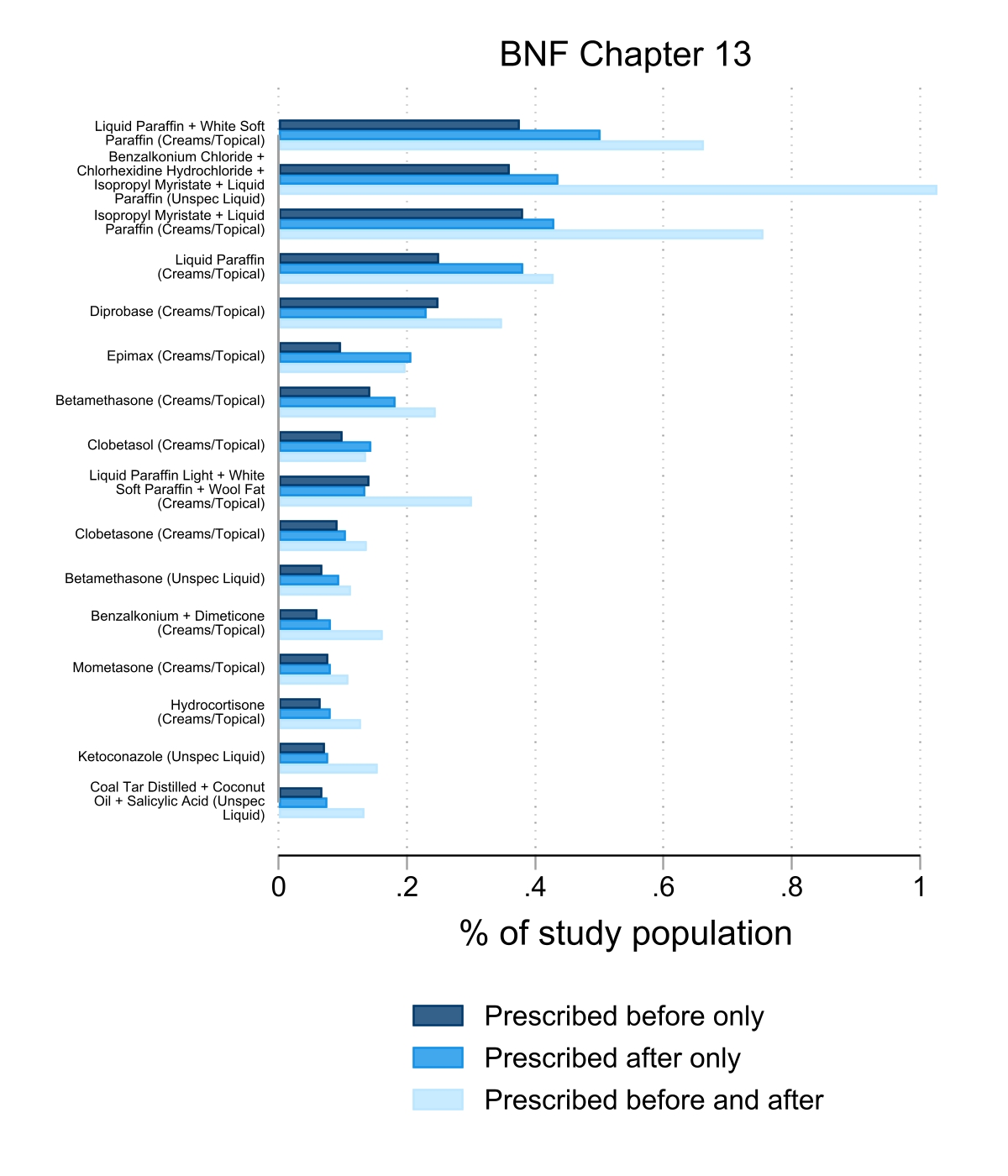


# Additional Figure S3.6. **Psychotropic medicines** prescribed in the three months before and/or after a medication review.

The figure is sorted according to the medicines most frequently ‘started’, i.e., prescribed only after the medication review. The top 20 most frequently ‘stopped’ or ‘started’ medicines are shown. Only prescriptions issued as a repeat prescription are included.


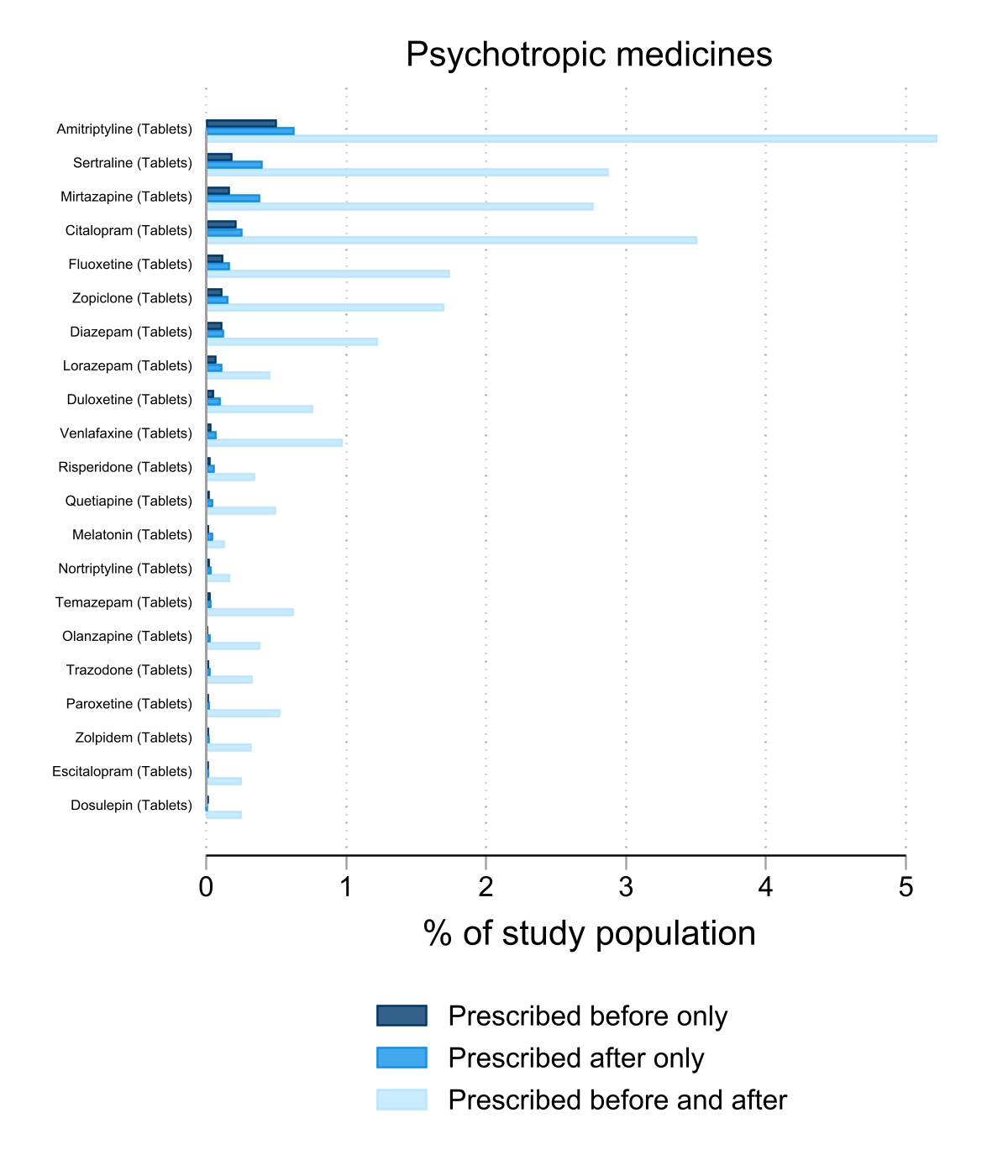


# Additional Figure S3.7. **Opioids** prescribed in the three months before and/or after a medication review.

The figure is sorted according to the medicines most frequently ‘started’, i.e., prescribed only after the medication review. The top 20 most frequently ‘stopped’ or ‘started’ medicines are shown. Only prescriptions issued as a repeat prescription are included.


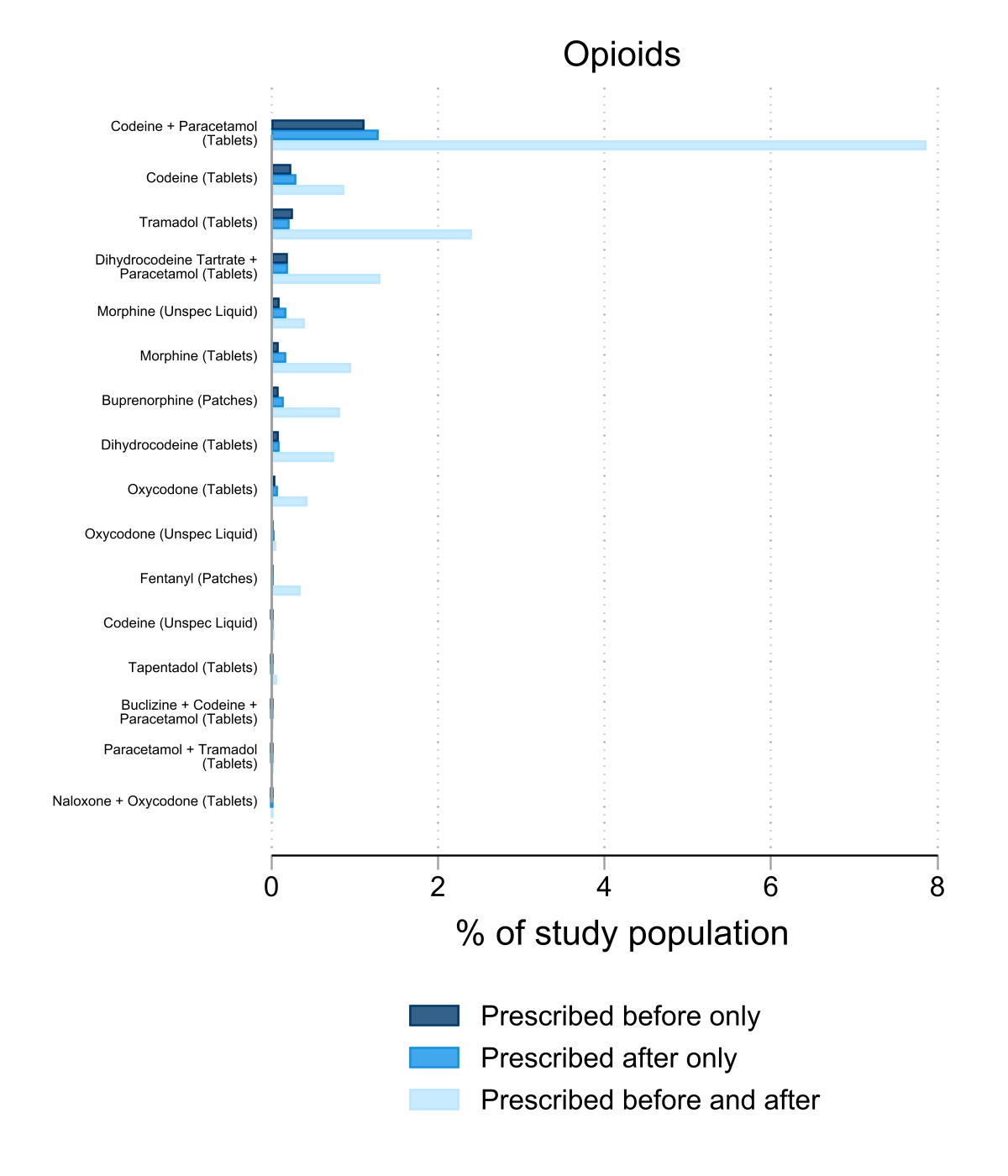


# Additional Figure S3.8. **Anticholinergic medicines** prescribed in the three months before and/or after a medication review.

The figure is sorted according to the medicines most frequently ‘started’, i.e., prescribed only after the medication review. The top 20 most frequently ‘stopped’ or ‘started’ medicines are shown. Only prescriptions issued as a repeat prescription are included.


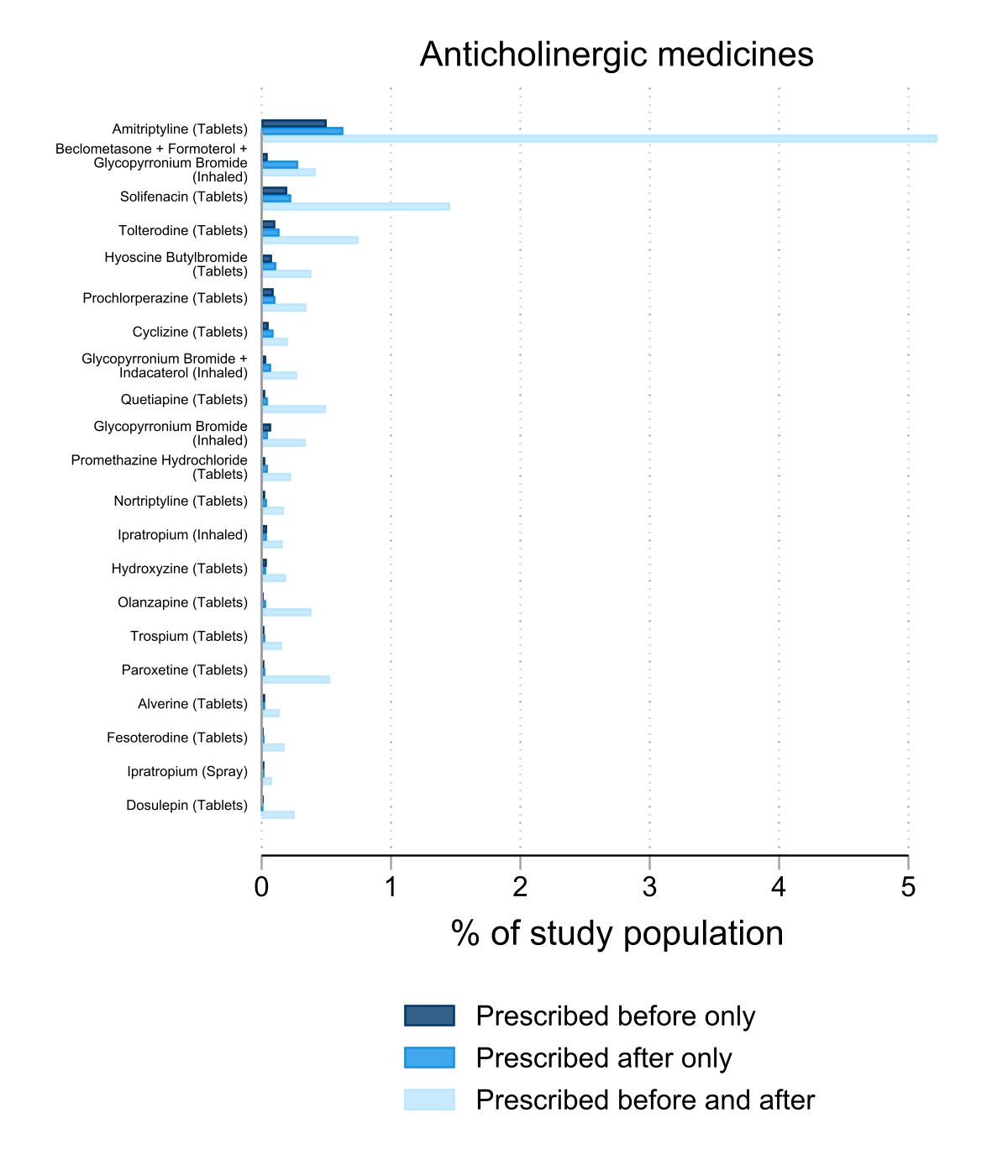


# Additional Figure S3.9. **Gabapentinoids** prescribed in the three months before and/or after a medication review.

The figure is sorted according to the medicines most frequently ‘started’, i.e., prescribed only after the medication review. The top 20 most frequently ‘stopped’ or ‘started’ medicines are shown. Only prescriptions issued as a repeat prescription are included.


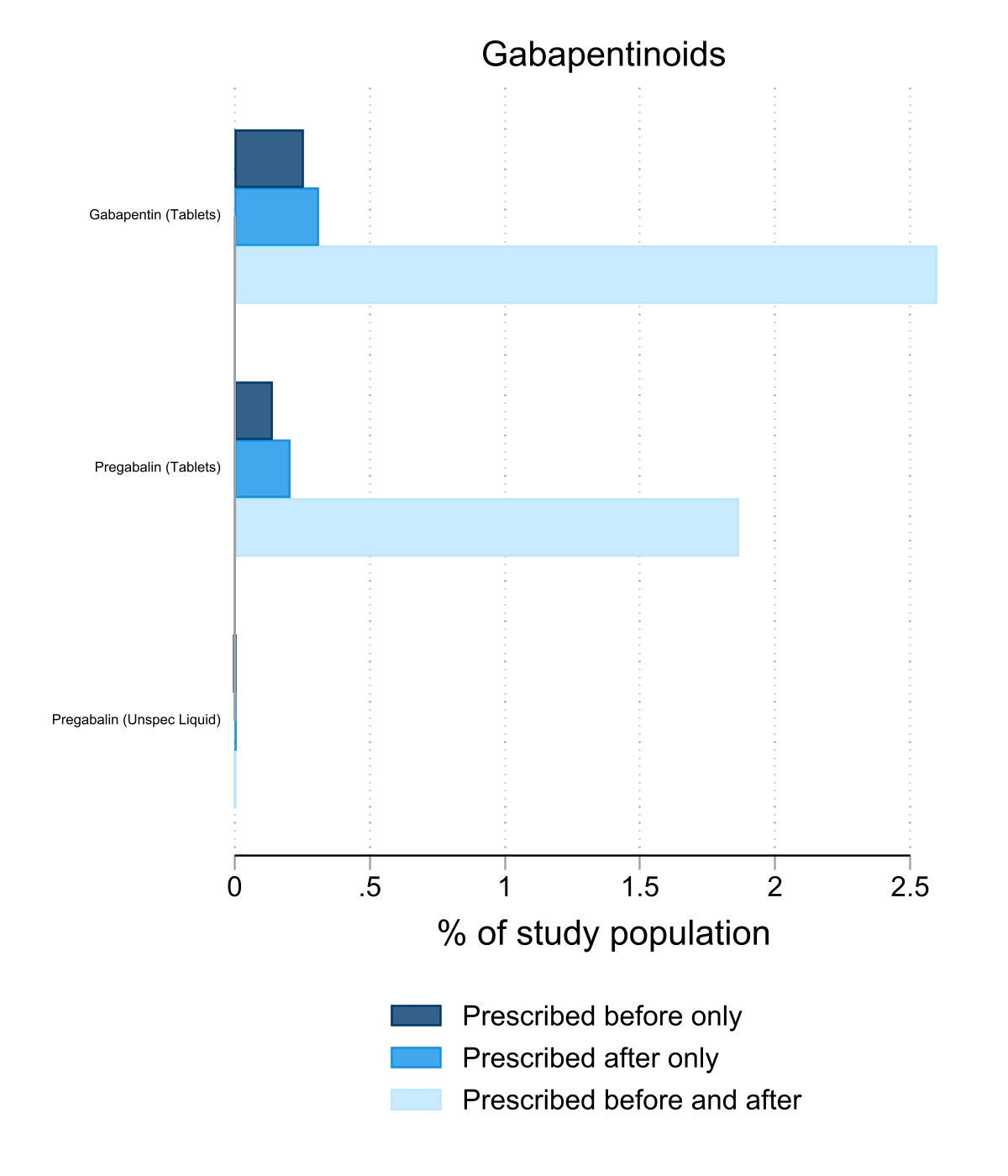


# Additional Figure S3.10. Medicines prescribed in the **six** **months** before and/or after a medication review.

The figure is sorted according to the medicines most frequently ‘started’, i.e., prescribed only after the medication review. The top 20 most frequently ‘stopped’ or ‘started’ medicines are shown. Only tablets and prescriptions issued as a repeat prescription are included.
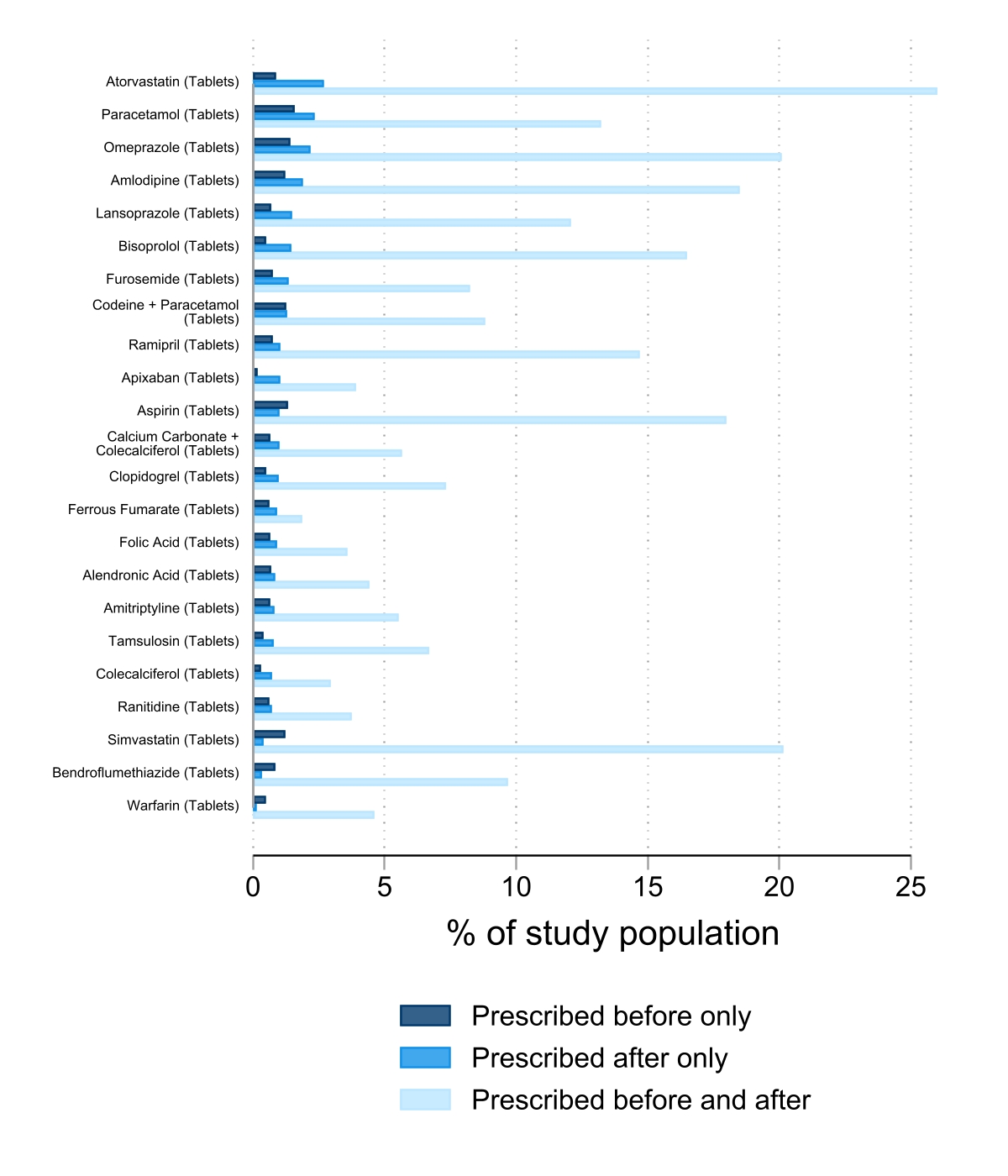


Additional Figure S3.11. Medicines prescribed in the **three months** before and/or **one-four** months after a medication review.

The figure is sorted according to the medicines most frequently ‘started’, i.e., prescribed only after the medication review. The top 20 most frequently ‘stopped’ or ‘started’ medicines are shown. Only tablets and prescriptions issued as a repeat prescription are included.


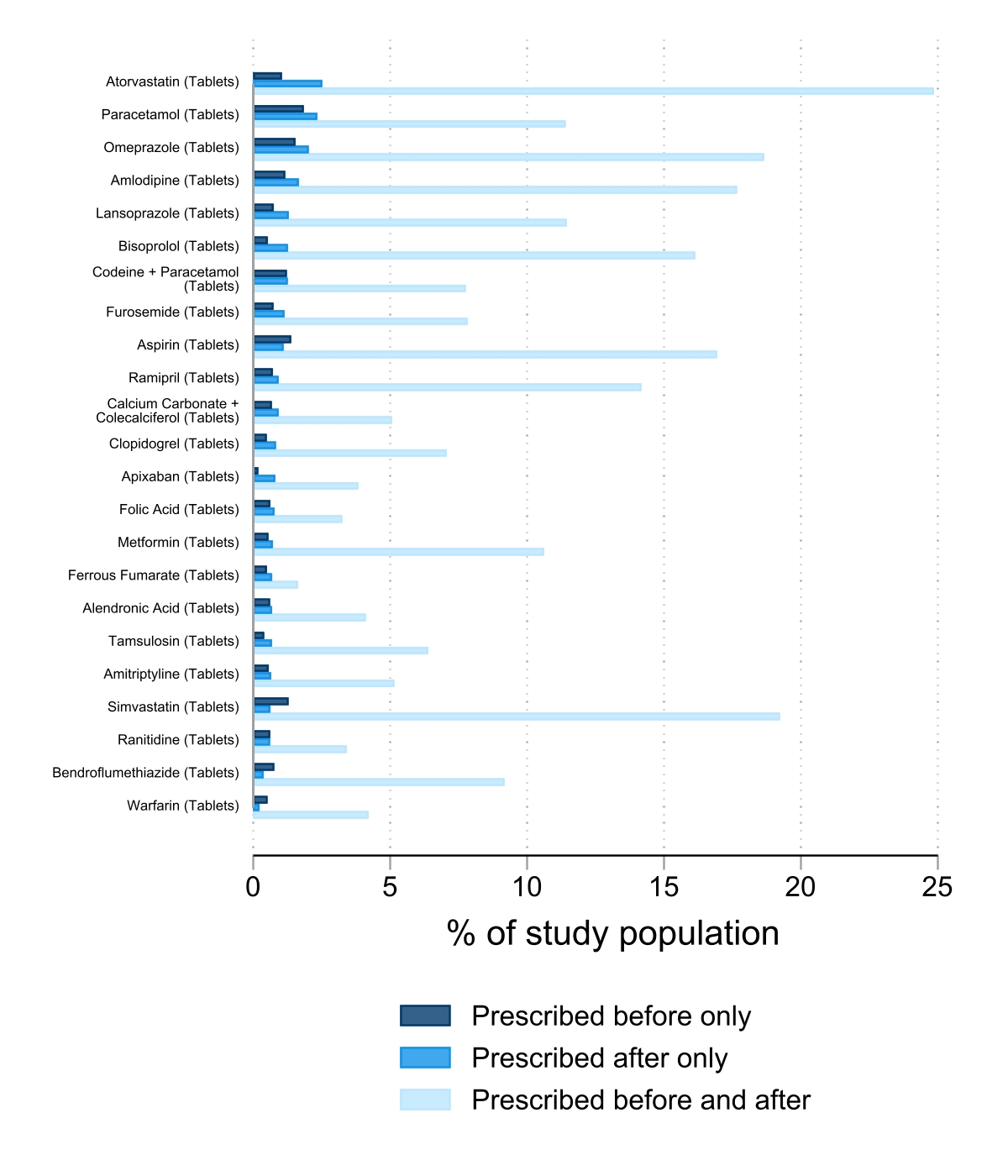


# Additional Figure S3.12. Medicines prescribed in the **one month** before and/or after a medication review.

The figure is sorted according to the medicines most frequently ‘started’, i.e., prescribed only after the medication review. The top 20 most frequently ‘stopped’ or ‘started’ medicines are shown. Only tablets and prescriptions issued as a repeat prescription are included.


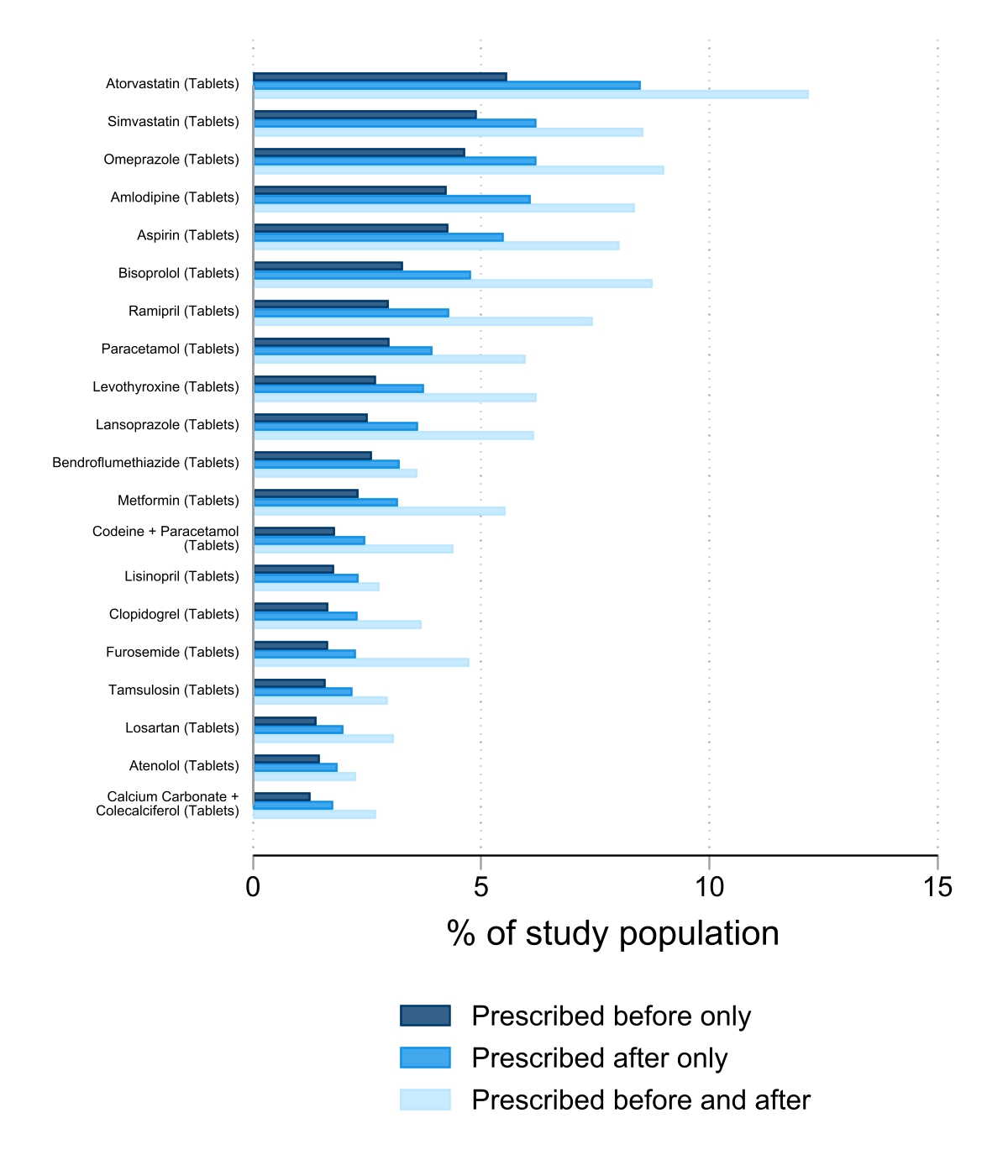


# Additional Figure S3.13. Medicines prescribed in the three months before and/or after an **in-person** medication review.

The figure is sorted according to the medicines most frequently ‘started’, i.e., prescribed only after the medication review. The top 20 most frequently ‘stopped’ or ‘started’ medicines are shown. Only tablets and prescriptions issued as a repeat prescription are included.


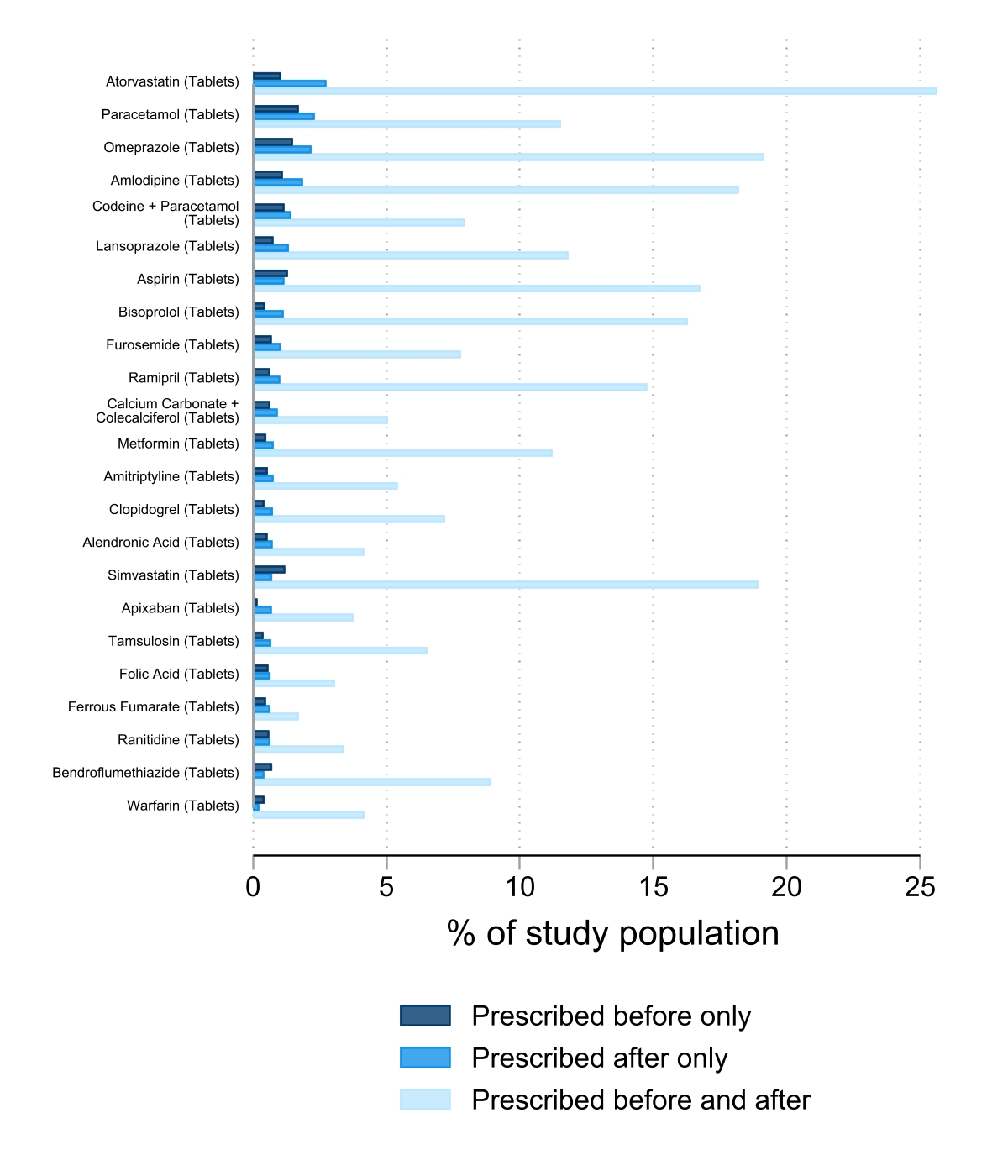


# Additional Figure S3.14. Medicines prescribed in the three months before and/or after a medication review – **most frequently prescribed**.

Sorted according to the most frequently prescribed medicines in the period after the medication review. The top 20 most frequently prescribed medicines before and/or after a medication review are shown. Only prescriptions issued as a repeat prescription are included.


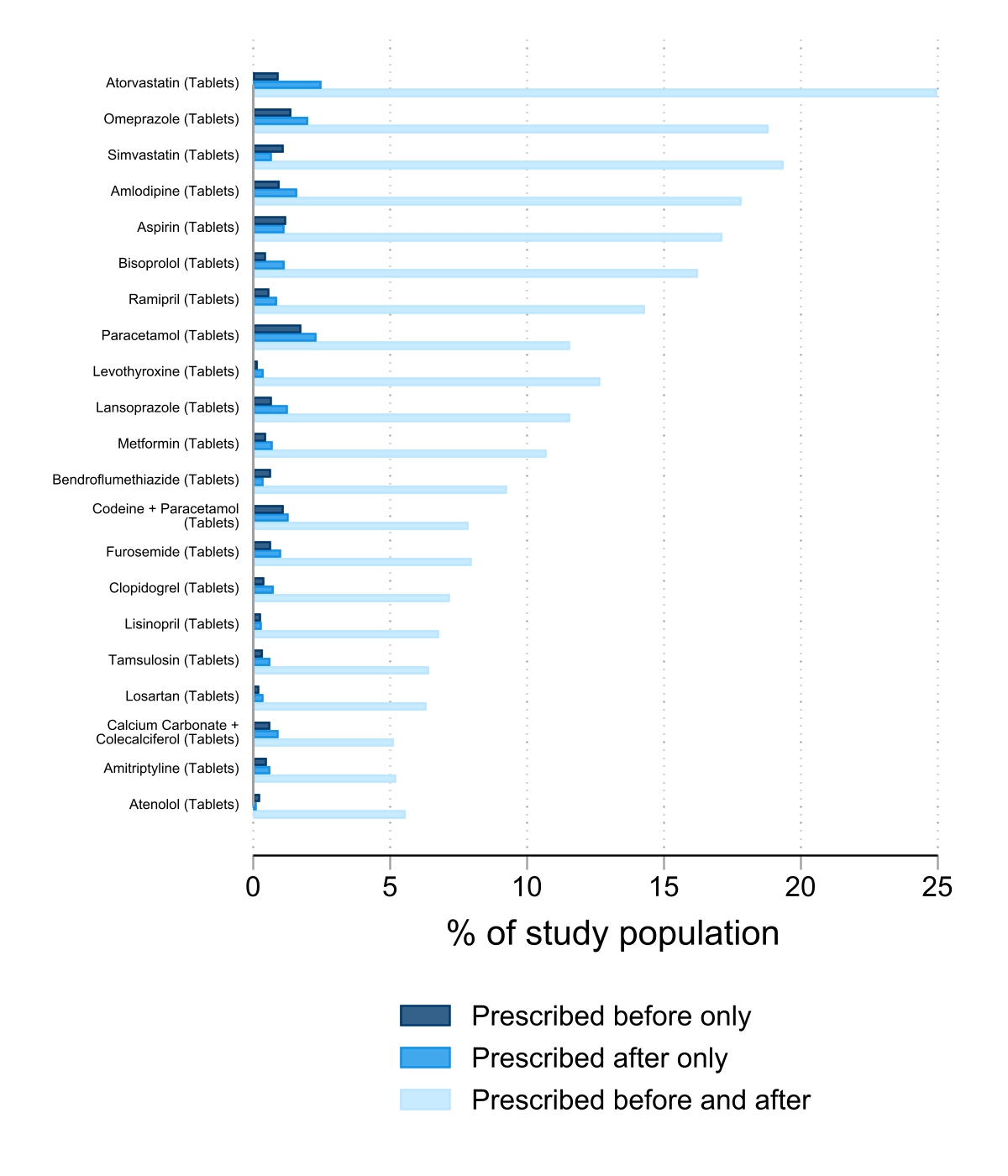


# Additional Figure S3.15. Medicines prescribed in the three months before and/or after a medication review – **most frequently prescribed, BNF paragraph-level**.

Sorted according to the most frequently prescribed medicines in the period after the medication review. The top 20 most frequently prescribed medicines before and/or after a medication review are shown. Only prescriptions issued as a repeat prescription are included. BNF British National Formulary.


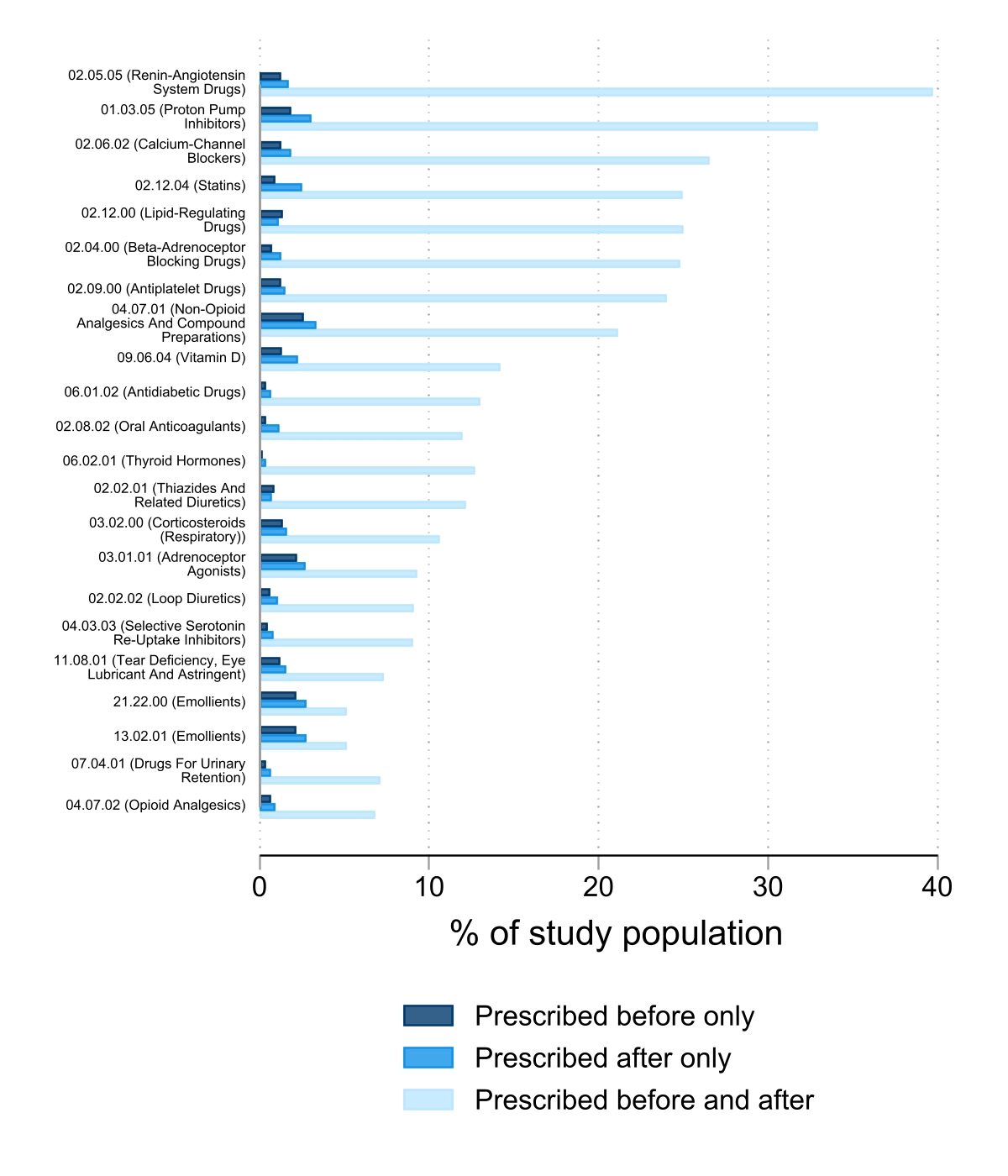


# Additional Figure S3.16. Medicines prescribed in the three months before and/or after a medication review – **most frequently prescribed, BNF chapter-level**.

Sorted according to the most frequently prescribed medicines in the period after the medication review. Only prescriptions issued as a repeat prescription are included. BNF British National Formulary.


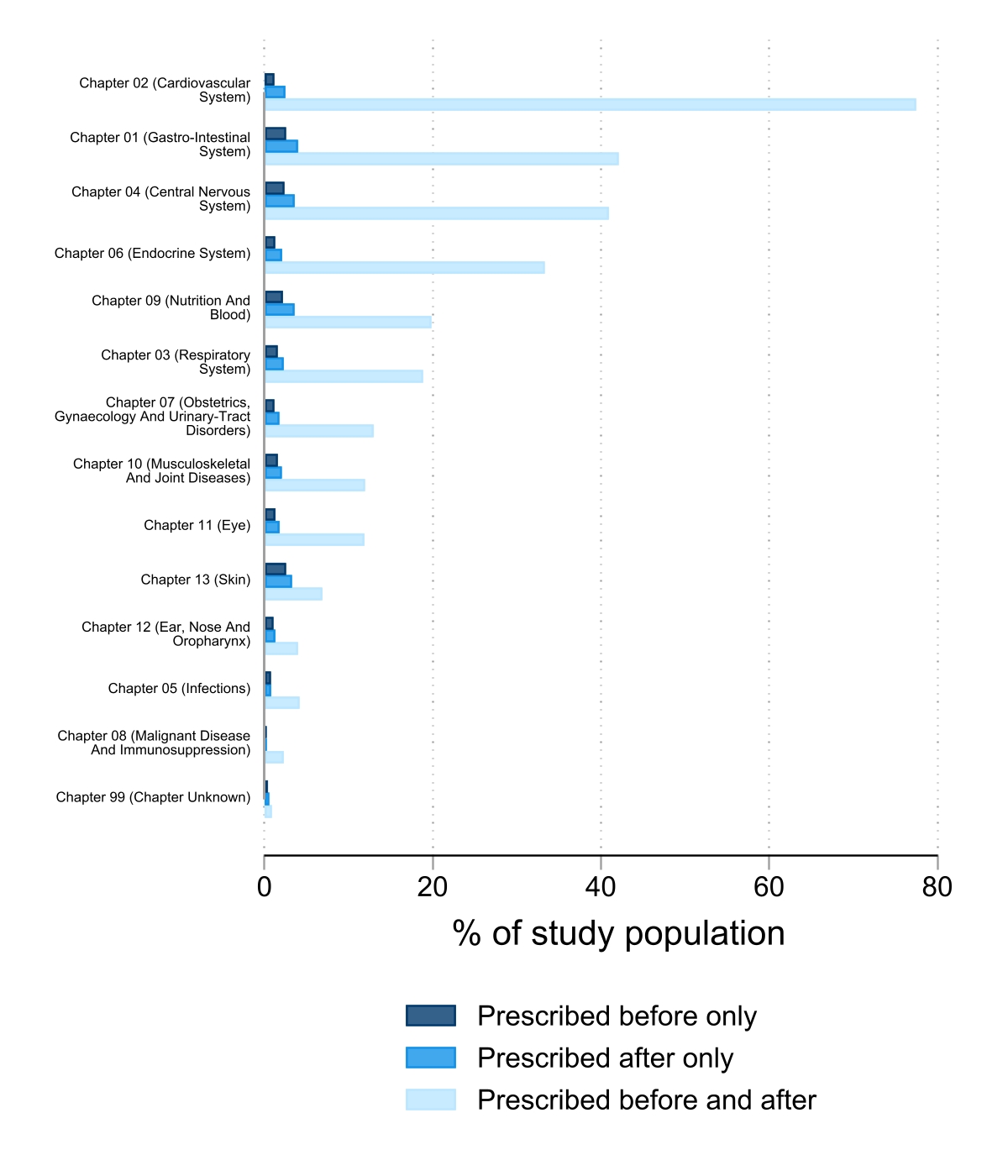

Supplement: Supplementary file 3 — Additional file 3: Additional Figure S3.1. Medicines prescribed in the three months before and/or after a medication review – BNF paragraph-level. Additional Figure S3.2. Medicines prescribed in the three months before and/or after a medication review – BNF chapter-level. Additional Figure S3.3. Medicines prescribed in the three months before and/or after a medication review – all formulations. Additional Figure S3.4. Medicines prescribed in the three months before and/or after a medication review – all prescription types. Additional Figure S3.5. Medicines prescribed in the three months before and/or after a medication review, by BNF chapter. Additional Figure S3.6. Psychotropic medicines prescribed in the three months before and/or after a medication review. Additional Figure S3.7. Opioids prescribed in the three months before and/or after a medication review. Additional Figure S3.8. Anticholinergic medicines prescribed in the three months before and/or after a medication review. Additional Figure S3.9. Gabapentinoids prescribed in the three months before and/or after a medication review. Additional Figure S3.10. Medicines prescribed in the six months before and/or after a medication review. Additional Figure S3.11. Medicines prescribed in the three months before and/or one-four months after a medication review. Additional Figure S3.12. Medicines prescribed in the one month before and/or after a medication review. Additional Figure S3.13. Medicines prescribed in the three months before and/or after an in-person medication review. Additional Figure S3.14. Medicines prescribed in the three months before and/or after a medication review – most frequently prescribed. Additional Figure S3.15. Medicines prescribed in the three months before and/or after a medication review – most frequently prescribed, BNF paragraph-level. Additional Figure S3.16. Medicines prescribed in the three months before and/or after a medication review – most frequently prescribed, BNF chapter-le [file 12877_2023_4143_MOESM3_ESM.docx]
